# DNA barcoding reveals ongoing immunoediting of clonal cancer populations during metastatic progression and immunotherapy response

**DOI:** 10.1038/s41467-022-34041-x

**Published:** 2022-11-07

**Authors:** Louise A. Baldwin, Nenad Bartonicek, Jessica Yang, Sunny Z. Wu, Niantao Deng, Daniel L. Roden, Chia-Ling Chan, Ghamdan Al-Eryani, Damien J. Zanker, Belinda S. Parker, Alexander Swarbrick, Simon Junankar

**Affiliations:** 1 Cancer Ecosystems Program, Garvan Institute of Medical Research, Darlinghurst, NSW 2010 Australia; 2 School of Clinical Medicine, Faculty of Medicine and Health, UNSW Sydney, Sydney, NSW 2052 Australia; 3 Sir Peter MacCallum Department of Oncology, University of Melbourne, Parkville, VIC 3010 Australia; 4 Cancer Immunology and Therapeutics Programs, Peter MacCallum Cancer Centre, Melbourne, VIC 3000 Australia

**Keywords:** Breast cancer, Immunoediting

## Abstract

Cancers evade the immune system through the process of cancer immunoediting. While immune checkpoint inhibitors are effective for reactivating tumour immunity in some cancer types, many other solid cancers, including breast cancer, remain largely non-responsive. Understanding how non-responsive cancers evade immunity and whether this occurs at the clonal level will improve immunotherapeutic design. Here we use DNA barcoding to track murine mammary cancer cell clones during immunoediting and determine clonal transcriptional profiles that allow immune evasion following anti-PD1 plus anti-CTLA4 immunotherapy. Clonal diversity is significantly restricted by immunotherapy treatment in both primary tumours and metastases, demonstrating selection for pre-existing breast cancer cell populations and ongoing immunoediting during metastasis and treatment. Immunotherapy resistant clones express a common gene signature associated with poor survival of basal-like breast cancer patient cohorts. At least one of these genes has an existing small molecule that can potentially be used to improve immunotherapy response.

## Abstract (web summary)

Understanding the molecular mechanisms of cancer immunoediting could provide insight into resistance to immunotherapy. Here, DNA barcoding provides evidence of ongoing immunoediting during metastasis and treatment with anti-PD1 and anti-CTLA4, and identifies cancer cell clones with unique immune evasive phenotypes.

## Introduction

All cancers must find ways to evade the immune system so that they can continue to grow^1^. Previous studies have established that this occurs through a process called immunoediting^2^. During immunoediting, more immunogenic cancer cells are selectively eliminated by the immune system, thus leaving behind less-immunogenic cancer cells that are then free to expand. Immunoediting can occur through multiple mechanisms, which include the elimination of cells with strong immunogenic mutations, leading to the loss of neo-antigens^3^, or the selection of cells with elevated expression of various immunosuppressive programmes^4^.

Immunotherapies look to overcome some of the immune evasion pathways established by cancer cells. The prominent clinically approved immunotherapies for solid tumours target T cell checkpoint molecules (eg. anti-CTLA4 and anti-PD1) to overcome T cell exhaustion^5,6^. In select cancer types, such as melanoma, immune checkpoint inhibitors have dramatic effects in a large proportion of patients^7^. Unfortunately for metastatic breast cancer, few patients, even those having the most sensitive basal-like breast cancer, had durable responses in clinical trials^8^. This indicates that in metastatic breast cancer, resistance is either pre-existing or can rapidly develop to anti-PD1/PD-L1 therapy and suggests that alternate immune drug targets are needed for breast cancer.

Although the immune system is known to play a role in breast cancer outcome^9^ and immunoediting can occur in a transgenic mouse model of breast cancer^10^, very little is known about immune evasion by breast cancer cells. The majority of studies examining immune evasion by cancers were performed using the highly mutated, methylcholanthrene-driven sarcoma model, the response of which cannot be tracked at the clonal level^11^, or colon cancer^12^. Of interest, a recent study suggests that immunoediting by T cells can occur at the clonal level by demonstrating the selection of clones that contain less-immunogenic fluorophores^13^. This leaves an important gap in our collective knowledge as to the mechanisms employed in less-immunogenic tumours such as breast.

Both natural killer (NK) cells and T cells have been demonstrated to play a role in immunoediting^14,15^. However, the majority of recent research has focused on pathways relevant to T cell recognition^16–20^. Downregulation of major histocompatibility complex (MHC) is one mechanism by which cancer clones become impervious to T cells^21^, but this inherently makes them targets of NK activity. In breast cancer, dysfunction of NK cells is noted and this is regulated by microenvironmental factors^22^. Data on resistance pathways that allow for immune evasion from both T cells and NK cells are currently more limited.

Intratumoural heterogeneity (ITH) has been identified as a major contributor to treatment response. Prior work in non-small cell lung cancer (NSCLC) demonstrated that neoantigen heterogeneity, and tumour mutation burden more broadly, is strongly associated with T cell anti-cancer immune responses^23,24^. Chemotherapy^25^ or loss of HLA^26^ have also been shown to increase ITH, which in turn was associated with treatment relapse in NSCLC. Similar results have been described in cell line models of melanoma^27^, with Williams and colleagues providing recent evidence of ITH enabling clonal cooperatively and immune escape in melanoma-derived cell lines^28^. While these previous studies have been crucial to our understanding of ITH and anti-tumour immune responses, there are limited studies of this nature in breast cancer. Furthermore, direct evidence of ITH enabling immune escape and immunotherapy resistance in vivo has not been reported.

Here we use DNA barcoding to analyse immunoediting in vivo in the primary tumours and resulting metastases and to study whether resistance to immune checkpoint inhibition develops from pre-existing or de novo-generated cell populations. We show pre-existing cell populations are selected for at the primary site, metastases and in the context of immunotherapy. By isolating immunotherapy-resistant clones, we identify multiple mechanisms of immune evasion are maintained in simultaneously in tumours. Furthermore, transcriptomic analysis reveals a common gene signature associated with poor survival in two cohorts of basal-like breast cancer.

## Results

### Immunoediting of breast cancer cells in the primary tumour

**Fig. 1:**
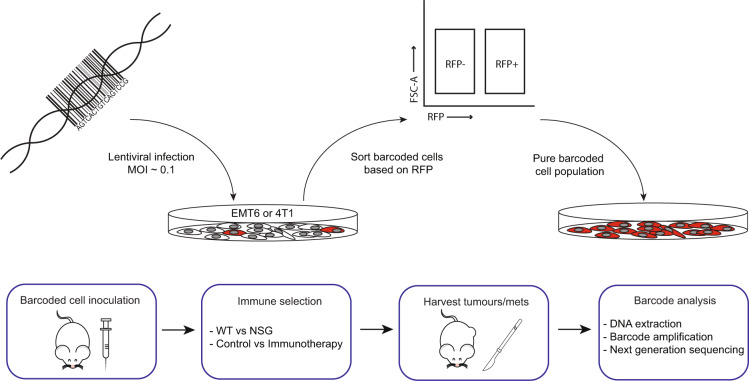
Experimental workflow schematic. Barcode library is introduced into mammary carcinoma cell lines (EMT6 or 4T1) in vitro at a low multiplicity of infection (MOI). Cells are sorted based on red fluorescent protein (RFP) expression to select those having incorporated a barcode. Barcoded cells are then transplanted into the mammary fat pad of immunocompromised (NSG) or immunocompetent (WT) mice. Following immunoselection with either the endogenous immune system or immunotherapy, barcode abundance and diversity within the primary tumours and lung-bearing metastases are analysed. Source data are provided as a source data file.

**Fig. 2:**
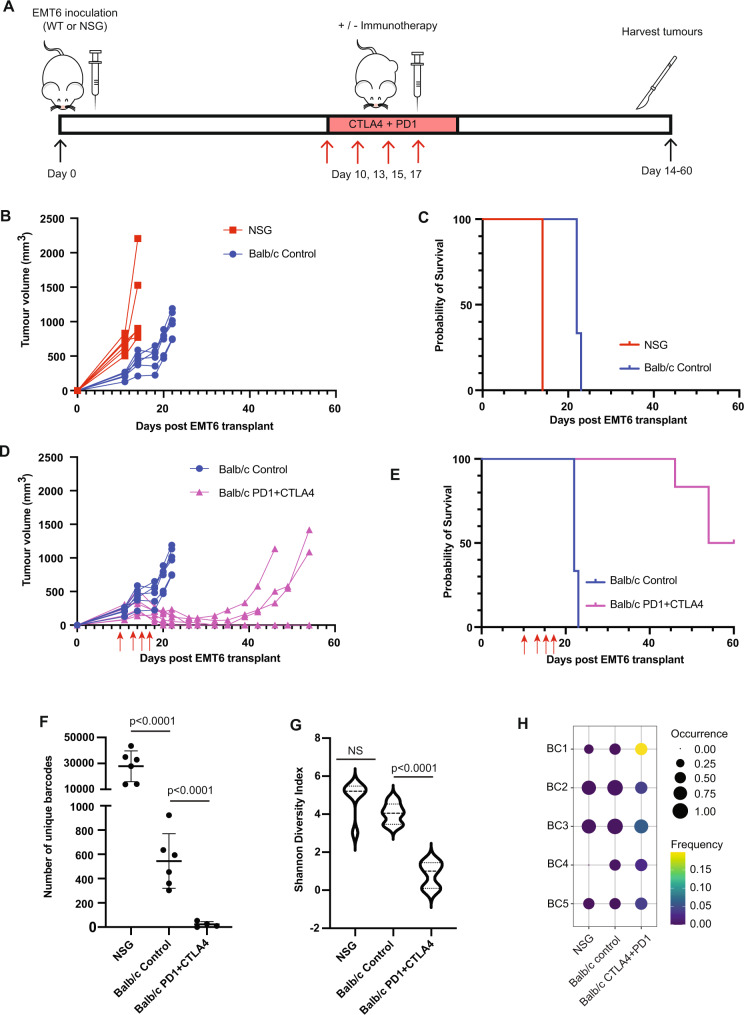
Immune selection and clonal immunoediting of EMT6 primary tumours. **A** Outline of experimental design. Wildtype (WT, Balb/c) and NOD scid gamma (NSG). **B** EMT6 primary tumour growth in wild-type Balb/c mice and in NSG mice plotted as tumour volume. Average volume ± SEM; *n* = 6 mice per group. **C** Kaplan–Meier survival analysis comparing Balb/c and NSG mice bearing EMT6 tumour (Mantel–Cox *p* = 0.009) *n* = 6 mice per group. **D**. EMT6 primary tumour growth plotted as tumour volume in wild-type Balb/c mice with or without immunotherapy (anti-PD1 + anti-CTLA4) on days 10, 12, 14 and 17 as indicated with red arrows. Average volume ± SEM; *n* = 5 immunotherapy-treated mice, 6 control-treated mice. **E**. Kaplan–Meier survival analysis of Balb/c mice bearing EMT6 tumours treated with immunotherapy or isotype control (Mantel–Cox *p* = 0.0006). *n* = 5 immunotherapy-treated mice, 6 control-treated mice. **F**. Number of unique barcodes identified in EMT6 primary tumours grown in NSG mice and in Balb/c mice treated with isotype control antibodies or anti-PD1 + anti-CTLA4. GLM fit with Tukey’s HSD for multiple comparisons. *n* = 6 mice (NSG), 6 mice (Balb/c control), 5 mice (Balb/c PD1 + CTLA4). Data presented as mean ±  SD. **G**. Shannon diversity index analysis of EMT6 primary tumours grown in NSG mice and in Balb/c mice treated with isotype control antibodies or anti-PD1 + anti-CTLA4. *p* value = 0.00001. One-way ANOVA with Tukey’s HSD for multiple comparisons. *n* = 6 mice (NSG), 6 mice (Balb/c control), 5 mice (Balb/c PD1 + CTLA4). **H** Dot plot of a subset of barcodes with enrichment following immunotherapy treatment. *n* = 6 mice (NSG), 6 mice (Balb/c control), 5 mice (Balb/c PD1 + CTLA4). Source data are provided as a source data file.

To understand the role of the immune system and immunotherapy in shaping the clonal dynamics of cancer cells within primary tumours, we used a DNA barcoding approach (Fig. 1). We introduced the ClonTracer DNA barcode library that contains ~7 million unique barcodes^29^ into the immunotherapy-sensitive mouse mammary carcinoma EMT6 cells^30^ (Sup Fig. 1), resulting in ~41,000 unique barcodes identified by DNA sequencing (Sup Fig. 2A). We then inoculated 250,000 cells (~6-fold over-representation of each barcode) into the mammary fat pad of syngeneic immune-competent wild-type (WT) Balb/c mice or severely immunocompromised NOD SCID Gamma (NSG) mice that lack T cells, B cells and functional NK cells, and compared the number of clones that were able to engraft and grow (Fig. 2A and Sup Fig. 2, and Sup Table 1).

Tumour growth was faster in NSG mice than in WT mice, with tumours reaching an ethical endpoint in NSG mice on day 14 post-transplant and in WT mice by day 23 (Fig. 2B), leading to NSG mice having significantly shorter median overall survival (14 days) than WT mice (22 days, Mantel–Cox *p* = 0.009). These results suggest that the immune system plays an important role in controlling the growth of the EMT6 primary tumours (Fig. 2C).

To examine the influence of immunotherapy on tumour growth and clonal dynamics, we compared WT mice treated with immunotherapy (anti-PD1 + anti-CTLA4) or control antibodies starting from day 10 when tumours were ~200 mm^3^ (Fig. 2A). Tumours in all mice treated with control antibodies reached ethical endpoint by day 23 (Fig. 2D). In contrast, tumours in all mice treated with immunotherapy regressed following treatment, with 50% relapsing and reaching ethical endpoint between days 46 and 54 (Fig. 2D). The remaining mice treated with immunotherapy remained tumour free until the experiment was terminated on day 60. During harvest, a small residual lesion was observed in two of these mice, but no metastatic lesions were observed in any of the mice, irrespective of treatment. Kaplan–Meier analysis demonstrated that immunotherapy significantly increased the median survival from 22 days to 57 days (Mantel–Cox *p* = 0.0006) (Fig. 2E).

To determine if immune control of tumour growth was driven at a clonal level, we examined the number and distribution of barcodes present in primary tumours collected at an ethical endpoint in the experiment described above. We found that at an ethical endpoint, tumours grown in NSG mice had over 50 times the number of unique barcodes as tumours grown in control WT mice (*p* < 0.0001, generalised linear model (GLM) with Tukey’s correction), which in turn had more than 20 times the number of unique barcodes found in WT mice treated with immunotherapy (*p* < 0.0001, GLM with Tukey’s correction) (Fig. 2F and Sup Fig. 2A). We applied Shannon diversity analysis to understand how the immune system influenced the diversity of barcodes in these samples. Shannon diversity index determines how evenly distributed the barcodes are within a population and is only moderately influenced by barcode number. Analysis of barcode diversity revealed a trend to a lower barcode diversity in tumours from control (WT Balb/c) mice than that in tumours from NSG mice, whereas the barcode diversity was significantly lower in immunotherapy treated than in control-treated Balb/c mice (Fig. 2G, *p* < 0.001 one-way ANOVA with Tukey’s correction). These data suggest that a subset of EMT6 cells are more resistant to the endogenous immune system, but this selection does not skew the evenness of the barcode distribution significantly, which suggests that all clones resistant to the immune system have similar levels of resistance. In contrast, immunotherapy applies a more stringent bottleneck that only a limited number of clones can overcome, and with a high degree of variability in doing so. Further analysis identified EMT6 clones that were reproducibly enriched across multiple mice following immunotherapy treatment, indicating that they had a pre-existing resistance phenotype that was being positively selected for (Fig. 2H).

### Immunoediting of breast cancer cells during metastasis

**Fig. 3:**
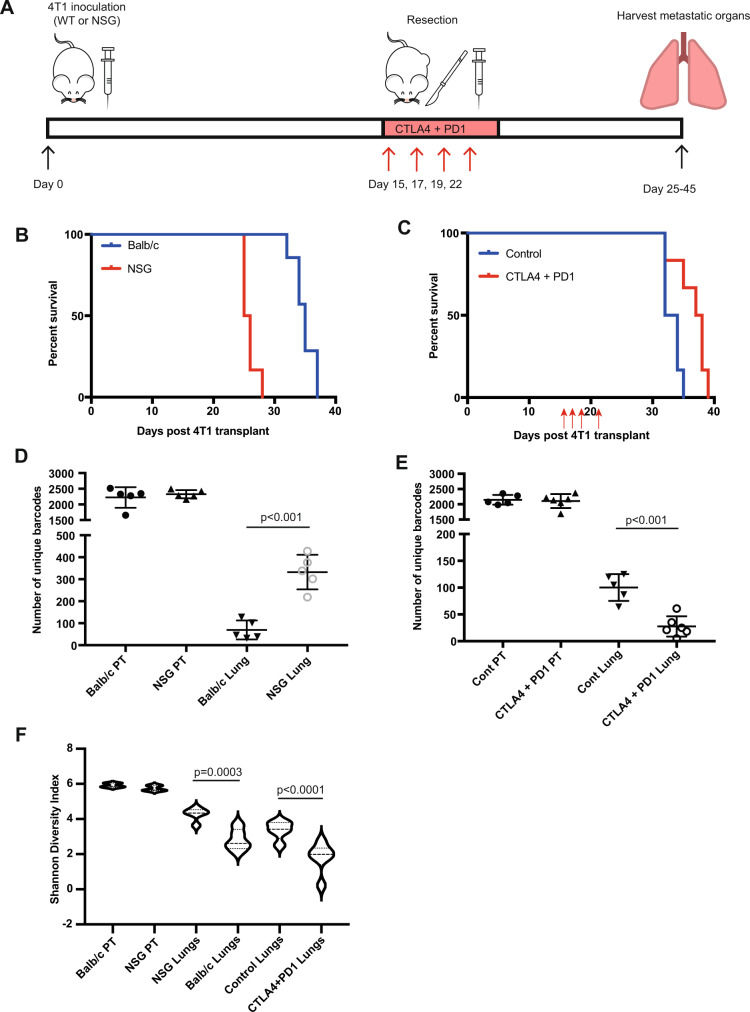
Immune selection and clonal immunoediting in the metastatic setting utilising the 4T1 model. **A** Outline of experimental design. Wildtype (WT), NOD scid gamma (NSG). **B** Kaplan–Meier survival analysis comparing Balb/c mice and NSG mice bearing 4T1 tumour; *n* = 5 mice/group (*p* = 0.0002, Mantel–Cox log-rank test). **C** Kaplan–Meier survival analysis of Balb/c mice bearing 4T1 tumour treated with immunotherapy or isotype control on days 15, 17, 19 and 21 as indicated with red arrows; *n* = 5 mice/group (*p* = 0.0141, Mantel–Cox log-rank test). **D** Number of unique barcodes identified in 4T1 primary tumours (PT) and lung metastases grown in NSG mice or Balb/c mice. GLM fit with Tukey’s HSD for multiple comparisons. *n* = 5 mice per group. Data are shown as mean ± SD. **E** Number of unique barcodes identified in 4T1 primary tumours (PT) and lung metastases grown in Balb/c mice treated with isotype control antibodies or anti-PD1 + anti-CTLA4. GLM fit with Tukey’s HSD for multiple comparisons. *n* = 5 mice per group. Data are shown as mean ±  SD. **F** Shannon diversity index analysis in primary tumours (PT) from NSG mice or Balb/c mice and in lung metastases from NSG mice, Balb/c mice, Balb/c mice treated with isotype control and Balb/c mice treated with anti-PD1 + anti-CTLA4. *P* value = 0.00005 for control lungs vs CTLA4 + PD1 lungs. One-way ANOVA with Tukey’s HSD for multiple comparisons. Five mice per group. Source data are provided as a source data file.

To determine whether immunoediting continued during metastatic dissemination and whether specific metastatic clones were enriched or depleted, we turned to the highly metastatic 4T1 mammary carcinoma model, as the EMT6 cell line is poorly metastatic^31^. We introduced the barcode library into 4T1 cells, leading to a cell pool with ~5000 unique barcodes (Sup Figs. 3, 4), then inoculated 50,000 of these cells (a ~10-fold over-representation of each barcode) into the mammary fat pad of WT and NSG mice (Sup Fig. 4). We resected primary tumours 15 days following inoculation to allow metastases to develop (Fig. 3A). All mice developed lethal lung metastases, with NSG mice succumbing to the metastatic disease earlier than WT mice (median survival of 25.5 days versus 35 days, *p* = 0.0002, Mantel–Cox log-rank test) (Fig. 3B). Primary tumour sizes at the time of resection were similar between the groups (Sup Fig. 5A). Adjuvant immunotherapy with anti-PD1 + anti-CTLA4 led to a modest but significant increase in survival (37.5 days) versus control-treated mice (33 days; *p* = 0.0121, Mantel–Cox log-rank test) (Fig. 3C).

We then examined whether the endogenous immune system shaped metastatic clonal dynamics. While primary tumours contained similar numbers of clones and barcode diversity in NSG and WT hosts (Fig. 3D and Sup Fig. 5B, C), the lung metastases of NSG mice contained approximately three times as many barcode clones as those of WT controls (Fig. 3D). We next determined if the increase in survival following immunotherapy was associated with alterations in clonal dynamics. As the treatment was only given after the excision of the primary tumour, the immunotherapy would only affect the outgrowth of cancer cells that had already metastasised to the lung. Despite only a modest increase in survival following combination immunotherapy (Fig. 3C), we observed a 70% reduction in the number of clones in metastases (Fig. 3E).

The higher barcode number in the lung metastases of NSG mice than in the lung of WT mice was associated with a higher diversity of barcodes as measured using the Shannon diversity index (Fig. 3F). This shows that the endogenous immune system restricts the number and skews the diversity of metastatic clones that can reach and outgrow in the lungs. In addition to the reduction in barcode number following immunotherapy treatment, we also saw a significant reduction in barcode diversity (Fig. 3F). This suggests that immunotherapy is leading to the immunoediting of specific clonal cell populations over others.

To further understand the key immune cell types that control clonal outgrowth in the lung, we depleted CD4 T cells using anti-CD4, CD8 T cells using anti-CD8 or NK cells using anti-asialo-GM1 in wild-type mice starting one day prior to tumour resection (Sup Fig. 6B–D). None of these treatments led to a significant change in overall survival (Sup Fig. 6A). Initial experiments indicated a small change in the number of clones detected within the lung between treatment groups (Sup Fig. 6E). However, this was not recapitulated in repeat experiments (Sup Fig. 6F). This may suggest that none of these cell types alone are sufficient to restrict clonal diversity after seeding of pulmonary metastases.

We were surprised by the lack of immunoediting in the primary tumours of the 4T1 model, so we repeated these experiments with a second pool of barcoded 4T1 cells containing a larger barcode library (300,000 barcodes). Following the injection of 50,000 barcoded cells, we recovered approximately 10,000−12,000 barcode sequences from each primary tumour grown in WT mice and in NSG mice (Sup Fig. 8A). This suggests that roughly a fifth of the injected cells are able to engraft and grow in the mammary gland. As clone diversity was again similar between NSG mice and WT mice, this confirms that the immune system does not play a major role in restricting the growth of 4T1 cells in the primary tumour setting. In contrast, when we examined the number of clones that had spread to the lung, we again found approximately three times as many in NSG mice as in wild-type mice (Sup Fig. 8A). In addition, we similarly saw an approximately threefold reduction in barcode diversity in response to immunotherapy (Sup Fig. 8B). These results confirm that the 4T1 cell line is already highly immunoedited for growing at the primary site yet is subjected to the second round of immunoediting during metastasis.

### Patterns of enrichment and depletion of specific clones

**Fig. 4:**
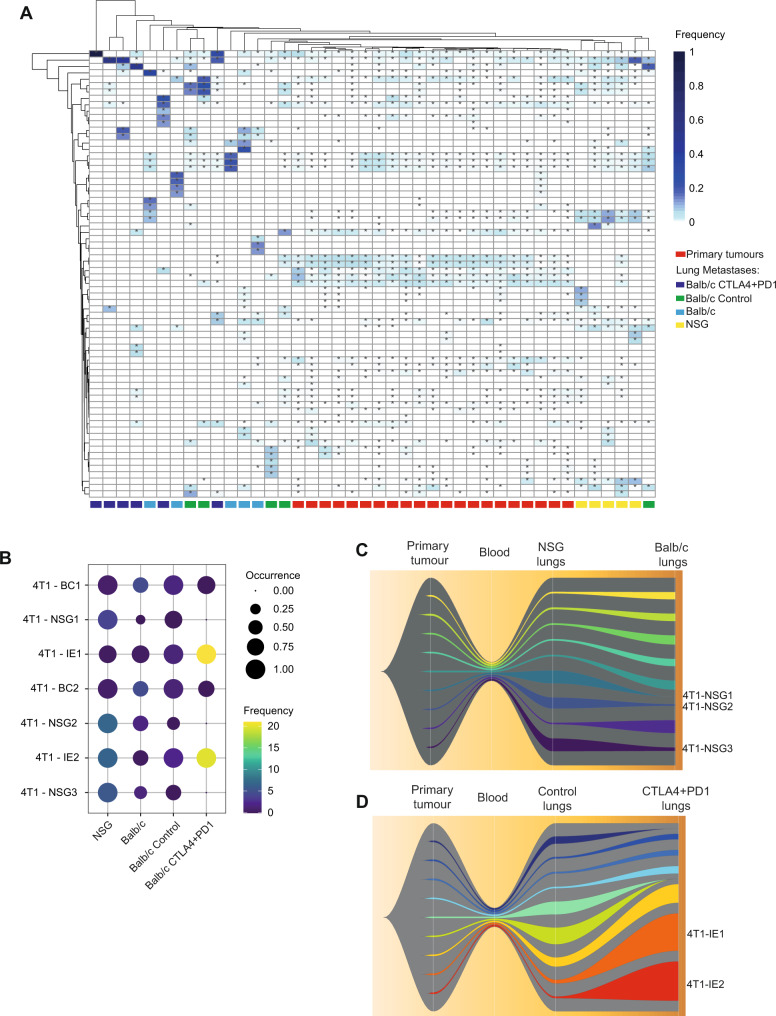
Analysis of specific barcodes enriched or depleted by the immune system and immunotherapy in the 4T1 model. **A** Unsupervised hierarchical clustering heatmap of barcodes with an abundance of above 5% in at least one 4T1 primary tumour or lung metastasis sample; * indicate barcodes detected at a frequency above 0.1% in a particular sample. Each column represents a sample (lung or primary tumour) and each row represents a barcode. **B** Dot plot of a subset of specific barcodes. Five mice per group. Each row represents a barcode. Each column represents a mouse strain or treatment group. **C** Fishplot of the nine most abundant barcodes detected in lungs of WT and NSG mice, each of these nine barcodes is given a unique colour with the remaining barcodes being combined and represented in grey. A bottleneck has been introduced between primary tumour and lung metastases to depict the transition through the bloodstream. **D** Fishplot of the nine most abundant barcodes detected in the lungs of WT mice treated with combination immunotherapy or control antibodies presented as in **C**, a bottleneck has been introduced between primary tumour and lung metastases to depict transition through the bloodstream. Source data are provided as a source data file.

To better understand how specific clonal cell populations responded to the immune system and immunotherapy, we combined the barcode frequencies from the two datasets utilising the 5000 barcode library (WT vs NSG and Control vs Immunotherapy). We performed unsupervised hierarchical clustering of these samples and selected barcodes that were observed at greater than 5% frequency in any one sample. We found that the primary tumours from the two experiments cluster together irrespective of the immune status of the mouse (Balb/c or NSG), further suggesting that 4T1 primary tumours, in contrast to EMT6 tumours, do not undergo further immunoediting (Fig. 4A). In contrast, lung tumours formed in the NSG hosts did not cluster with lung tumours formed in Balb/c mice, with the immunotherapy treated samples mostly clustering alone or with metastases formed in WT mice. A number of specific barcodes were enriched in lung metastases of all NSG mice, indicating that these clones were highly metastatic (Fig. 4A); this is similar to the findings of Wagenblast and colleagues who examined 4T1 clonal diversity in metastases in NSG (but not wild-type mice) and found tissue-specific enrichment of unique barcode clones^32^.

We identified from the heatmap (Fig. 4A) a number of barcodes that had striking patterns of enrichment or depletion in response to the immune system and immunotherapy; we replotted these using a dot plot (Fig. 4B). These barcodes were enriched or depleted in a reproducible manner across replicate mice, suggesting this is due to inherent features of these clones. There are three barcodes that were enriched in NSG lung metastases (NSG1-3), present at lower abundance in untreated wild-type mice, and were completely eliminated following immunotherapy treatment. This suggests that these clones are highly metastatic in the absence of an immune system, but they are immunogenic and are thus subjected to immunoediting in WT mice, particularly following immunotherapy. Another group of metastatic clones present in the lung of NSG and WT mice were further enriched following immunotherapy (IE1-2). These immunotherapy-enriched clones were detected in the lungs of all six replicate mice. With the significant reduction in the number of barcodes present following immunotherapy, the odds ratio of this reproducible enrichment happening by chance is 0.0034 (95% confidence interval: 0.0010–0.0079; chi square *p* value: 3.67 × 10^−251^). This suggests that these clones have a pre-existing resistance phenotype and are positively selected for the following immunotherapy.

To further analyse how specific barcodes were enriched in lung metastases following immunotherapy, we visualised the top nine clonal populations (based on average barcode proportions in the metastatic lungs) and generated fish-plots. These showed that different clones were preferentially enriched in the lung of NSG mice when compared to WT mice (Fig. 4C). Furthermore, we observed that a small subset of clones was highly enriched in the lungs of immunotherapy-treated mice (Fig. 4D).

### Analysis of immunotherapy-resistant clones

To understand more about the phenotype of these immunotherapy-resistant clones, we established clonal cell populations from two of them (designated IE1 and IE2) and from two independent control clones (NT1 and NT2) that were not enriched following immunotherapy. We generated three to four independent clonal cell lines per barcode clone. These clonal cell lines were isolated from the parental barcoded 4T1 cell population in vitro with no additional selective manipulation. The barcode within each of these clonal cell lines was confirmed to be correct using Sanger sequencing. All four clonal cell lines had similar growth kinetics in vitro, indicating no proliferative advantage of the immune evasive clones in vitro (Sup Fig. 9A). All clonal cell lines were able to form tumours in the primary setting (Sup Fig. 9B). IE2 demonstrated considerable metastatic potential with 66% of mice forming extensive lung metastases and regularly metastasised to the lungs (Sup Fig. 9C). IE1 also demonstrated some metastatic capacity with 30% of mice forming lung metastases. Neither NT1 nor NT2 successfully metastasised to the lungs (Sup Fig. 9C).

### Genomic analysis for barcode integration site and copy number variation (CNV)

To identify the barcode integration sites and determine whether the clones contained large-scale genomic alterations, we performed whole genome sequencing (WGS) at around 30x coverage of the clones. The WGS analysis determined the precise genomic location where barcodes were integrated (Sup Table 2). The integration site in IE1 was in the intergenic region between *Kpna*2 and *Smurf*2 and the integration site in IE2 was within an intron of *Nrf*1; neither integration site changed the coding sequence of these genes. Copy number analysis determined that no clone had dramatic copy number changes when compared to other clones. Each clone only contained a small number of single copy number gains and losses (IE1 only 6 CNVs and IE2 only 5 CNVs), with clone NT2 showing the greatest number of CNVs at 41 (summarised in Sup Data 1). We found one locus on chromosome 18 with a single copy number gain in both IE1 and IE2 that led to three copies of *Nc3r1*, encoding the Glucocorticoid receptor, and *Arhgap26*, encoding a Rho GTPase that associates with focal adhesion kinase. However, this copy number gain on chromosome 18 was also present in the NT2 clone. These results demonstrate that large-scale genomic changes unlikely play a major role in determining various phenotypes of different clones but instead suggest that copy number changes may be selected against during immunoediting.

### Transcriptomic analysis of the clonal cell lines

**Fig. 5:**
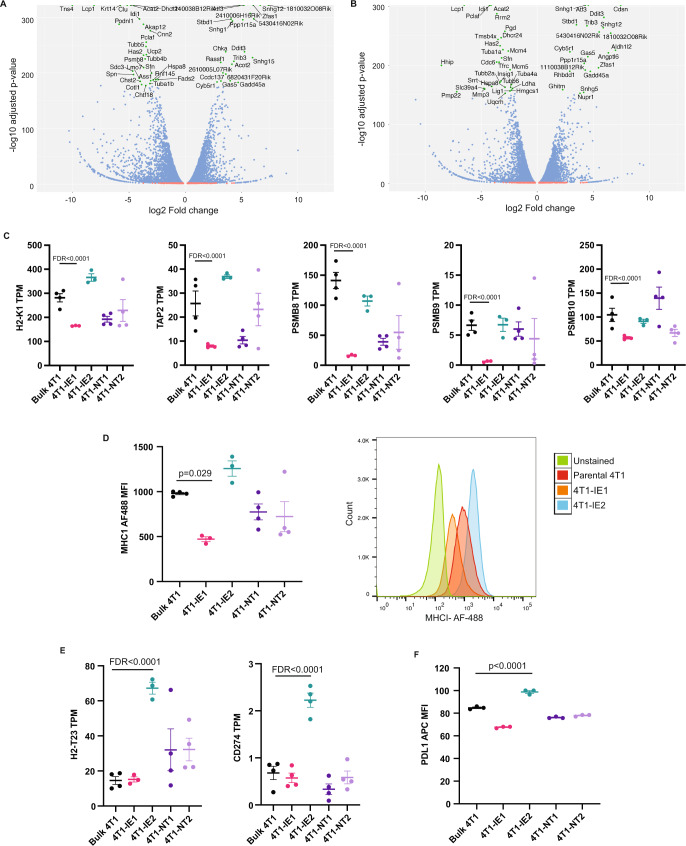
Gene expression analysis of immunotherapy-resistant clones. **A** Volcano plot showing differentially expressed genes between parental 4T1 cells and the immunotherapy-enriched 1 (IE1) clone. **B** Volcano plot showing differentially expressed genes between parental 4T1 cells and the immunotherapy-enriched 2 (IE2) clone. **C** Expression of indicated MHC-related genes, measured as transcripts per million (TPM), in the parental 4T1 population and in indicated cell clones; FDR calculated using EdgeR with Benjamini–Hochberg multiple testing correction. Three independent cell lines in 4T1-IE1 and 4T1-IE2 groups and four independent cell lines in all other groups. Data presented as mean ± SEM. **D** MHC-I protein expression as quantified by flow cytometry in the indicated clones and the parental 4T1 population measured as mean fluorescence intensity (MFI) (left) and representative histogram (right); one-way ANOVA with Tukey HSD for multiple comparison. Three independent cell lines in 4T1 –IE1 and 4T1-IE2 groups and four independent cell lines in all other groups. Data presented as mean ± SEM. **E** Expression of immune-related genes upregulated in clone IE2, measured as transcripts per million (TPM), in the parental 4T1 population and in indicated cell clones. FDR calculated by EdgeR with Benjamini–Hochberg multiple testing correction. Three independent cell lines in 4T1 –IE1 and 4T1-IE2 groups and four independent cell lines in all other groups. Data presented as mean ± SEM. **F**. PD-L1 protein expression as determined by flow cytometry in indicated clones and the parental 4T1 population measured as mean fluorescence intensity (MFI), a representative plot of three independent experiments; one-way ANOVA with Tukey HSD for multiple comparisons. Three independent cell lines in 4T1 –IE1 and 4T1-IE2 groups and four independent cell lines in all other groups. Data presented as mean ± SEM. Source data are provided as a source data file.

To investigate the mechanism of immune evasion by these clones, we performed RNA-sequencing (RNAseq) analysis and compared the two immunotherapy-resistant clones to the bulk 4T1 population. Differential gene expression analysis was carried out using EdgeR. The IE1 clone had 1553 differentially expressed genes (log fold change >2 and FDR *p* < 0.05), with 478 significantly upregulated and 1075 significantly downregulated (Fig. 5A). The IE2 clone had 1099 differentially expressed genes, with 375 significantly upregulated and 724 significantly downregulated (Fig. 5B). The non-target clones had fewer gene expression changes compared to the bulk 4T1 population with NT1 having 621 and NT2 having only 262. We examined the top differentially expressed genes between each of IE1 and IE2 with the parental 4T1 cells, however, we did not find any with an obvious role in immune evasion (Sup Data 2, 3). Gene set enrichment analysis revealed that among the top ten gene sets upregulated in IE1 and in IE2, only two (CHEN_HOXA5_TARGETS_9HR_UP, BLUM_RESPONSE_TO_SALIRASIB_UP) were common between them (Sup Data 4, 5). *Hoxa5* is a known tumour suppressor gene in breast cancer^33^; although we see an enrichment of its target genes, the expression of *Hoxa5* itself was significantly reduced in the IE1 clone and trended to be reduced in the IE2 clone. There was no overlap in the top ten downregulated gene sets between IE1 and IE2. The top downregulated gene set for IE1 was the REACTOME_UB_SPECIFIC_PROCESSING_PROTEASES gene set, which contained two genes involved in antigen processing for display by MHC-I (*Psmb8* and *Psmb9*). As downregulation of the MHC-I pathway is a common mechanism of immune evasion, we investigated this in more detail.

Through this analysis, we found that the IE1 clone had significantly reduced expression of many genes related to antigen presentation, including MHC-I (*H2-k1*), *Tap2*, *Psmb8*, *Psmb9* and *Psmb10* (Fig. 5C). *H2-k1* encodes the main MHC molecule that is expressed by the Balb/c mouse from which the 4T1 carcinoma cell line was derived from. We validated the reduction in MHC-I expression levels seen in the RNAseq data at the protein level using flow cytometry (Fig. 5D). This analysis showed that the IE1 clone had significantly reduced cell surface MHC-I protein compared to the bulk 4T1 population. We thus examined the WGS data and found that the loss of MHC-I expression in IE1 was not due to genomic loss at the MHC locus on chromosome 17 (Sup Fig. 10A). In contrast, the IE2 clone had elevated levels of a number of these MHC-related genes (Fig. 5C) in addition to *H2-t23* that encodes a non-classical MHC molecule (Fig. 5E) known to negatively regulate NK cells through their inhibitory receptor NKG2A^34^. Interestingly, IE2 cells also demonstrated a significantly increased expression of *Cd274* that encodes the T cell inhibitory molecule PD-L1 (Fig. 5E). This again was validated at the protein level using flow cytometry (Fig. 5F). These results demonstrate that these two immunotherapy-resistant clones are phenotypically unique. To determine if these two mechanisms are simultaneously maintained in vivo, we carried out flow cytometry for MHC-I and PD-L1 on advanced 4T1 lung metastases from immunotherapy-treated BALB/c mice. By isolating RFP + cancer epithelial cells from lung tissue, we observed MHC-I-high and MHC-I-low neoplastic populations, both with varying expression of PD-L1 (Sup Fig. 11). This provides convincing evidence of these two mechanisms of immune evasion being maintained simultaneously in vivo.

We next examined whether copy number changes or barcode integration sites identified above-impacted gene expression. The copy number changes of *Nc3r1* and *Arhgap26* were associated with a significant increase of their expression in IE1 and IE2 cells but not in NT2 cells (Sup Fig. 10B). Elevated *NC3R1* expression has been associated with poor prognosis and metastasis in triple-negative breast cancer, although whether it plays a role in immune evasion is not known^29,35^. As stated above, the barcode integrated into the intergenic region between *Kpna2* and *Smurf2* in IE1 and within an intron of *Nrf1* in IE2. Among these three genes, only the expression of *Smurf2* was significantly altered, with a modest log fold increase of 0.59 in IE1.

### Demethylating drugs do not fully restore MHC expression

Demethylating agents such as 5-aza-2′-deoxycytidine (5-aza) are known to upregulate MHC-I expression in cancer cells^36^, thus we treated our clonal cell lines with 5-aza for 72 h to determine whether DNA methylation was a mechanism suppressing MHC expression in the IE1 clone. Using flow cytometry, we observed that MHC-I expression was elevated in a dose-dependent manner following 5-aza treatment in all clones. However, MHC-I expression in the IE1 clone was consistently lower than the parental 4T1 cell line at all doses of 5-aza (Sup Fig. 12A). This indicates that gene hyper-methylation is not the mechanism of MHC-I suppression in the IE1 clone.

Interferon-gamma (IFN-gamma) stimulation is another mechanism by which MHC expression can be increased in cancer cells. The IE1 clone responded to IFN-gamma treatment by upregulating MHC-I expression, but again it remained suppressed compared to the parental 4T1 cells (Sup Fig. 12B). This suggests these cells broadly retain the transcriptional regulatory machinery that is required to upregulate MHC-I in response to IFN-gamma stimulation. These results indicate that MHC-I downregulation is likely regulated by epigenetic factors other than DNA methylation and that the majority of MHC-I expression in this clone can be restored by IFN-gamma treatment.

### The 4T1-IE2 clone can directly suppress anti-cancer CD8 T cell responses

**Fig. 6:**
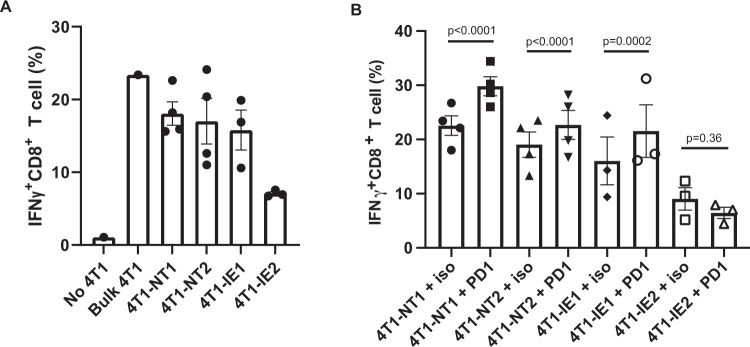
The 4T1-IE2 clone can directly suppress cytotoxic T cell activation and prevent further T cell activation by anti-PD1 immunotherapy. **A** Measurement of intracellular IFN-gamma (IGNγ) production by 4T1-specific CD8 + T cells following co-culture with no 4T1 cells (No 4T1), bulk 4T1 cells (Bulk 4T1) and 4T1 clonal populations (4T1-NT1; 4T1-NT2; 4T1-IE1; 4T1-IE2). Four independent biological replicates in NT1 and NT2 groups, three independent biological replicates in IE1 and IE2 groups and one replicate for no 4T1 and bulk 4T1 groups. Data are shown as mean ± SEM. **B** Measurement of intracellular IFN-gamma production by 4T1-specific CD8 + T cells following co-culture with clonal 4T1 populations with or without anti-PD1 (PD1) or an isotype control (iso). Three biological replicates in IE1 and IE2 groups and four biological replicates in NT1 and NT2 groups. *P* value calculated using flow cytometry proportions fitted to a GLM with Bonferroni post hoc correction for multiple comparisons. Data are shown as mean ± SEM. Source data are provided as a source data file.

To further characterise the immunotherapy-resistant clones (IE1 and IE2) in comparison to the control (NT1 and NT2) clones, we performed an in vitro CD8 T cell activation assay^37,38^. This assay utilises a pool of de novo-generated 4T1-specific CD8 + T cells, with intracellular IFN-gamma production utilised as a functional measure of T cell activation. We then determined how T cell activation changed following co-culture with the clonal 4T1 populations. In the absence of 4T1 cells, 1% of CD8 T cells had detectable intracellular IFN-gamma production (Fig. 6A), while in the presence of the parental 4T1 cells, 23% of the T cells had detectable intracellular IFN-gamma production. When the activation of T cells by clonal cell lines was examined, a decrease in CD8 T cell activation was seen only in the IE2 clones. As we had demonstrated that the immune evasive clones were also more immunotherapy resistant, we examined the effect of adding anti-PD1 immunotherapy to the T cells in vitro. The addition of anti-PD1 significantly increased anti-cancer T cell responses to the control clones and the IE1 clones, while T cell response to the IE2 clone was unchanged with the addition of anti-PD1 (*p* = 0.36; Fig. 6B).

This assay demonstrated that the IE2 clone uniquely evades activation of anti-cancer CD8 T cells and that the addition of anti-PD1 could not overcome the T cell suppression in response to this 4T1 clone. In contrast, the IE1 clone induced a similar response to the control clones suggesting that it does not directly suppress CD8 T cell activation.

### Overlapping gene signature is associated with poor survival in breast cancer patients

**Fig. 7:**
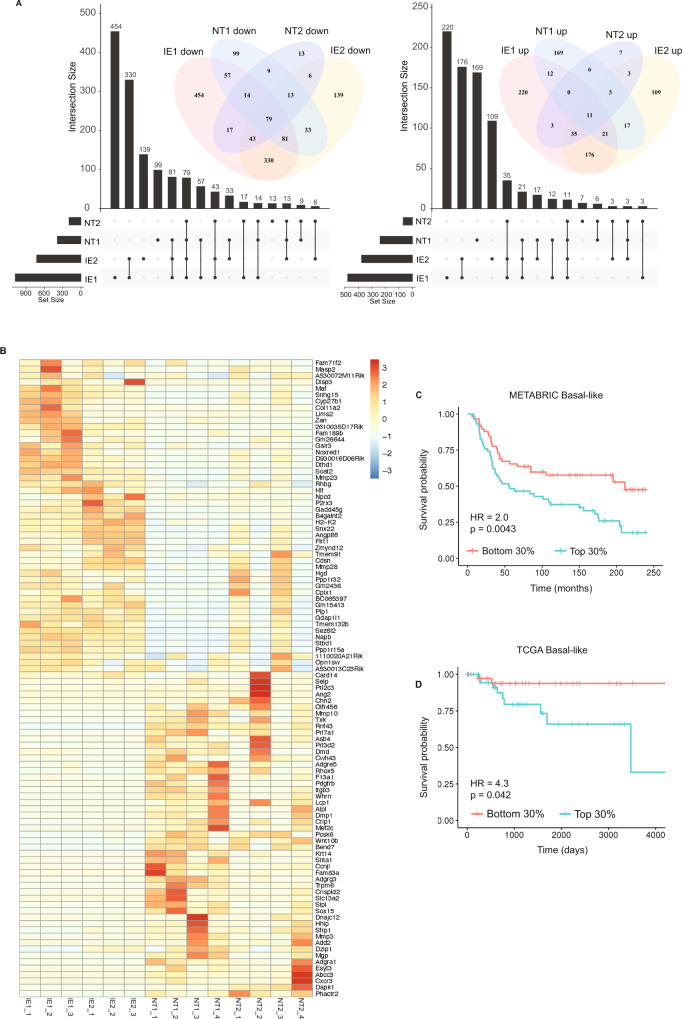
Overlapping gene signatures of the immunotherapy-resistant clones show prognostic significance in basal-like breast cancer patients. **A** Upset plots and Venn diagrams show the overlap in significantly upregulated (right) and downregulated (left) genes between the two immunotherapy-enriched clones (IE1 and IE2) and two control clones (NT1 and NT2). **B** Heatmap of the top 50 overlapping upregulated and downregulated genes between IE1 and IE2 clones across all clonal cell lines. **C** Kaplan–Meier analysis of overall survival of basal-like breast cancer patients in the METABRIC cohort based on whose breast cancer having the top 30% (*n* = 58) or the bottom 30% (*n* = 58) expression of the overlapping upregulated 25 gene signature. 116 patients total, with 58 per group. **D**. Kaplan–Meier analysis of overall survival of basal-like breast cancer patients in the TCGA cohort based on whose tumour having the top 30% (*n* = 40) or the bottom 30% (*n* = 40) expression of the overlapping upregulated 25 gene signature. Eighty patients total, with 40 per group. In **C**, **D**, the Cox proportional hazards model was used to compute hazard ratios. Significance between stratification groups were computed using log-rank test statistics.

We noted that the GSEA analysis showed some overlap in enriched gene sets between IE1 and IE2. We thus reasoned that, in addition to having their unique immune evasion features, these clones may have some pathways in common. To identify immune evasion pathways common to both immunotherapy-resistant clones, we identified overlapping gene expression changes (Sup Data 6). This analysis demonstrated that immunotherapy-resistant clones had more genes with expression changes in common than either one had with either non-target clones (Fig. 7A). We generated a heatmap of the top 50 upregulated and downregulated genes across all samples (Fig. 7B) and performed GSEA analysis using C2 on the longer list (Sup Fig. 13A and Sup Data 7). Only two gene sets had significant p values when multiple testing was considered, these were the *Hoxa5* gene set mentioned previously and a COVID-19-related gene set. Although not significant, there were several additional COVID-19-related gene sets from the same recent publication identified in the overlapping upregulated gene list, suggesting an immune-related role of these genes^39^. We also identified three gene sets that were closely related and had clear associations with epigenetic regulation, BENPORATH_ES_WITH_H2K27ME3, BENPORATH_PRC2_TARGETS and BENPORATH_SUZ_12_TARGETS. In patient data, Ben-Porath and colleagues found negative enrichment of these gene sets to be correlated with a stem-like phenotype and to be associated with poor prognosis^40^. Further investigation showed the top genes driving these signatures were also negatively enriched in our derived gene signature, namely *Hhip*, *Cwh43*, *Wnt10b*, *AbcA3*, *Chn2* and *Crip1*. This suggests a possible role for PRC2-mediated MHC-I suppression in our subclones.

To understand the role of these genes in patients, we generated signatures from the top 25 upregulated and downregulated genes that had human orthologs and were detectable in both the METABRIC^41^ and TCGA^42^datasets. We then analysed the association of these signatures with the survival of patients with basal-like breast cancer from these cohorts. We limited our analysis to basal-like breast cancer as we considered this the most relevant patient subgroup as the gene signature was derived from the 4T1 mouse model of the basal-like breast. Although patients in these cohorts were not treated with immunotherapy, it has previously been demonstrated that immune features such as the number of tumour infiltrating lymphocytes or regulatory T cells influence prognosis in basal-like breast cancer patients^43^. When we analysed overall survival, we observed that the upregulated gene signature was associated with significantly poorer outcomes in both cohorts (METABRIC: *p* = 0.0043, HR = 2.0, Fig. 7C; TCGA: *p* = 0.042, HR = 4.3, Fig. 7D). We generated heatmaps with unsupervised clustering to determine whether specific individual genes or groups of genes from the signature were driving the association with survival (Sup Fig. 13B). In the TCGA dataset, we observed a number of clusters that seemed to associate more with survival, including *FAM71F2, MASP2, HLF, PPP1R15A, MMP23B* and *LIMS2*. Interestingly, GADD34, encoded by *PPP1R15A*, had previously been demonstrated to be critical in blocking immunogenic cell death following chemotherapy, when it was inhibited, the chemotherapy response was improved in immunocompetent mice but not in immunocompromised mice^44^. The second group of genes included *SEZ6L2*, which had been associated with survival in a number of cancers but not through an immune-related mechanism^45,46^ There was no enrichment of proliferation or invasion gene sets in our GSEA analysis, suggesting that these processes were not behind the poor outcome of patients whose breast cancer highly expressed genes in the common upregulated signature.

Previous studies have shown that cytotoxic T lymphocytes (CTL) infiltration correlates with survival in basal-like breast cancer, so it is possible that our signature was a surrogate measure of T cell infiltration. To test this, we performed TIDE analysis^47^ on TCGA and METABRIC cohorts, followed by correlation analysis between the CTL signature score and our upregulated immune evasion signature score. This showed no correlation in the METABRIC cohort (Sup Fig. 13C) and only a weak negative correlation in the TCGA cohort (Sup Fig. 13D), suggesting little overlap between these two predictors of patient survival. Thus, it suggests we have discovered an immune-related signature associated with patient survival in basal-like breast cancer. Future studies will be necessary to determine how the genes in this signature regulate survival, influence immune evasion and immunotherapy response.

## Discussion

Immunotherapy has revolutionised cancer therapy, with long-term responses seen in certain patient groups with a few types of cancer. Unfortunately, some patients with these same types of cancer and most patients having other types of malignancies, including breast cancer, have limited to no response to the current immunotherapies. Thus, our understanding of immune evasion in non-responsive cancer types needs significant improvement. We have addressed this by examining immune evasion at a clonal level and used this information to identify pathways that could be targeted to overcome immunotherapy resistance.

Here we show that clonal immunoediting occurs and is enhanced by immunotherapy. Using the more immunotherapy-sensitive EMT6 model, we demonstrate that immunoediting occurs during the development of primary tumours and that immunotherapy leads to strong clonal selection. Most of the more immunotherapy-resistant 4T1 cells are able to evade the immune system during the development of primary tumours, but only a subset of them are able to evade the immune system during metastasis. This indicates that immune evasion is not a static process but requires ongoing regulation through tumour progression, even in a highly aggressive allograft model. These findings broadly agree with findings from a recent comprehensive genomic analysis of patient samples assessed across metastatic sites and over time^3,48^. These studies tracked clonal populations in metastatic lesions using whole genome sequencing, examined a number of immune correlates and identified immunoediting that was associated with the immune response. However, they were unable to examine clonal heterogeneity driven by epigenetic or transcriptomic changes and were limited in the identification of rare clones by sequencing depth. Future clinical studies utilising single-cell approaches to analyse multiple biopsies from individual patients over a time course of treatment will be necessary to confirm the key findings of this study in patients. While challenging, these studies are becoming more feasible with recent technological improvements.

Intriguingly the 4T1 model, unlike the EMT6 model, showed little immunoediting in the primary tumour. This suggests that either the vast majority of 4T1 cells are inherently resistant to immune control at the orthotopic site, or that 4T1 cells very rapidly set up a suppressive immune microenvironment that protects the majority of clones from immune-mediated killing. The ability of 4T1 cells to induce myeloid-derived suppressor cells could well contribute to a suppressive immune microenvironment, however, further studies would be necessary to further clarify this^49^.

As we wanted to understand the role of immunotherapy in controlling metastatic disease, we examined lung metastases from the 4T1 model following the resection of the primary tumour. Lung metastasis occurs early in the 4T1 model, with micrometastases forming by day 14^50^ and others showing the related 4T1.2 model robustly metastasises by day 10^51^. This indicates that when adjuvant immunotherapy was given, these therapies were activating immune cells to target micrometastases that had already formed within the lungs. We postulate that while possible, it is unlikely that circulating cancer cells were a major target of adjuvant immunotherapy as previous studies have indicated that circulating breast cancer cells only have a short half-life of 1–2.4 h in circulation^52^.

A number of previous barcoding studies in breast cancer had focused on metastasis and response to chemotherapy. However, these were performed in immunocompromised mice, so the role of the immune system in these processes was not addressed^32,53–55^. In the absence of a fully intact immune system, it was demonstrated that specific clones have greater metastatic ability^32,53,55^. Our results suggest that in the context of an intact immune system, a subset of these highly metastatic clones identified in these studies may have been recognised and removed via immunoediting. Similar to our results, these studies found that the dominant clone within the primary tumour generally was not the dominant clone in the metastases^32,53,55^. Some of these studies also showed that chemotherapy treatment of PDX models led to a decrease in clonal abundance and diversity in relapsed disease, which is similar to what we found with immunotherapy^54,55^. Future studies combining immunotherapy with chemotherapy utilising a similar regimen to the atezolizumab plus nab-paclitaxel of the Impassion130 trial would give important insights into how combining these two treatment modalities affected clonal diversity following relapse^56^.

Our and others’ studies have indicated that immunoediting can select clones with immune evasive phenotypes irrespective of specific neo-antigens^2,6,57^. One previous study examining immunoediting at a clonal level used a fluorescent barcoding approach in a B cell leukaemia model^13^. While our findings broadly agree with the findings of that study, the study by Milo and colleagues was limited to five unique fluorescent clones that could be tracked and was confounded by the variable immunogenicity of these fluorescent proteins. DNA barcoding, in contrast, allowed for the labelling of thousands of clones and much more precise identification of immune evasive clones. We could then isolate these clones and identify both common and variable features of immune evasion in them. Furthermore, this technique, unlike a fluorescent barcode approach, allowed us to demonstrate that clones that had greater immunotherapy resistance pre-existed in both EMT6 and 4T1 models, as these clones were enriched from the same starting pool of cells across replicate mice.

We identified and isolated two immunotherapy-resistant clones from the 4T1 model. An in-depth analysis of these resistant clones demonstrated that they expressed genes of key immune evasive pathways (MHC-I and PD-L1) differently. Intratumoural heterogeneity (ITH) has previously been associated with resistance to immunotherapy in melanoma and lung cancer, with higher ITH being associated with resistance to immunotherapy^26,27,58^. McGranahan and colleagues postulated that this was due to improved T cell killing of tumours with clonal neo-antigens. A non-mutually exclusive explanation is that clonal tumours are less likely to contain cancer cells with a pre-existing resistance mechanism to immunotherapy. These findings refine the concept of cancer immunoediting, demonstrating that there are clonal populations of cancer cells with variable resistance to the immune system. Based on their phenotype, these clones are either enriched or depleted by an active immune system and immunotherapy.

We identified a core overlapping gene expression profile between the two immunotherapy-resistant clones. The common upregulated gene signature was able to stratify basal-like breast cancer patients into good and poor prognosis. This gene signature appeared to represent an immune evasion pathway associated with poor prognosis. Aside from *Ppp1r15a*, this signature did not contain genes known to be associated with immune evasion, and it did not contain genes associated with other poor prognostic signatures, such as proliferation or invasion.

Because both T cells and NK cells are present during immunoediting in our models, this common signature likely enables cancer cells to evade both T cells and NK cells. Interestingly, our common signature does not strongly correlate with CTL infiltration, which indicates that this signature is not a surrogate for the lack of T cell infiltration and suggests that these genes likely do not regulate immune evasion by influencing immune cell recruitment. These common genes may offer insights into developing therapeutic approaches to improve immunotherapy response in breast cancer. One of the common immunotherapy resistance genes we identified was *PPP1R15A*, which is consistent with its known role in immunogenic cell death in response to chemotherapy^44^. Recent studies in a mouse model of multiple sclerosis demonstrated that this pathway can be targeted using Sephin-1, a small molecule^59^. Future studies are necessary to examine whether this compound or others targeting this pathway could synergise with immunotherapy or immunogenic chemotherapy to treat breast or other types of cancer. As breast cancers have a relatively low mutational burden, it is likely that epigenetic factors may play a greater role than mutational events in driving ITH in breast cancer. One current approach to improve immunotherapy response under investigation is combining immunotherapy with epigenetic targeting drugs such as decitabine and HDAC inhibitors^36,60^. This combination has been shown to increase MHC protein expression and improve response to immunotherapy. However, epigenetic drugs may reduce the diversity of clones and overcome other epigenetically driven immune evasion mechanisms in addition to enhancing MHC expression^61^. Further research is needed to test this hypothesis more fully. Our results, however, suggest that while demethylating agents could increase MHC-I expression in the MHC-I low immunotherapy-resistant clone, they did not increase it above the baseline seen in the parental 4T1 cell line. This is corroborated by a recent study demonstrating that while treating breast cancer patients with demethylating agents could increase MHC-I expression in most patients’ tumours, a subset appeared resistant to this therapy^36^. This suggests that while epigenetic treatments may improve the proportion of patients responding to immunotherapy, in some cases, pre-existing clones could still mediate resistance to this combination.

A limitation of this study is the reliance on mouse cell line models, which do not recapitulate the early stages of tumorigenesis and do not represent the full diversity of human breast cancer. However, syngeneic allograft models have delivered central insights into the immune response to cancer and demonstrated the utility of immunotherapies^62^. Another limitation is that the integration of the barcode and selection markers into the genome and the potential immunogenicity of red fluorescent protein (RFP) could affect the phenotype of these cancer cells. We and others have found in previous studies that some fluorophores and luciferase were immunogenic and negatively affected tumour growth and metastasis in the 4T1 model^35,63^. However, we found that tumour growth and metastasis were unaffected by RFP expression in this study. While the introduction of DNA barcodes could have influenced the phenotype of specific clones, we feel that this is unlikely, given that no dramatic impact on the expression of the genes closest to the integration site. Furthermore, none of the genes associated with integration sites was identified to be significantly involved in cancer cell evasion of CD8 T cell responses in a recent CRISPR screen^16^.

To survive in any given system, cancer cells must utilise a number of mechanisms to avoid more than just immune destruction, as extensively reviewed in the recent work of Hanahan^64^. Not only are IE1 and IE2 immune evasive, they are also highly metastatic and, by their very nature, must be able to grow independent of anchorage, as well as possessing abilities to engraft in both the mammary fat pad and lungs, shed from the primary tumour prior to resection and resist all other mechanisms of host anti-cancer response. We attribute the decrease in unique clones present in the lungs of control-treated 4T1 tumour bearing mice compared to the primary tumour to precisely this; only some clones within the engrafted tumour possess the required abilities to be able to successfully metastasise. By making comparisons between controlled conditions, e.g. immunotherapy-treated lungs vs control-treated lungs, we believe we have effectively demonstrated immunoediting occurring in vivo.

Overall, this study has demonstrated that immunoediting occurs at the clonal level in primary tumours and that the second round of immunoediting occurs during metastasis. Immunotherapies dramatically enhanced immunoediting, but pre-existing resistant populations were still responsible for relapse. The large reduction in clonal diversity following immunotherapy in the 4T1 model, which is known to be poorly responsive to immunotherapy, suggests that slight improvements through combination therapy could eliminate the remaining clones and lead to dramatic improvements in survival. By isolating immunotherapy-resistant clones and characterising them, we identified common and distinct immune evasion pathways. We anticipate that through targeting pathways identified in this study, in particular common pathways, it will be possible to further reduce the number of resistant clones and improve the efficacy of immunotherapies.

## Methods

### Cells

4T1 cells were obtained from ATCC (CRL-2539). 4T1 cells were grown in RPMI (Gibco) supplemented with 10% FCS (HyClone), d-Glucose, sodium pyruvate, 2 mM HEPES and Penicillin/Streptomycin. EMT6 cells were obtained from ATCC (CRL-2755). EMT6 cells were grown in Waymouth’s MB 752/1 Medium supplemented with 15% FCS (HyClone) and 2 mM l-glutamine. Cells were tested for mycoplasma contamination using the MycoAlert Mycoplasma Detection Kit, 100 Test Kit (Catalogue# LT07-318). No further cell line authentication was performed, but ATCC provides cell line authentication prior to dispatch.

### Cellular DNA barcoding

The ClonTracer library was a gift from Dr. Frank Stegmeier (Addgene #67267). Lentiviral particles containing the high-complexity barcode library were produced by transfecting 293 T cells. 4T1 and EMT6 cancer cell lines were barcoded by lentiviral infection using 0.8 µg/ml polybrene. Cells from each line were infected with a target MOI of 0.1, corresponding to 10% infectivity to ensure single lentiviral integration. Cells that received a barcode were then sorted based on the RFP reporter protein using a BD FACSAria. These cells were then expanded and frozen into a number of aliquots for the subsequent experiments. 4T1 cells were generated with two different barcode complexities, one with ~5000 barcodes (4T1 BC5000) and one with ~300,000 barcodes. The EMT6 cells and the high-complexity 4T1 cells were passaged twice following cell sorting, frozen and cells from these aliquots were thawed and passaged once more prior to transplantation. The low complexity 4T1 cells (4T1 BC5000) were a subpool derived from the higher complexity 4T1 cell line; these were passaged an additional three times to expand and freeze and were then thawed and used as described above.

### In vivo experiments

All animal experiments were approved by the Garvan Institute of Medical Research/St. Vincent’s Hospital Animal Experimentation Ethics Committee, approval 19/04.

Immunocompetent BALB/c female mice (BALB/cJAusB) and immunocompromised NOD.Cg-*Prkdc*^*scid*^ *Il2rg*^*tm1Wjl*^/SzJ (NSG) female mice aged 6-to-8 weeks were obtained from Australian BioResources (Moss Vale, Australia) and housed at the Garvan Institute of Medical Research. Mice are housed at 21 °C (±1 °C) with 50–60% humidity, a light/dark cycle of 12 h, and chow and water available ad libitum. Mouse numbers are stated in all figure legends. Ethical endpoint definition included tumour burden exceeding 1500 mm^3^, continued weight loss or respiratory distress. Mice were humanely euthanised if any of these ethical endpoints were reached. All experiments were carried out in accordance with the above-described conditions.

### In vivo tumour growth

For tumour transplantation, barcoded EMT6 cells (ATCC, USA) were resuspended in Matrigel 2.5 × 10^5^ cells in 100 ml volume were injected into the fourth inguinal mammary gland. Barcoded 4T1 cells (ATCC, USA) were resuspended in PBS and 5 × 10^4^ cells in a 10 ml volume were injected into the fourth inguinal mammary fat pad. For studies with the 4T1 model primary tumours were surgically resected at day 15. At resection or ethical endpoint tumours and whole lung tissue were removed, minced and snap-frozen in liquid nitrogen for barcode analysis.

### Immunotherapy treatment

Mice were treated with four 200 μg doses of either combination immunotherapy antibodies via intraperitoneal injection: anti-CTLA4 (clone UC10-4F10-11, cat# BE0032), anti-PD1 (clone RMP1-14, cat#BE0146), or isotype control antibodies Armenian hamster IgG (clone 2A3, cat#BE0091), Rat IgG (clone 2A3, cat#BE0089) all from BioXCell (Lebanon, NH, USA). No antibody validation was carried out after purchase. Antibodies were given every 2–3 days from day 10 after tumour implantation for the EMT6 model and following resection of the primary tumour on day 15 for the 4T1 model.

### CD8 T cell, CD4 T cell and NK cell depletion

Starting one day prior to primary tumour resection mice were received IP injections of 100 μg of depleting antibodies for CD8 T cells (anti-CD8; clone53-5.8, cat#BEO223; BioXCell), or NK cells (anti-Asialo-GM1; 986-10001; Novachem), or CD4 T cells (anti-CD4; clone: GK1.5, cat#BE0003-1; BioXCell) or isotype control antibodies. Antibodies were then given every 2–3 days for a total of four doses. No antibody validation was carried out after purchase.

### Isolation of buffy coat and flow cytometry to confirm depletion of key cell types

Whole blood was collected into K2EDTA coated tubes (BD, cat# 365973) from mice by tail nick 2 days after the last intraperitoneal injection of depleting or control antibodies. The Buffy coat was isolated by spinning blood at 800×*g* at room temperature for 10 min and removing the small layer of the buffy coat. Any carryover red blood cells were lysed using Pharmlyse (BD, 5 min) and quenching with FACS buffer (DPBS supplemented with 2% FCS and 2% HEPES). Cells were washed and stained with BV711 conjugated anti-mouse CD8a (clone53-6.7, Biolegend, 1:200, cat# 563046), PE-conjugated anti-mouse CD4 (clone RM4-5, Biolegend, 1:200, cat# 116006), and APC conjugated anti-mouse NKp46 (clone 29A1.4, Biolegend, 1:200, cat# 137607). Cells were washed three times before staining with DAPI. Data were collected using the BD LSR Fortessa (FACSDiva 8.0.1).

### DNA extraction

Frozen tissue samples were lysed in 5 ml QIAGEN buffer P1 (with RNaseA) and 0.5% SDS within a Miltenyi M-Tube (# 130-096-335). Samples were processed on the gentleMACS or gentleMACS Octo using the RNA_02 programme. DNA was then extracted using a standard phenol/chloroform process.

### Targeted barcode PCR and sequencing

All samples underwent targeted barcode PCR amplification according to the updated version of the original protocol^29^ available on the Addgene website (https://www.addgene.org/pooled-library/clontracer/). Specific PCR products (180 bp) were gel purified, quantified by Qubit 2.0 fluorometer (Thermo Fisher Scientific, Waltham, MA, USA) and pooled into a library. Prior to sequencing, an equal combination of additional PCR products containing two inverse barcodes (GACTCAGTGTCAGACTGAGTGTCTGACTGT and CTGAGTCACAGTCTGACTCACAGACTGACA) plus the PhiX Control V3 (Cat. FC-110-3001, Illumina, CA, USA) were spiked in to balance the nucleotide distribution within the library. Samples were sequenced using a custom sequencing primer (GCGACCACCGAGATCTACACACTGACTGCAGTCTGAGTCTGACAG) and the NextSeq® 500/550 Mid Output Kit v2—150 cycles (FC-404-2001, Illumina, CA, USA) on the Illumina NextSeq® platform, with Basespace v5.31 for all main figures, and v6.2 for Supplementary Fig. 6F.

### Barcode analysis

Barcode composition analysis and calculation of barcode overlap between samples was performed as indicated in the original protocol^29^ and updated Python scripts available from the Addgene website (https://www.addgene.org/pooled-library/clontracer/).

Further analysis was performed using R (v3.6.1 and v4.0.2) statistical framework and packages EntropyExplorer (v1.1) for analysis of differential Shannon Entropy^65^, DEBRA for differential barcode expression^66^, and libraries fishplot (v0.5.1) and UpSetR (v1.4.0), and RcolorBrewer (v1.1.2) for visualisation purposes. Pheatmap (v1.0.12) was used with default parameters. Clustering distances for rows and columns were euclidean with complete clustering linkage.

### Generating clonal cell lines

Cells of interest were isolated from the barcoded parental population using a sub-pooling approach.

The barcoded 4T1 BC5000 cells were seeded into a 96-well plate at a density of 150 cells per well. At ~80% confluence, cells were trypsinised and split identically into two plates. One plate was viably frozen in freezing media (10% DMSO, 40% FCS and 50% 4T1 media). DNA was extracted from one plate using the Promega SV Wizard Genomic DNA kit. Target barcodes of each sample were PCR amplified and sequenced using the method described above.

After sequencing, wells containing cells with the target barcodes were thawed, pooled and seeded at 40 cells/well in a 96-well plate. Media was changed every 3 days for 8 days before cells were split into two identical plates as above. One plate was viably frozen in freezing media, while DNA was extracted and prepared for targeted sequencing as above.

Wells with the highest proportion of target barcodes were revived into a 6-well plate and grown for 4 days before being single-cell sorted by BD FACSAria II (with FACSDIVA 8.0.1) into a 96-well plate. Sorted single cells were grown in conditioned media for 5 days before being changed to 4T1 media and grown until 80% confluence. Cells were lifted and split identically into two plates—one for freezing and one for targeted sequencing.

Wells containing single cells clones of the cells of interest were identified and target wells were revived. Cells were expanded before being aliquoted and viably frozen for future experiments.

Barcoded sequences of isolated cells were confirmed by targeted Sanger sequencing of barcode regions.

### Bulk RNA sequencing

RNA was extracted from established subclonal cell lines using the QIAGEN RNeasy Mini Kit. Three to four unique clonal cell populations were sequenced for each barcode. Libraries were prepared using the KAPA RNA HyperPrep Kit with RiboErase, and sequenced on the NextSeq500 platform using a High Output V2.5 300-cycle kit.

### Transcriptome analysis

FastQ files from sequencing libraries were first trimmed with FASTQC v0.11 Andrews S. (2010)*.* FastQC: a quality control tool for high throughput sequence data. Available online at: http://www.bioinformatics.babraham.ac.uk/projects/fastqc. Raw reads were subsequently mapped to the mouse transcriptome (Gencode release M9, GRCm38.p4, https://www.ncbi.nlm.nih.gov/assembly/GCF_000001635.24/), to the mouse genome (mm10 assembly), with STAR aligner v.2.4.1d, allowing for multimapping reads^67^. The reads were counted over gene models with RSEM, v.1.2.18^51^. Differentially expressed genes and repeat elements were defined with EdgeR (v3.3.8) with FDR <0.01^68^. EdgeR uses genewise negative binomial generalised linear models through the function glmQLFTest^69^. Genes with less than ten reads across three samples per group were omitted from the analysis. Two bulk 4T1 and two NT2 samples were prepared on separate days from the remainder of the samples, introducing a batch effect into the data. This was corrected by fitting a matrix model using the model.matrix() function in R.

### Survival analysis

To assess the clinical relevance of our isolated immune evasion clones, we assessed the association between the gene signatures derived from our bulk RNA-Sequencing studies with the overall survival of basal (PAM50) breast cancer patients from the METABRIC and The Cancer Genome Atlas (TCGA; https://www.cancer.gov/tcga) cohorts. Mouse gene signatures were first converted to human orthologs using the biomaRt package v2.5^70^. Shared upregulated genes across both immune evasion clones IE1 and IE2 were then filtered, and only genes detected in each expression cohort were considered. For each tumour from the bulk cohort, signature scores were computed based on the average expression of the top 25 genes ranked by log fold change. Patients were then stratified based on the signatures scores into the top 30%, middle 40% and bottom 30% groups. Survival curves were generated using the Kaplan–Meier method with the ‘survival’ package in R (https://cran.r-project.org/package=survival, v3.2.7). The Cox proportional hazards model was used to compute hazard ratios. We assessed the significance between groups using the log-rank test statistics.

### Gene set enrichment analysis

Gene set enrichment analysis (GSEA) was carried out using the GSEA desktop app (4.1.0) and DEGs generated with EdgeR. GSEA was run using preranked list of significantly differentially expressed genes, ranked by log fold change. The molecular signatures database (MsigDB v7.5.1) hallmark and curated (C2) gene sets were used for analysis.

### Whole genome sequencing

DNA was extracted from established subclonal cell lines using the QIAGEN DNeasy blood and tissue kit. Libraries were prepared using the Roche KAPA PCR-free library preparation kit and genomes were sequenced on the HiSeq X platform to a depth of ~30x.

### Whole genome analysis

Fastq files from the WGS were firstly aligned to mouse genome reference mm10. The output bam files were subsequently used for copy number analysis. Copy number analysis was performed using an R package cn.mops (version 1.4.2)^71^ in paired mode with a window length of 10 kb. Reads were aligned to the BALB/c reference genome using BWA (v0.7.8) before being indexed and sorted with Novosort (v1.03.8). Reads that mapped incompletely to the reference genome were then mapped to the barcode plasmid sequence with BWA and sorted and indexed with Novosort. Read pairs, where only one pair mapped to the barcode plasmid sequence, were blasted (NCBI BLAST v2.9) against mm10 to establish the barcode plasmid insertion site.

### Flow cytometry for MHC-1 and PD-L1

The 4T1 subclones (IE1, IE2, NT1 and NT2), as well as the parental 4T1 bulk population, were revived and passaged three times before being seeded into a 6-well plate at a density of 200,000 cells per well. At ~80% confluence, cells were collected into FACS buffer (DPBS supplemented with 2% FCS and 2% HEPEs) for flow cytometry. Cells were stained with a mastermix of APC conjugated anti-mouse CD274 (Biolegend, clone 10 F.9G2, cat#124311, 1:200) and Alexa Fluor488 conjugated anti-mouse H2-kD (Biolegend, clone SF1-1.1, cat#116610, 1:200) in FACS buffer for 20 min. Cells were washed three times with FACs buffer before being stained with DAPI and run on the BD FACSCanto II flow cytometer, utilising BD FACSDIVA software (v8.0.1). Data were analysed in FlowJo (version 10.6.1) and the median fluorescence intensity of live, single cells was calculated.

### Treating cells with 5-Aza-2′-deoxycytidine and flow cytometry for MHC-I

5-Aza-2′-deoxycytidine (5-aza) was sourced from Sigma (cat#A3656) and reconstituted in DMSO according to the manufacturer’s instructions. Subclones (IE1 and IE2) and the parental 4T1 cell line were seeded into a 24-well plate at a density of 8000 cells per well in 4T1 media. Cells were allowed to settle overnight before being treated with 5-aza at 200, 100 or 50 nM for 72 h. 5-aza was removed and cells were cultured in media only for 24 h before being collected for flow analysis. Cells were stained with Alexa Fluor488 conjugated anti-mouse H2-kD (Biolegend, clone SF1-1.1, cat#116610, 1:200) at a concentration of 1:200 in FACS buffer for 20 min. Cells were washed three times after staining before being stained with DAPI. Data were collected using the BD FACSCanto II flow cytometer with BD FACSDIVA software (v8.0.1). The resulting data were analysed using FlowJo (version 10.6.1) and media fluorescence intensity of live, single cells was calculated.

### Treating cells with interferon-gamma (IFN-gamma) and flow cytometry for MHC-I

Active mouse IFN-gamma was sourced from Abcam (Cat#ab9922) and reconstituted in sterile water, as per the manufacturer’s instructions. Subclones (IE1 and IE2) and the parental 4T1 cell line were grown in a 24-well plate until ~70% confluence was achieved. Cells were then treated with IFN-gamma (100 ng/ml) for 24 h. Cells were stained with Alexa Fluor488 conjugated anti-mouse H2-kD (Biolegend, clone SF1-1.1, cat#116610, 1:200) at a concentration of 1:200 in FACS buffer for 20 min. Cells were washed three times before being stained with DAPI. Data were generated using the BD FCSCanto II flow cytometer with BD FACSDIVA software (v8.0.1). Analysis was carried out using FlowJo (version 10.6.1) and the median fluorescence intensity of live single cells was calculated.

### Dissociation of metastatic lung tissue and flow cytometry for MHC-I and PD-L1

Lungs were minced with scissors and dissociated in GentleMACS C tubes (Miltenyi Biotec) with collagenase (1 mg/ml, Type 1 A from clostridium histolyticum, Sigma, cat # C9891) in collagenase buffer (RPMI-1640 supplemented with 2.5% FBS and 1% HEPES), shaking at 37 °C for 50 min. After shaking, samples were passed through 70 um MACS SmartStrainers (Miltenyi Biotec) and washed with PBS, samples were treated with DNase (1 mg/ml, DNase 1 from bovine pancreas, Sigma, cat# CD25) for 3 min and quenched in FACS buffer (DPBS, Gibco, supplemented with 2% FBS and 2% 1 M HEPES). Samples were washed twice in FACS before Fc block with Mouse BD Fc Block (BD, 1:100, cat# 553141) for 15 min. Cells were stained with Alexa Fluor488 conjugated anti-mouse H2-kD (clone SF1-1.1, Biolegend, 1:200, cat# 116610) and APC conjugated anti-mouse CD274 (B7-H1, PD-L1; clone 10 F.9G2; Biolegend; 1:200, cat# 124311) in FACS buffer for 20 min. Samples were washed three times before being stained with DAPI. Data were generated using the BD LSR Fortessa flow cytometer with BD FACSDIVA software (v8.0.1). Analysis was carried out using FlowJo (version 10.6.1).

### Generation of poly-specific cytotoxic T lymphocyte lines

An established method was used^37^. Balb/c mice were immunised with 1 × 10^6^ irradiated 4T1 cells via intraperitoneal injection. Spleens were harvested >30 days post-immunisation and re-stimulated in vitro with irradiated 4T1 cells and cultured in RPMI containing 10% FBS and 10IU IL-2/mL for 14 days before experimental use.

### Functional assessment of tumour cell line immunogenicity

4T1 cell line variants were cultured in the absence or presence of 100 ng/mL recombinant IFN-gamma in 37 °C 5% CO_2_ for 24 h^38^. About 5 × 10^4^ treated cells were washed twice with PBS and incubated with 5 × 10^3^ 4T1-specific CTL in the presence of 10 µg/mL Brefeldin A in 37 °C 5% CO_2_ for 5 h. For anti-PD1 experiments: 4T1-specific CD8 + T cells were incubated in the presence of 10 µg/mL anti-PD1 (clone: RMP1-14, BioXcell, cat#BE0146) or isotype control (clone: 2A3, BioXcell, cat#BE0089) for 30 min, RT. Samples were then stained with cell surface antibodies for 20 min, 4 °C (α-CD8, clone: 53-6.7, 1:300, eBioscience), washed with PBS, fixed with 1% paraformaldehyde (15 min, RT, in the dark), permeabilized and stained for intracellular proteins (α-IFN-gamma, clone: XMG1.2, 1:300, eBioscience) in the presence of 0.4% Saponin for 30 min, 4 °C, and analysed on a FACSymphony A5 (BD).

### Statistics and reproducibility

No data were excluded from the analyses. No statistical method was used to predetermine the sample size of mouse experiments. Mice were randomised into treatment groups. The investigators were not blinded to allocation during experiments and outcome assessment.

### Reporting summary

Further information on research design is available in the Nature Research Reporting Summary linked to this article.

## Supplementary information

Supplementary Information

Peer Review File

Description of Additional Supplementary Files

Supplementary Data 1

Supplementary Data 2

Supplementary Data 3

Supplementary Data 4

Supplementary Data 5

Supplementary Data 6

Supplementary Data 7

Reporting Summary

Supplementary Figures

Supplementary tables

## Data Availability

All DNA barcode sequencing, RNA sequencing and whole genome sequencing FASTQs, intermediary files and resulting analysis files generated and used in this study have been deposited in the publicly available GEO database under accession code GSE210057. All raw data, intermediary files and resulting analysis files can be accessed at https://www.ncbi.nlm.nih.gov/geo/query/acc.cgi?acc=GSE210057. The remaining data were available within the manuscript, supplementary information or source data file. Mm10 (GRCm38.p4) reference genome was used for alignment and can be accessed at https://www.ncbi.nlm.nih.gov/assembly/GCF_000001635.24/ Source data are provided with this paper.

## Source data

Source Data

## Code availability

Code related to the analyses in this study can be found on GitHub at https://github.com/Swarbricklab-code/Baldwin-et-al-DNA-barcoding.
