# Supplementary material for: DNA barcoding reveals ongoing immunoediting of clonal cancer populations during metastatic progression and immunotherapy response
Source: Nat Commun. 2022 Nov 7;13:6539. doi: 10.1038/s41467-022-34041-x (PMC9640547; doi:10.1038/s41467-022-34041-x)
Supplement: Supplementary file 1 — Supplementary Information [file 41467_2022_34041_MOESM1_ESM.pdf]

BD FACSDiva 8.0.1

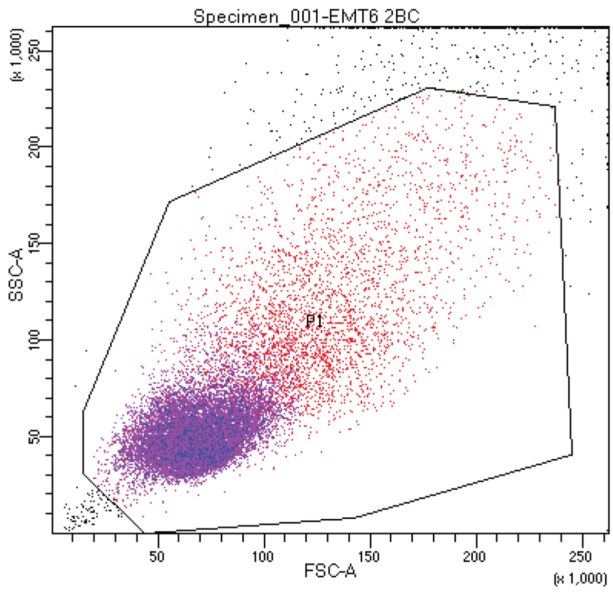

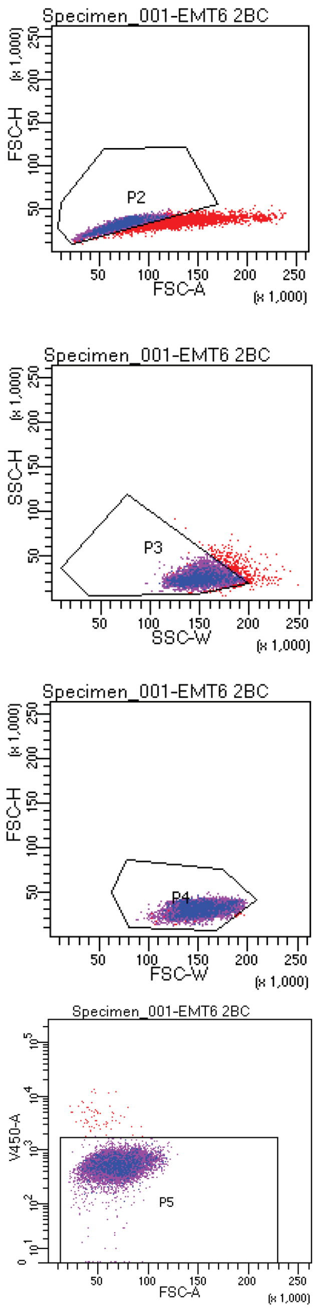

Tube: EMT6 2BC

Population	#Events	%Parent	%Total
All Events	20,000	####	100.0
P1	19,157	95.8	95.8
P2	16,133	84.2	80.7
P3	15,735	97.5	78.7
P4	15,719	99.9	78.6
P5	15,653	99.6	78.3
Live rFP pos	998	6.4	5.0

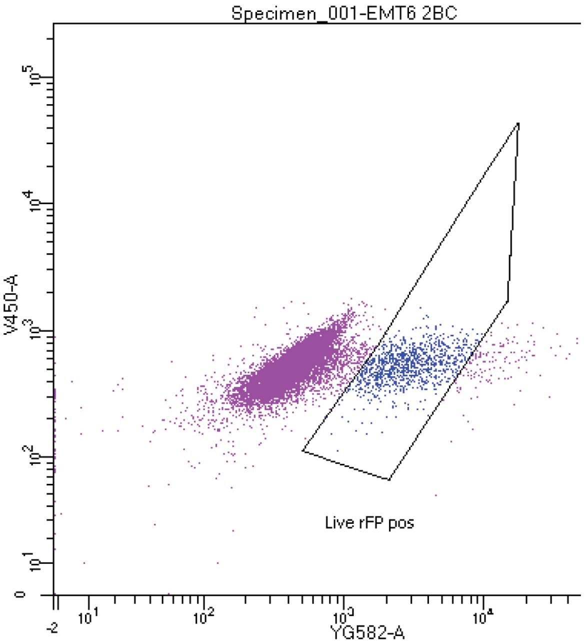

Supplementary Figure 1. Gating strategy showing isolation and selection of barcode transfected Red Fluorescent Protein (RFP) positive EMT6 cells.
Cells were isolated by first selecting all events and then excluding doublets. RFP is included as a reported gene in the barcode lentiviral cassette. As such, live, RFP positive cells were selected and sorted for further experimentation.

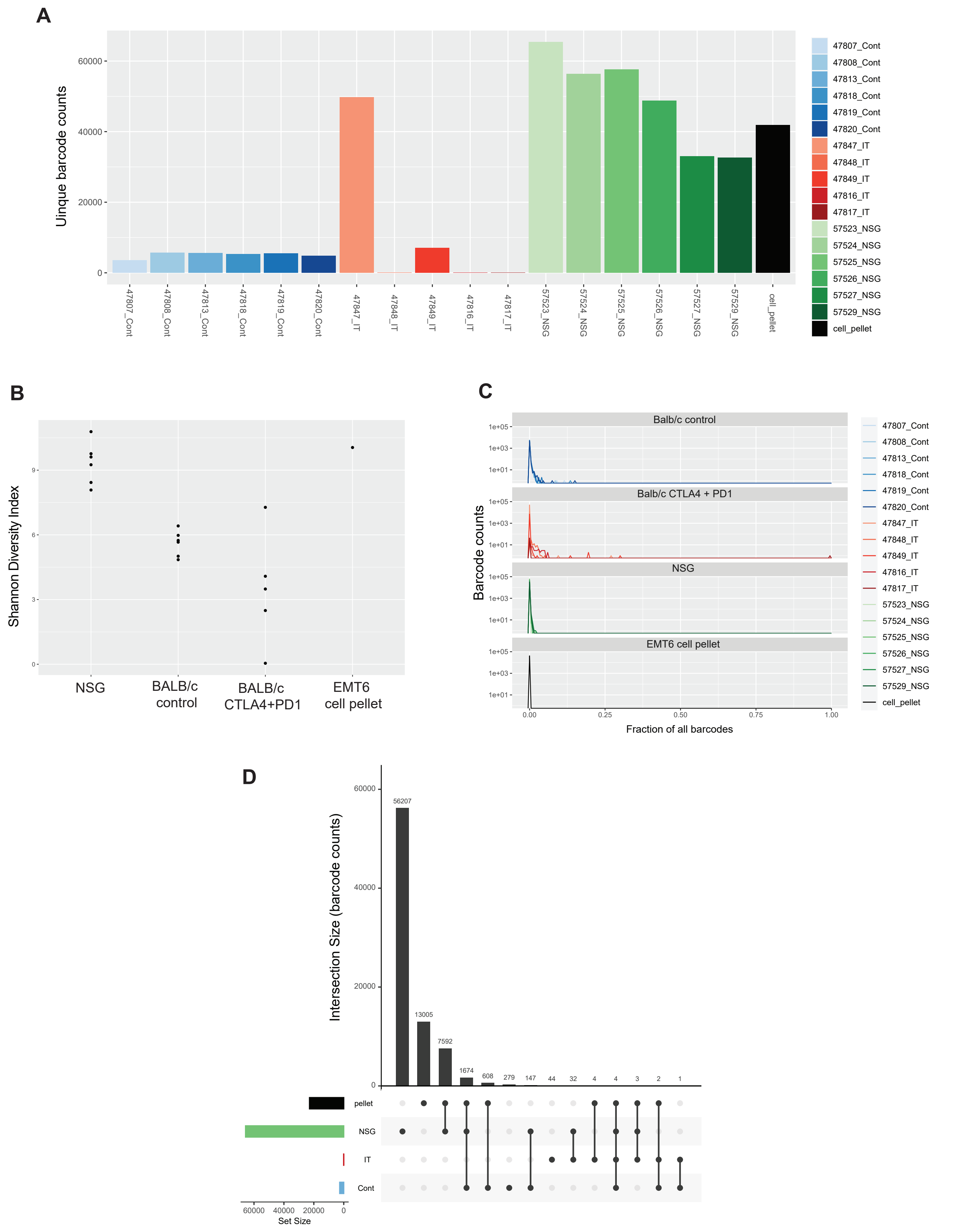

Supplementary Figure 2. Unique barcode counts and barcode distributions across EMT6 primary tumours and matching cell pellets. A. Unique barcode counts from EMT6 tumour bearing mice. B. Shannon diversity index, n=6 (NSG group), n=5 (Balb/c groups), n=1 for cell pellet. C. Distribution plots of barcode counts from individual mice, grouped by strain and treatment group. n=6 (NSG group), n=5 (Balb/c groups), n=1 for cell pellet D. Upset plot showing unique barcode counts and overlap between the EMT6cell pellet and tumour bearing NSG mice or immunocompetent immunotherapy treated (IT) or control treated (Cont) mice.

BD FACSDiva 8.0.1

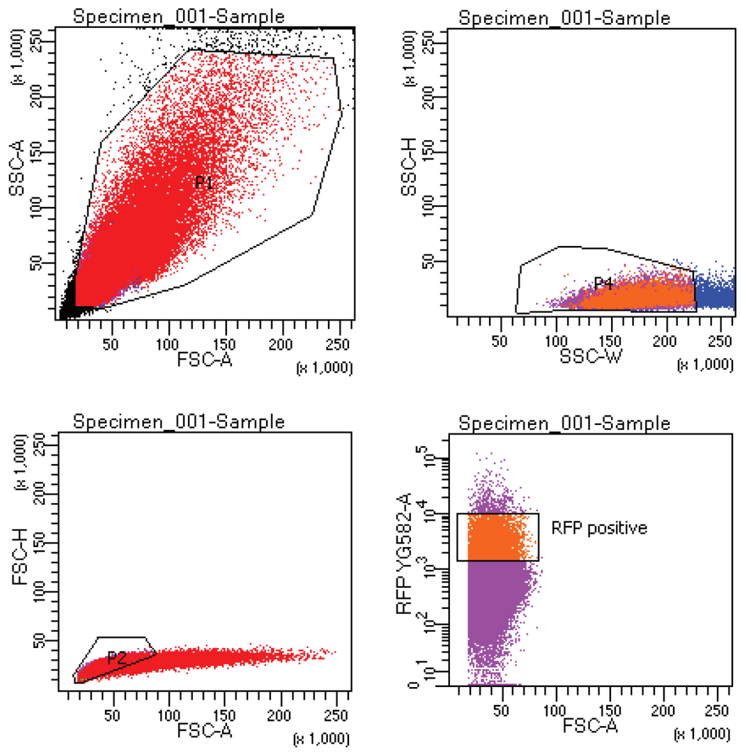

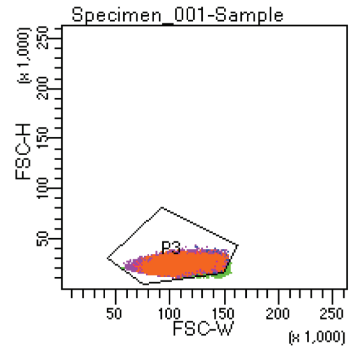

Tube: Sample

Population	#Events	%Parent	%Total
All Events	100,000	####	100.0
P1	93,547	93.5	93.5
P2	70,570	75.4	70.6
P3	70,224	99.5	70.2
P4	66,929	95.3	66.9
RFP positive	7,310	10.9	7.3

Supplementary Figure 3. Gating strategy showing isolation and selection of barcode transfected Red Fluorescent Protein (RFP) positive 4T1 cells.
Cells were isolated by first selecting all events and then excluding doublets. RFP is included as a reported gene in the barcode lentiviral cassette. As such, live, RFP positive cells were selected and sorted for further experimentation.

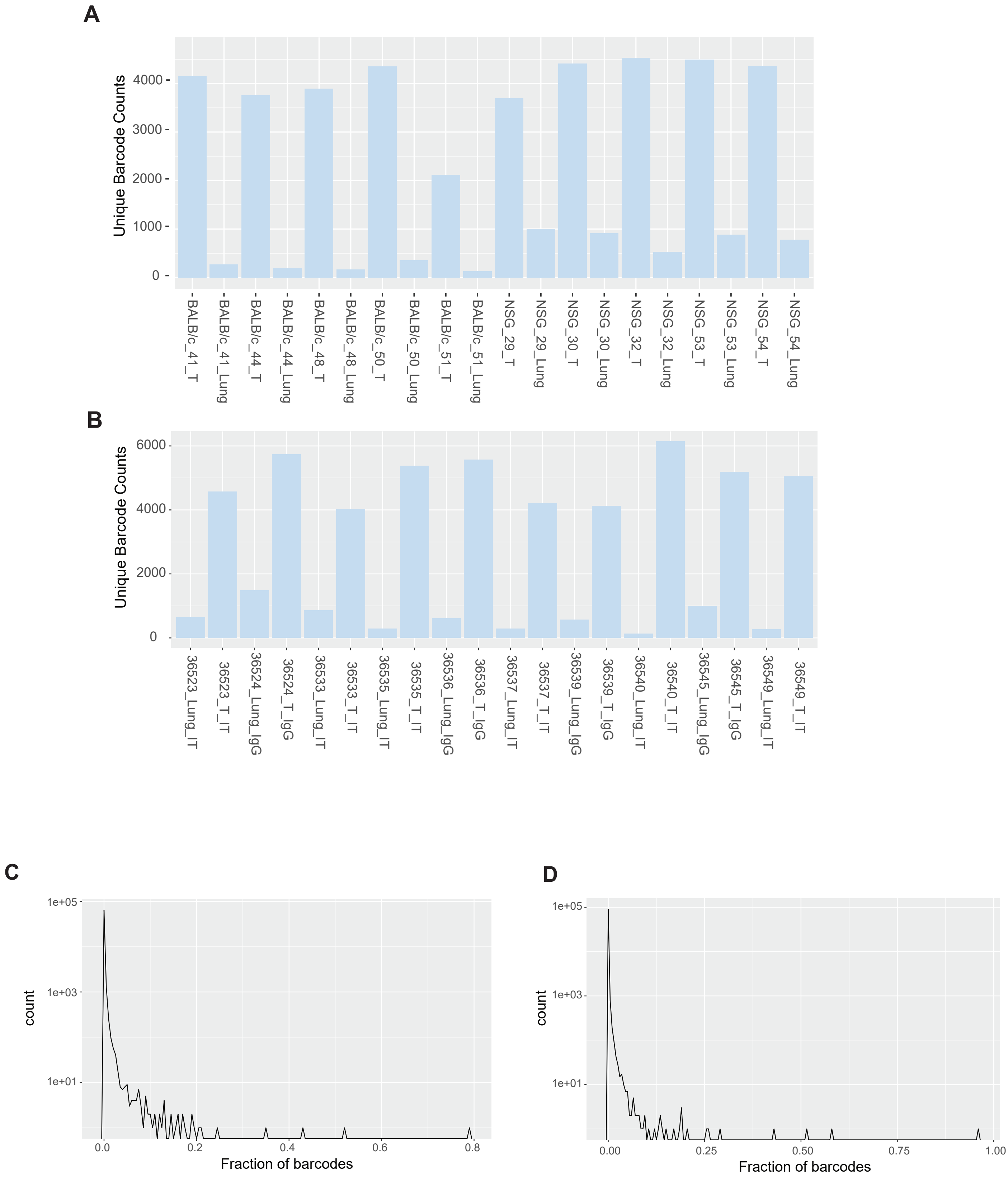

Supplementary Figure 4. Unique barcode counts and barcode distributions across 4T1 primary tumours and matched lungs in NSG and Balb/c mice. A. Unique barcode counts detected in matched primary tumour (T) or lungs (Lung) from 4T1 tumour bearing NSG or Balb/c mice. B. Unique barcode counts detected in matched primary tumour (T) and lungs (Lung) from 4T1 tumour bearing mice treated with either combined immunotherapy (IT) or control antibodies (IgG) C. Distribution plot of barcode counts from all (NSG and Balb/c) mice. D. Distribution plot of barcode counts from Balb/c mice treated with combination immunotherapy or control antibodies.

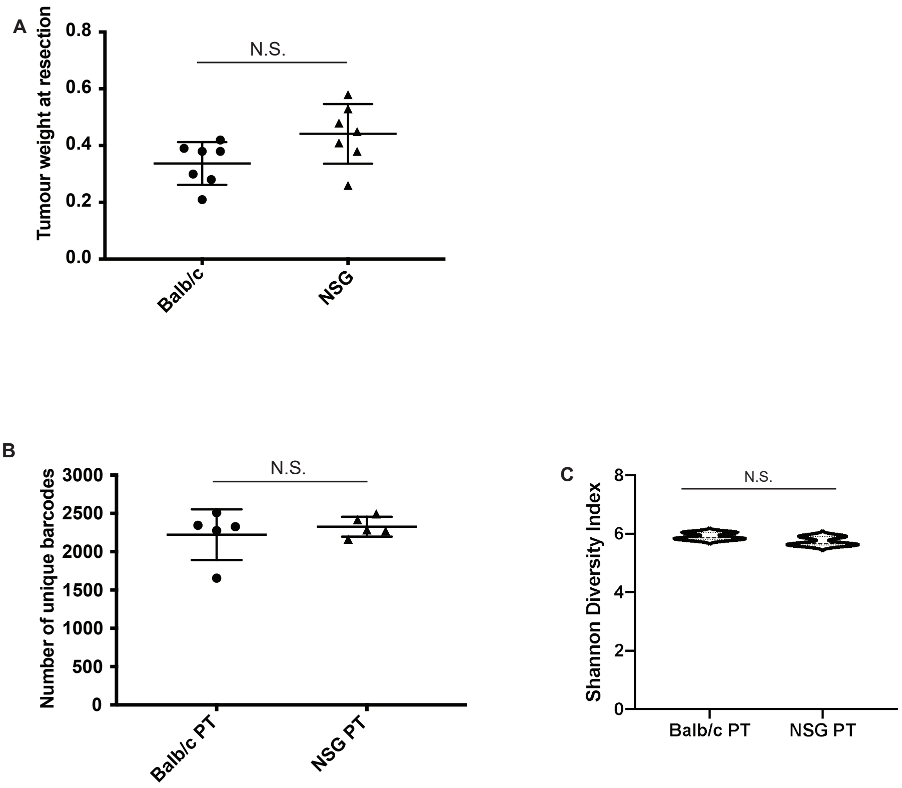

Supplementary Figure 5. 4T1 primary tumour growth and barcode diversity in primary tumours. A. Primary tumour mass. 7 mice per group. Data shown as mean +/- SD, two sided T test to calculate significance. B. Total number of unique barcodes identified in primary tumours (PT) of Balb/c or NSG mice. 5 mice per group. Data shown as mean +/- SD, two sided T test to calculate significance C. Shannon diversity analysis comparing primary tumours (PT) from Balb/c and NSG mice. 5 mice per group. Statistical significance calculated using two sided T test.

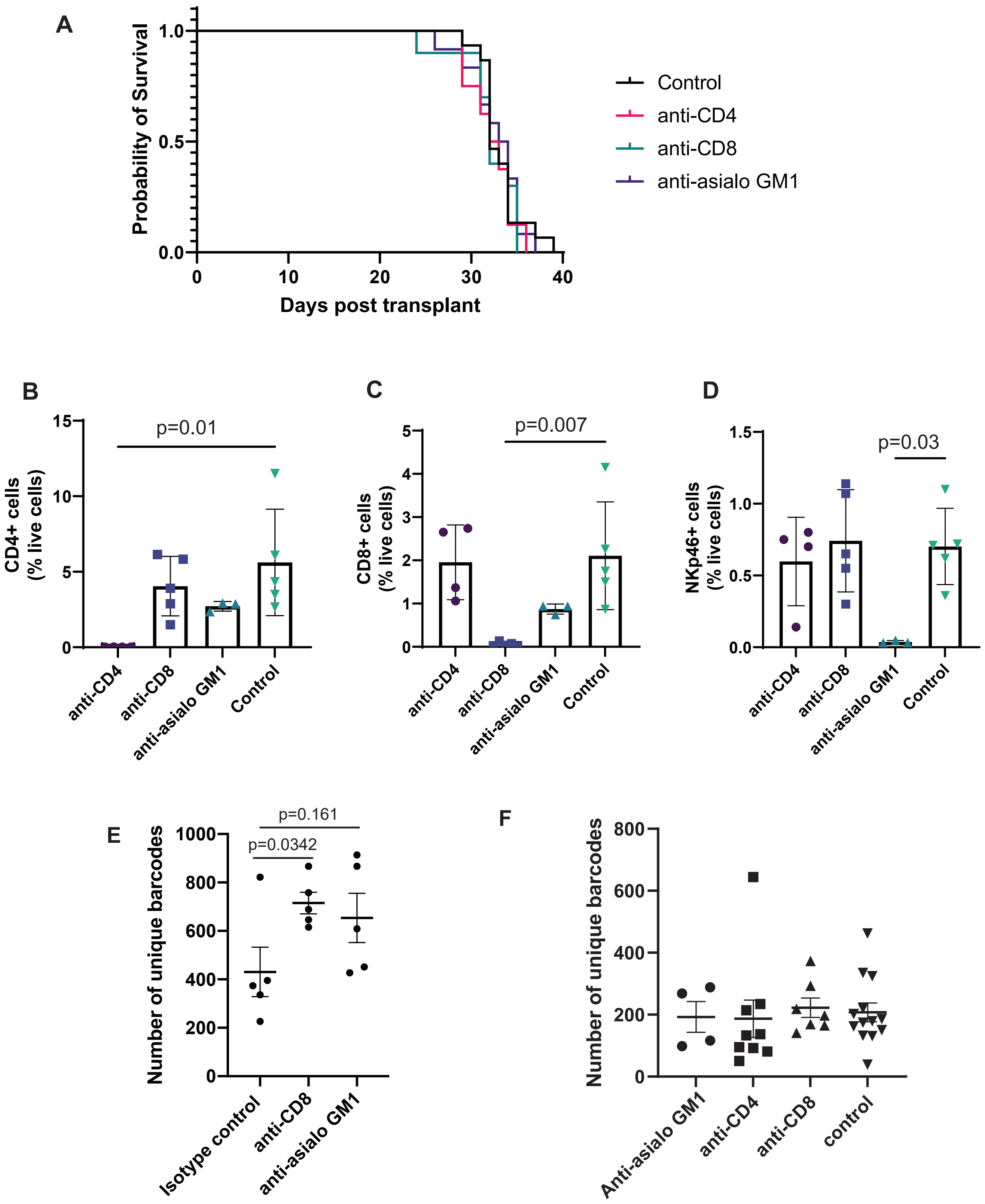

Supplementary Figure 6. Depletion of CD8+ T cells with anti-CD8, CD4+ T cells with anti-CD4 or NK cells with anti-asialo GM1 does not significantly affect survival or barcode diveristy of mice bearing 4T1 tumours. A. Kaplan-Meier survival analysis of Balb/c mice transplanted with 4T1 cells, primary tumour was resected on day 15. Depletion of target cell types was initiated 1 day prior to resection, on day 14. n=15 mice (control), n=8 mice (anti-CD4), n=10 mice (anti-CD8), n=12 mice (anti-asialo GM1). B-D. Flow cytometry to confirm depletion of target cell types. Buffy coat was collected on day 22, 2 days after the final dose of depleting antibodies was given. One way ANOVA with Tukey's HSD for multiple comparisons. n=4 mice (anti-CD4), n=5 mice (anti-CD8), n=3 mice (anti-asialo GM1), n=5 mice (control). Data shown as mean +/- SD. E. Initial experiments showed a small increase in unique barcode number in the lungs when mice were treated with CD8-depleting antibodies. One way ANOVA with Tukey's HSD correction for multiple comparisons. 5 mice per group. Data shown as mean +/- SEM. F. Repeated experiments showed depletion of key cell types does not significantly change barcode number in the lungs at endpoint. n=4 mice (anti-asialo GM1), n=9 mice (anti-CD4), n=7 mice (anti-CD8), n=13 mice (control). Data shown as mean +/- SEM

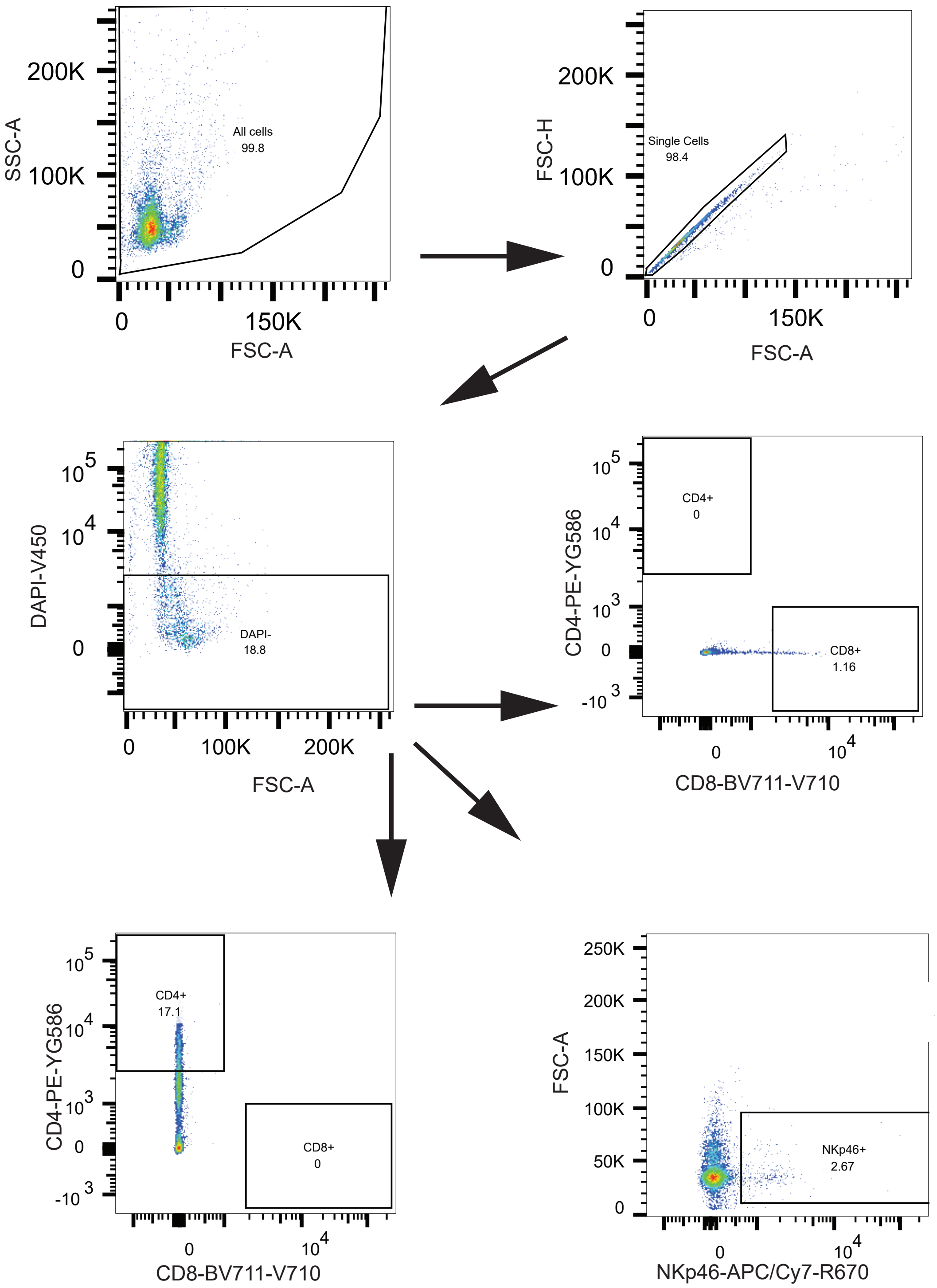

Supplementary Figure 7.Gating strategy to confirm depletion of target cell types in 4T1 tumour bearing mice, treated with either anti-CD4, anti-CD8 or anti-asialo GM1.

Buffy coat samples were analysed via flow cytometry. Live, single cells were isolated and the expression of CD4, CD8 and NKp46 was recorded.

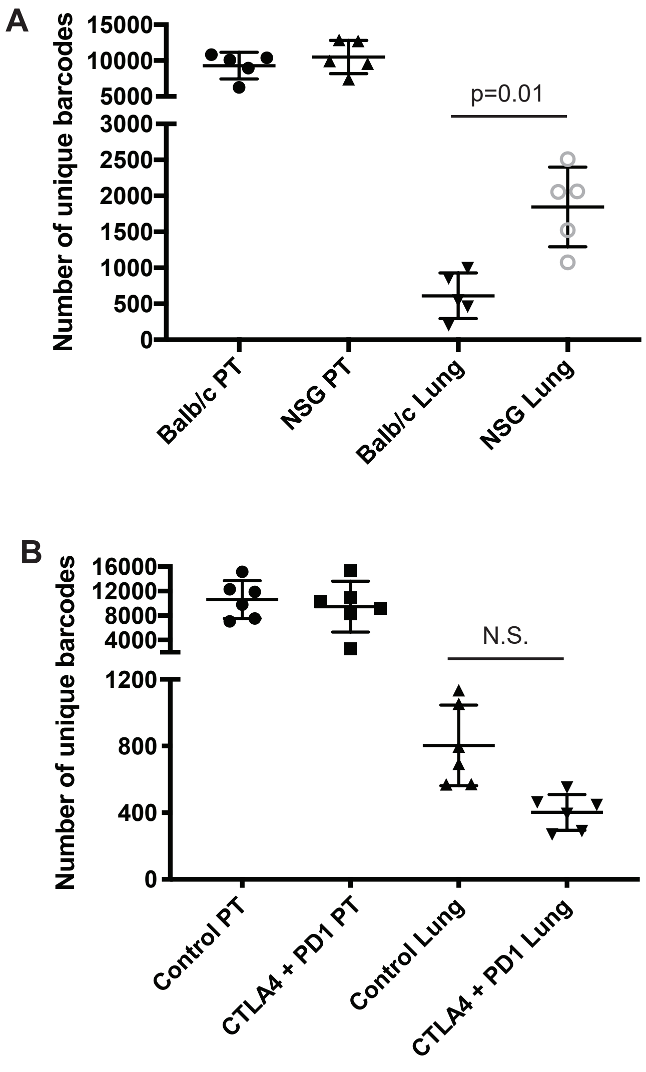

Supplementary Figure 8. Analysis of changes in barcode proportions in the 4T1 cells with the 300 000 barcode library replicates the 5000 barcode library. A. Number of unique barcodes identified in 4T1 primary tumours and lung metastases grown in NSG mice or Balb/c mice. GLM fit with Tukey's HSD for multiple comparisons. n= 5 mice per group. Data shown as mean +/- SD B. Number of unique barcodes identified in 4T1 primary tumours (PT) and lung metastases (Lung) grown in Balb/c mice treated with isotype control antibodies or anti-PD1 + anti-CTLA4. A trend to decreasing unique barcode number is seen in anti-PD1 and anti-CTLA4 treated lungs, although this does not reach significance. GLM fit with Tukey's HSD for multiple comparisons. 5 mice in control group, 5 mice in all other groups. Data shown as mean +/- SD.

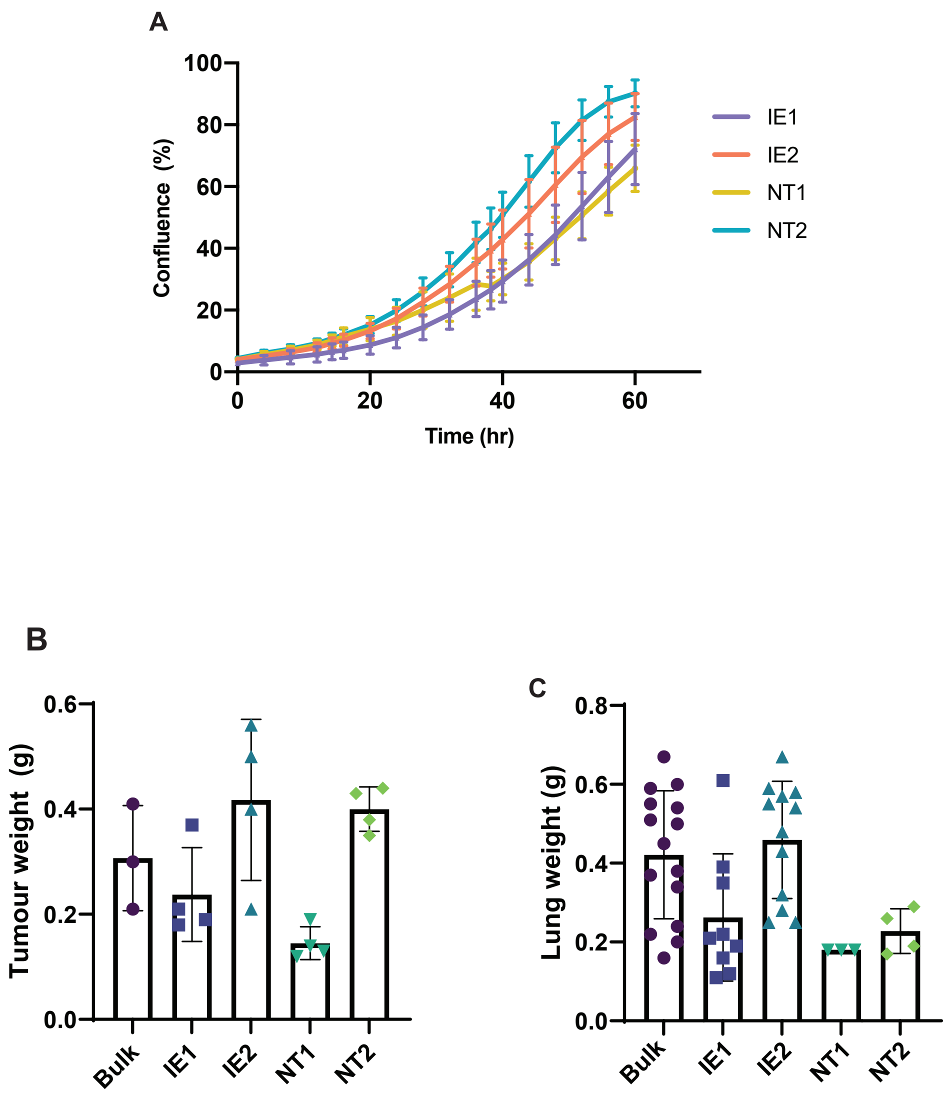

Supplementary Figure 9. Kinetics of 4T1 clonal cell lines in vitro and in vivo. A: Growth kinetics in vitro as measured by percentage confluence over time (hr).3 biological replicate cell lines per group. Data shown as mean +/- SD. B: Tumour weight at resection generated from clonal cell lines transplanted into BALB/c mice. n=3 (bulk), n=4 (all other groups). Data shown as mean +/- SD C: Lung weight at endstage of untreated subclone-tumour bearing BALB/c mice. n=15 mice (bulk), n=9 mice (IE1), n=12 mice (IE2), n=3 mice (NT1), n=4 mice (NT2). Data shown as mean +/- SD.

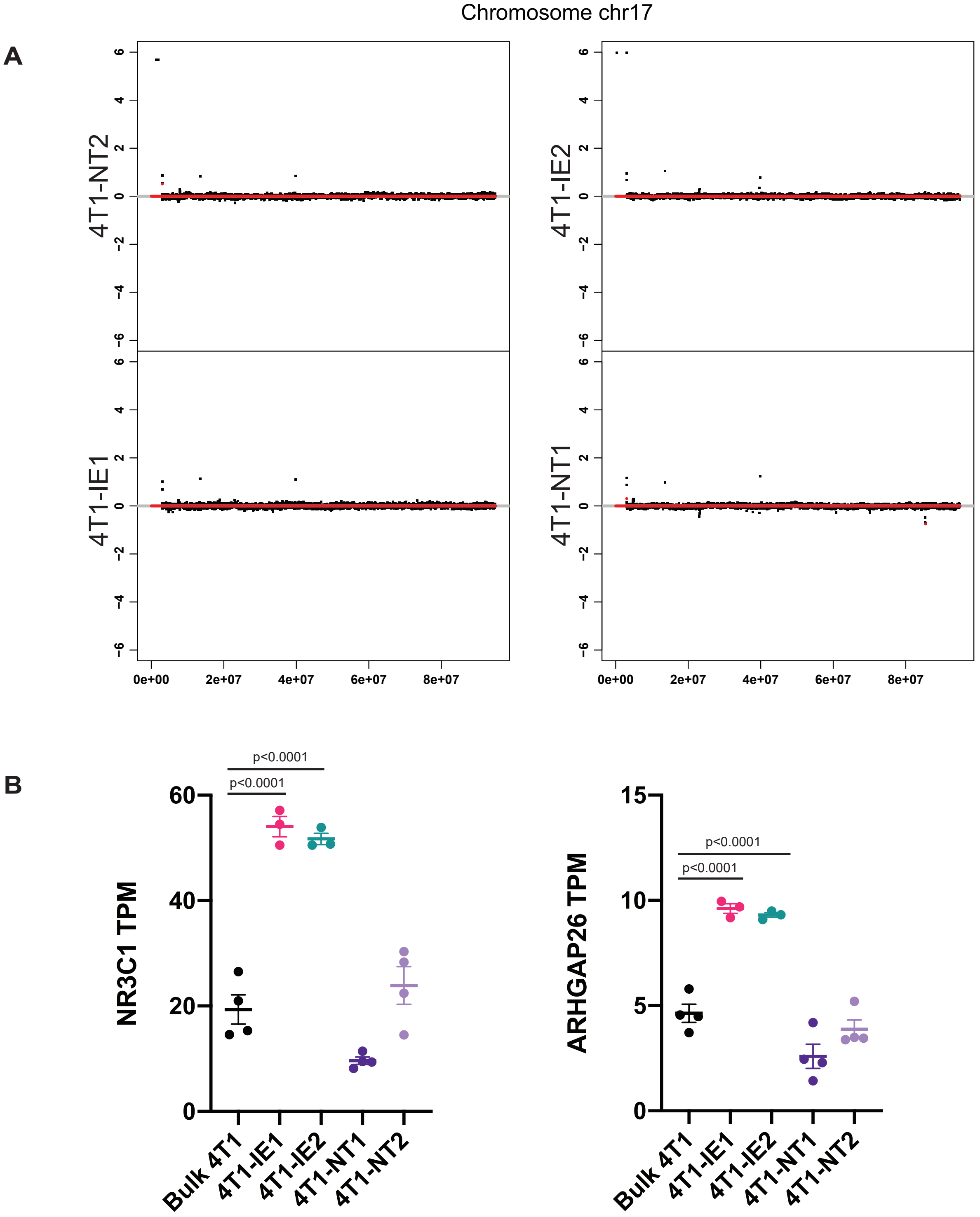

Supplementary Figure 10. Genomic alterations do not appear to explain phenotypes of the immune evasive clones A. Genome copy number at the MHC-I locus on chromosome 17 of indicated clones. Chromosomal location is indicated on the x-axis and level of copy number alteration is indicated on the y-axis. B. Expression levels of the two genes that have a single copy number increase in IE1 and IE2 in indicated cell populations. N=3 biological replicate cell lines in IE1 and IE2 groups, 4 biological replicates in bulk, NT1 and NT2 groups. Data presented as transcripts per million (TPM) mean +/- SEM. Significance was calculated using a one-way ANOVA with Tukey's HSD for multiple comparisons.

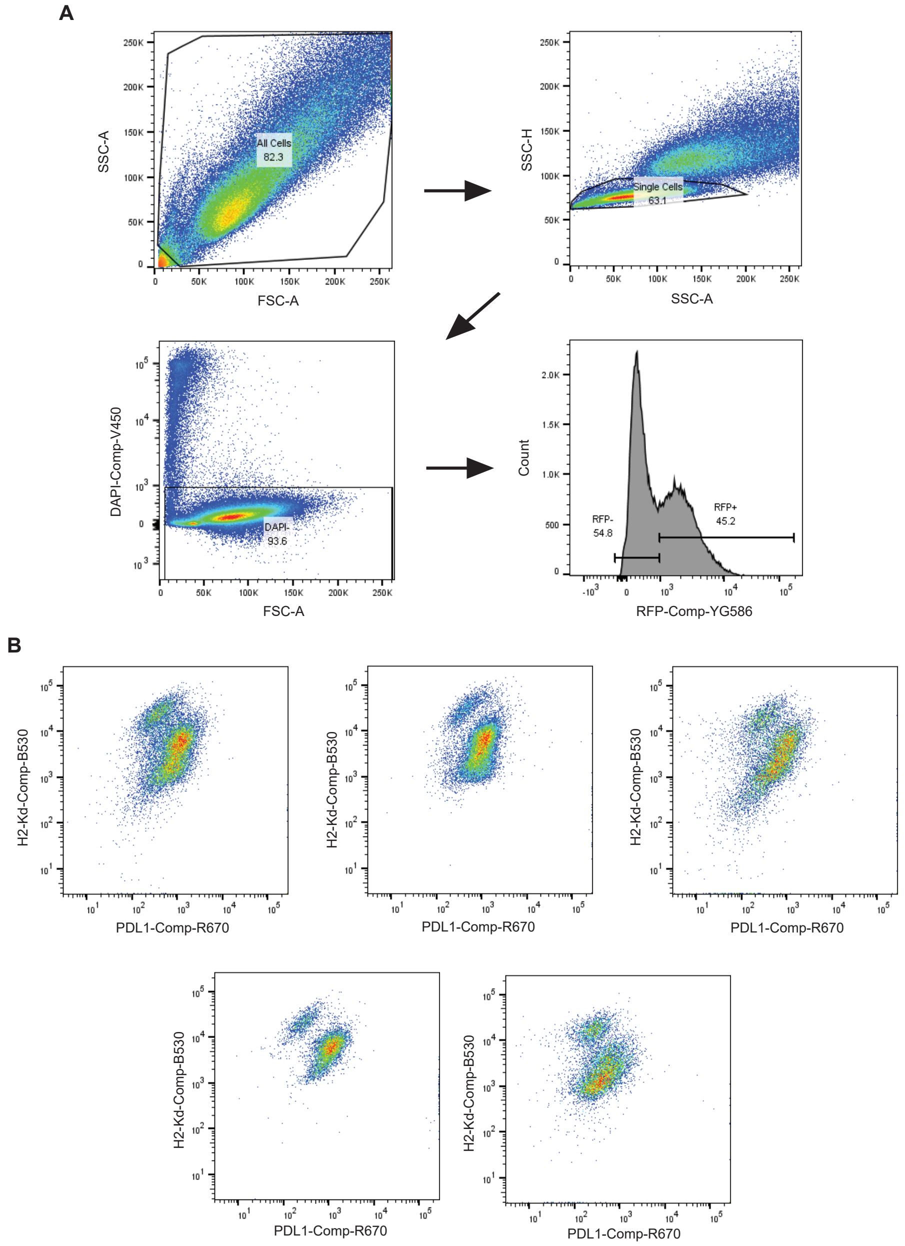

Supplementary Figure 11. Distinct populations of subclones with varying PD-L1 and MHC I expression exist in immunotherapy treated end-stage lungs. A: Gating strategy. B: PD-L1 and MHC I expression of RFP+ barcoded cancer cells isolated from endstage 4T1 immunotherapy treated lungs. Each plot represents an individual mouse. n=5

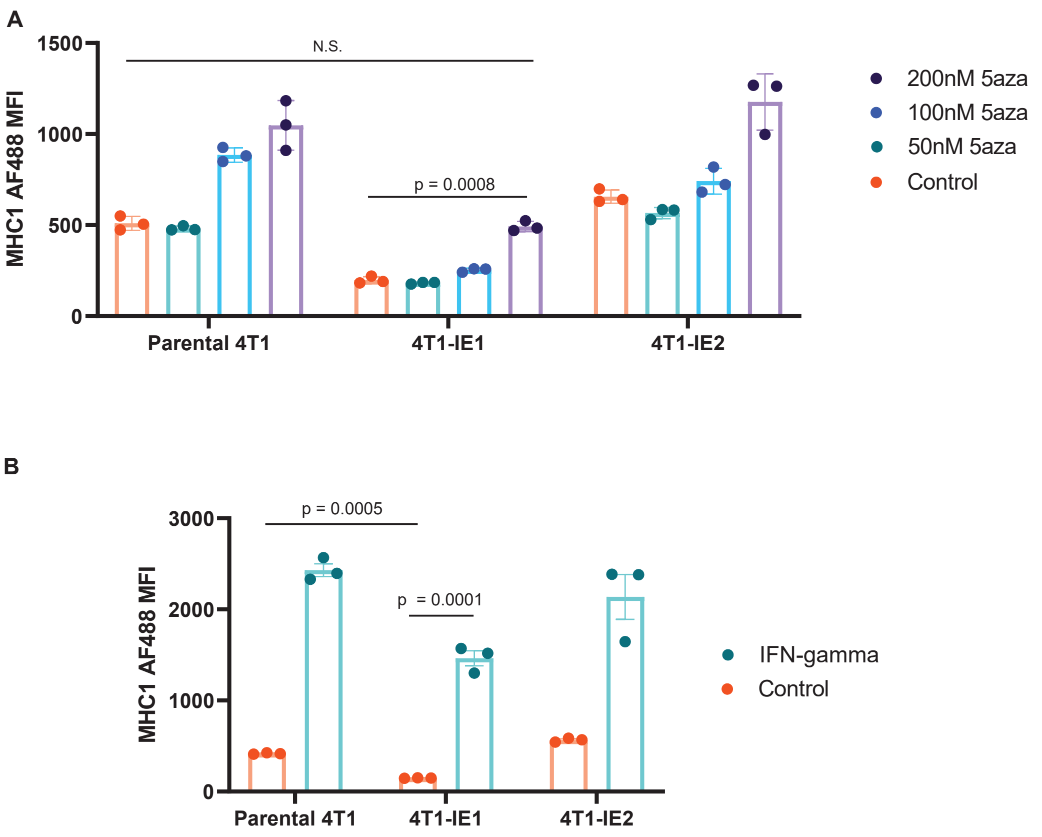

Supplementary Figure 12. Regulation of MHC expression by 5'-aza-2'-deoxycytidine (5-aza) and IFN-gamma in the clonal cell lines. A. MHC-I protein expression measured by flow cytometry in indicated cell lines treated with indicated concentrations of 5-aza. Two-way ANOVA with Tukey's HSD for multiple corrections. Data shown as mean +/- SEM. 3 biological replicates per group. B. MHC-I protein expression in indicated cell lines without or with IFN-gamma treatment. One way ANOVA with Tukey's HSD for multiple corrections. Data shown as mean +/- SEM. 3 biological replicates per group.

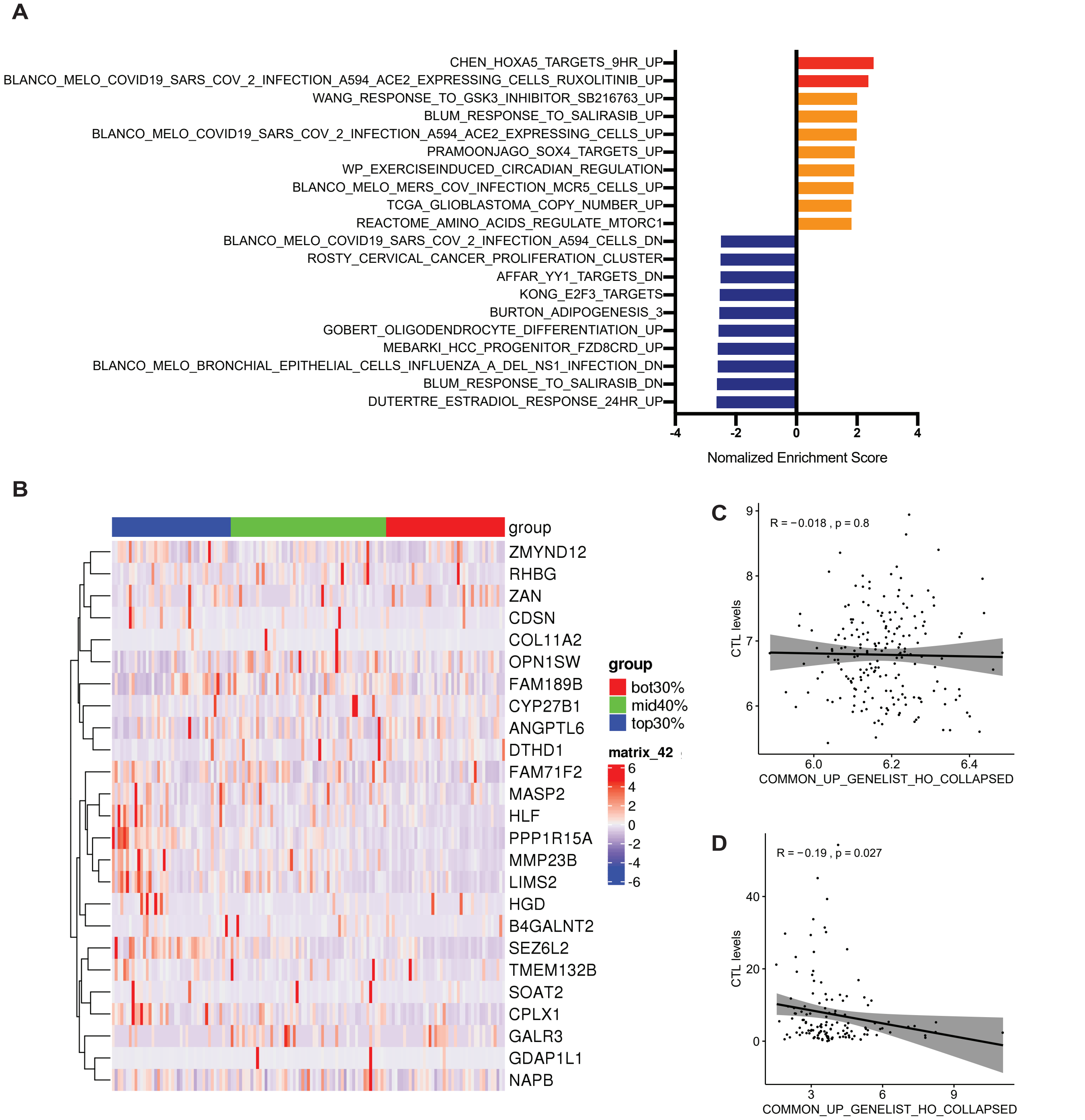

Supplementary Figure 13. Common gene signature from immunotherapy resistant clones. A. Gene-set enrichment analysis of the overlapping genes between the IE1 and IE2 clones. Red indicates upregulated gene-sets with a significant FDR q-value when multiple testing is taken into account, orange indicates significant upregulated gene-sets with a nominal p value, and blue indicates downregulated gene-sets with a significant FDR q-value. B. Heatmap of unsupervised hierarchical clustering of the immunotherapy resistance signature genes in the TCGA breast cancer dataset, with tumours grouped based on top 30%, middle 40%, and bottom 30% overall gene signature score. C. Correlation plot of immunotherapy resistance signature score with cytotoxic T lymphocyte (CTL) levels in the METABRIC dataset. Error bands indicate 95% confidence interval. D. Correlation plot of immunotherapy resistance signature score with CTL levels in the TCGA dataset. Error bands indicate 95% confidence interval.
