# Supplementary material for: DNA barcoding reveals ongoing immunoediting of clonal cancer populations during metastatic progression and immunotherapy response
Source: Nat Commun. 2022 Nov 7;13:6539. doi: 10.1038/s41467-022-34041-x (PMC9640547; doi:10.1038/s41467-022-34041-x)
Supplement: Supplementary file 3 — Description of Additional Supplementary Files [file 41467_2022_34041_MOESM3_ESM.pdf]

File Name: Supplementary data 1

Description: Annotated copy number variations (CNV) found within all subclones. By analysing whole genome sequencing data in R using the cn.mops package, copy number variations could be determined. No major copy number aberrations were detected across the clones, although a single copy number gain was detected in IE1, IE2 and NT2.

File Name: Supplementary data 2

Description: List of genes differentially expressed in IE1 compared to parental 4T1 bulk. Differentially expressed genes (DEGs) were generated by analysing bulk RNA sequencing data using R and the EdgeR package with Benjamini-Hochberg multiple testing corrections. DEGs were filtered for significance based on a FDR <0.05.

File Name: Supplementary data 3

Description: List of genes differentially expressed in IE2 compared to parental 4T1 bulk. Differentially expressed genes (DEGs) were generated by analysing bulk RNA sequencing data using R and the EdgeR package with Benjamini-Hochberg multiple testing corrections. DEGs were filtered for significance based on a FDR <0.05. A greater number of differentially expressed genes were detected in IE2 than IE1.

File Name: Supplementary data 4

Description: All significantly enriched gene sets found in IE1. Differentially expressed genes generated from comparing IE1 to bulk were preranked by fold change before searching for gene set enrichment using the Molecular Signature Database (MSigDB) across all available collections. Gene sets were filtered for significance based on a FDR<0.05.

File Name: Supplementary data 5

Description: All significantly enriched gene sets found in IE2. Differentially expressed genes generated from comparing IE2 to bulk were preranked by fold change. Gene set enrichment was carried out using Molecular Signature Database (MSigDB) across all available collections. Gene sets were filtered for significance based on a FDR<0.05

File Name: Supplementary data 6

Description: List of common differentially expressed genes found in IE1 and IE2. Commonly differentially expressed genes (DEGs) were determined by overlapping significant DEGs in IE1 and IE2 by gene name. An average fold change was calculated across the two samples.

File Name: Supplementary data 7

Description: Top significantly enriched gene sets found from common differentially expressed genes in IE1 and IE2. The fold change of the common differentially expressed genes in IE1 and IE2 were averaged together to generate an average fold change across both IE1 and IE2. The gene list was then preranked before searching for gene set enrichment using Molecular Signature Database (MSigDB) across the C2 All collection. Gene sets were filtered for significance based on a $FDR<0.05$. The majority of significant gene sets that were returned were negatively enriched in IE1 and IE2.
