# Supplementary material for: DNA barcoding reveals ongoing immunoediting of clonal cancer populations during metastatic progression and immunotherapy response
Source: Nat Commun. 2022 Nov 7;13:6539. doi: 10.1038/s41467-022-34041-x (PMC9640547; doi:10.1038/s41467-022-34041-x)
Supplement: Supplementary file 11 — Reporting Summary [file 41467_2022_34041_MOESM11_ESM.pdf]

nature portfolio

Corresponding author(s): Alexander Swarbrick

Last updated by author(s): Sept 26, 2022

Reporting Summary

Nature Portfolio wishes to improve the reproducibility of the work that we publish. This form provides structure for consistency and transparency in reporting. For further information on Nature Portfolio policies, see our Editorial Policies and the Editorial Policy Checklist.

Statistics

For all statistical analyses, confirm that the following items are present in the figure legend, table legend, main text, or Methods section.

n/a	Confirmed	
☐	☒	The exact sample size (*n*) for each experimental group/condition, given as a discrete number and unit of measurement
☐	☒	A statement on whether measurements were taken from distinct samples or whether the same sample was measured repeatedly
☐	☒	The statistical test(s) used AND whether they are one- or two-sided<br>*Only common tests should be described solely by name; describe more complex techniques in the Methods section.*
☒	☐	A description of all covariates tested
☐	☒	A description of any assumptions or corrections, such as tests of normality and adjustment for multiple comparisons
☐	☒	A full description of the statistical parameters including central tendency (e.g. means) or other basic estimates (e.g. regression coefficient) AND variation (e.g. standard deviation) or associated estimates of uncertainty (e.g. confidence intervals)
☐	☒	For null hypothesis testing, the test statistic (e.g. *F*, *t*, *r*) with confidence intervals, effect sizes, degrees of freedom and *P* value noted<br>*Give P values as exact values whenever suitable.*
☒	☐	For Bayesian analysis, information on the choice of priors and Markov chain Monte Carlo settings
☒	☐	For hierarchical and complex designs, identification of the appropriate level for tests and full reporting of outcomes
☒	☐	Estimates of effect sizes (e.g. Cohen's *d*, Pearson's *r*), indicating how they were calculated

Our web collection on statistics for biologists contains articles on many of the points above.

Software and code

Policy information about availability of computer code

Data collection	Flow cytometry data was collected using BD FACSDIVA version 8.0.1. Barcode sequencing data collection utilised Illumina's BaseSpace software.
Data analysis	GraphPad Prism 9 was used for statistical analysis and data visualization.<br>Python 2.7.12 was used for initial barcode data analysis, using scripts from Bhang et al. 2015 Nature Medicine (ClonTracer 1.2).<br>Basespace v5.3.1 and v6.2 was used.<br>Open source R was used for analysis of sequencing data, version 3.6.1, 4.0.2. Packages used include EntropyExplorer)v1.1), fishplot (v0.5.1), UpSetR (v1.4.0), RcolorBrewer (v1.0.12), Pheatmap (v1.0.12), tidyverse (v1.3.0), survival (v3.2.7), biomaRt (v2.5)<br>Geneset enrichment analysis was carried out using the GSEA desktop app v4.1.0 and MsigDB (v7.5.1).<br>Transcriptome analysis utilised mm10, mouse transcriptome (Gencode M9, GRCm38.p4), STAR aligner (v2.4.1d), RSEM (v1.2.18), EdgeR (v.3.3.8).<br>Whole Genome analysis used cn.mops (v1.4.2), BWA (v0.7.8), novosort (v1.03.8) and NCBI BLAST v2.9.<br>Flow Cytometry gating and analysis was performed using FlowJo 10.6.1 and FACSDIVA v8.0.1.

For manuscripts utilizing custom algorithms or software that are central to the research but not yet described in published literature, software must be made available to editors and reviewers. We strongly encourage code deposition in a community repository (e.g. GitHub). See the Nature Portfolio guidelines for submitting code & software for further information.

Data

Policy information about availability of data

All manuscripts must include a data availability statement. This statement should provide the following information, where applicable:

- Accession codes, unique identifiers, or web links for publicly available datasets
- A description of any restrictions on data availability
- For clinical datasets or third party data, please ensure that the statement adheres to our policy

RNA-seq data have been deposited in the NCBI Gene Expression Omnibus (GEO) under accession number XX.
DNA-seq data (whole genome and barcoding) have been deposited in NCBI Gene Expression Omnibus (GEO) under accession number XX.
mm10 (GRCm38.p4) was used for genome aligments. The remaining data are available within the article, supplementary information, or source data file.

Field-specific reporting

Please select the one below that is the best fit for your research. If you are not sure, read the appropriate sections before making your selection.

[x] Life sciences [ ] Behavioural & social sciences [ ] Ecological, evolutionary & environmental sciences

For a reference copy of the document with all sections, see nature.com/documents/nr-reporting-summary-flat.pdf

Life sciences study design

All studies must disclose on these points even when the disclosure is negative.

Sample size	No statistical calculation was carried out to inform sample size of in vivo experiments. In vitro studies were performed with at least 3 biological replicates to ensure reproducibility.
Data exclusions	No data was excluded from this study.
Replication	All experiments that were replicated produced equivalent results and this is indicated in the figure legends. The major findings of the effect of the immune system and immunotherapy on clonal diversity in the 4T1 model was reproduced with two independent pools of barcoded cells as indicated in the manuscript. RNAseq analysis was performed on 3-4 independent biological replicates for each of the clonal cell lines as well as bulk parental cells.
Randomization	Mice were randomised into treatment arms based on tumour volume determined by caliper measurements. Treatment groups were generated so that they all had similar average tumour volumes at the initiation of treatment.
Blinding	Blinding was not performed during in vivo experiments as researchers were unable to influence the barcode diversity of the samples. During the bioinformatic analysis of the barcoding data the researchers were blinded to the groupings until the analysis was complete.

Reporting for specific materials, systems and methods

We require information from authors about some types of materials, experimental systems and methods used in many studies. Here, indicate whether each material, system or method listed is relevant to your study. If you are not sure if a list item applies to your research, read the appropriate section before selecting a response.

Materials & experimental systems

n/a	Involved in the study	
[ ]	[x]	Antibodies
[ ]	[x]	Eukaryotic cell lines
[x]	[ ]	Palaeontology and archaeology
[ ]	[x]	Animals and other organisms
[x]	[ ]	Human research participants
[x]	[ ]	Clinical data
[x]	[ ]	Dual use research of concern

Methods

n/a	Involved in the study	
[x]	[ ]	ChIP-seq
[ ]	[x]	Flow cytometry
[x]	[ ]	MRI-based neuroimaging

Antibodies

Antibodies used	Antibodies for in vivo experiments:<br>BioXCell InVivoMAb anti-mouse CTLA-4 (CD152) Clone UC10-4F10-11 Cat# BE0032, 200ug per mouse administered via intrapertoneal injection in PBS.<br>BioXCell InVivoMAb polyclonal Armenian hamser IgG (Clone N/A) Cat# BE0091, 200ug per mouse administered via intrapertoneal injection in PBS

	BioXCell InVivoMab anti-mouse PD-1 (CD279) Clone RMP1-14 Cat# BE0146, 200ug per mouse administered via intrapertoneal injection in PBS.<br>BioXCell InVivoMAb rat IgG2a isotype control, anti-trinitrophenol Clone 2A3 Cat# BE0089, 200ug per mouse administered via intrapertoneal injection in PBS<br>BioXCell InVivoMAb anti-mouse CD8 (Lyt3.2) Clone 53-5.8 Cat# BE0223 Lot 666318F1, 100ug per mouse administered via intraperitoneal injection in PBS<br>BioXCell InVivoMAb anti-mouse CD4 Clone GK1.5 Cat# BE0003-1 Lot 798421M1B, 100ug per mouse administered via intraperitoneal injection in PBC<br>Novachem anti-Asialo-GM1 SKU# 986-10001 Lot KQF6371, 100ug per mouse administered via intraperitoneal injection in PBS<br><br>Antibodies for flow cytometry:<br>BioLegend Alexa Fluor 488 anti-mouse H-2kd antibody Clone SF1-1.1 Cat# 116610 Lot# B260805 used at 1:200 dilution.<br>Biolegend APC anti-mouse CD274 (B7-H-1, PD-L1) antibody Clone 10F.9G2 Cat# 124311 Lot# B277023 used at 1:200 dilution.<br>BioLegend BV711 anti-mouse CD8a antibody, clone 53-6.7, Cat# 100759, used at 1:200 dilution<br>Biolegend PE anti-mouse CD4 antibody, clone RM4-5, Cat# 100511, used at 1:200 dilution.<br>Biolegend APC anti-mouse NKp46/CD335 antibody, clone 29A1.4, Cat# 137608, used at 1:200 dilution
Validation	Antibodies for in vivo experiments:<br>Antibodies were not validated in house after purchase. Antibodies were obtained from BioXCell. This manufacturer guarantees high purity antibodies which are validated by binding bioassays, and also provide matching isotype control antibodies. These antibodies have been widely used and referenced in other peer reviewed publications which can be found in the manufacturer's website. Manufacturer validation utilitises a known recombinant protein library to ensure each antibody binds specifically to target<br>Antibodies for flow cytometry:<br>Antibodies used for flow cytometry are all commercially available and have been used in other peer reviewed publications (linked on the supplier's website). Manufacturer's validation includes testing on known negative and known positive cell lines to ensure specific binding and comparison with other commercially available clones.

Eukaryotic cell lines

Policy information about cell lines

Cell line source(s)	All cell lines were obtained from ATCC. Cell lines used were 4T1 and EMT6.
Authentication	Cell lines were obtained from ATCC, minimally passaged and expanded before being frozen down in bulk. Low passage number cells were used for these experiments. No further cell line authentication was performed, but ATCC provides cell line authentication prior to dispatch.
Mycoplasma contamination	All cell lines were tested for mycoplasma contamination and returned a negative result.
Commonly misidentified lines<br>(See ICLAC register)	None used

Animals and other organisms

Policy information about studies involving animals; ARRIVE guidelines recommended for reporting animal research

Laboratory animals	Female BALB/cJAusb mice at 6-8 weeks and female NOD.Cg-Prkdcscid Il2rgtm1Wjl/SzJ (NSG) mice at 6-8 weeks. All mice were sourced from Australian BioResources (Moss Vale, Australia). Mice were housed in an environment with regulated temperature, water and food ad libitum and a 12 hour light/dark cycle. Temperature is controlled at 21 degrees C (+/-1deg.) with humidity maintained between 50-60%.
Wild animals	No wild animals were used in these studies.
Field-collected samples	No field collected samples were used in these studies.
Ethics oversight	All animal experiments were approved by the Garvan Institute of Medical Research/St. Vincent's Hospital Animal Experimentation Ethics Committee, approval 19/04

Note that full information on the approval of the study protocol must also be provided in the manuscript.

Flow Cytometry

Plots

Confirm that:

☒ The axis labels state the marker and fluorochrome used (e.g. CD4-FITC).

☒ The axis scales are clearly visible. Include numbers along axes only for bottom left plot of group (a 'group' is an analysis of identical markers).

☒ All plots are contour plots with outliers or pseudocolor plots.

☒ A numerical value for number of cells or percentage (with statistics) is provided.

Methodology

Sample preparation	Cells in culture were trypsinised, washed, filtered through a 70um strainer and stained with the indicated antibodies for 20-30 minutes on ice. DAPI was added to discriminate dead cells just prior to running on the cytometer.
Instrument	BC FACS CANTO II, BD LSR Fortessa
Software	BD FACSDIVA 8.0.1
Cell population abundance	Cell populations were analyses based on fluoresence intensity and not population size.
Gating strategy	Cells were intitially gates based on FSC-A vs SSC-A, followed by FSC-A vs FSC-H to obtain single cells. Dead cells were excluded based on DAPI fluoresence. Where appropriate, the median fluoresence intensity (MFI) was calulated for the indicated channels.

☒ Tick this box to confirm that a figure exemplifying the gating strategy is provided in the Supplementary Information.
