# Supplementary material for: DNA barcoding reveals ongoing immunoediting of clonal cancer populations during metastatic progression and immunotherapy response
Source: Nat Commun. 2022 Nov 7;13:6539. doi: 10.1038/s41467-022-34041-x (PMC9640547; doi:10.1038/s41467-022-34041-x)
Supplement: Supplementary file 13 — Supplementary tables [file 41467_2022_34041_MOESM13_ESM.docx]

Supplementary table 1. Barcode overlap between tow cell pellets from independent barcoding experiments using the 4T1 cell line.

Supplementary table 2: DNA barcode insertion sites of IE1 and IE2.
