# Supplementary material for: DNA barcoding reveals ongoing immunoediting of clonal cancer populations during metastatic progression and immunotherapy response
Source: Nat Commun. 2022 Nov 7;13:6539. doi: 10.1038/s41467-022-34041-x (PMC9640547; doi:10.1038/s41467-022-34041-x)
Supplement: Supplementary file 2 — Peer Review File [file 41467_2022_34041_MOESM2_ESM.pdf]

nature portfolio

Peer Review File

DNA barcoding reveals ongoing immunoediting of clonal cancer populations during metastatic progression and immunotherapy response

Open Access This file is licensed under a Creative Commons Attribution 4.0 International License, which permits use, sharing, adaptation, distribution and reproduction in any medium or format, as long as you give appropriate credit to the original author(s) and the source, provide a link to the Creative Commons license, and indicate if changes were made. In the cases where the authors are anonymous, such as is the case for the reports of anonymous peer reviewers, author attribution should be to 'Anonymous Referee' followed by a clear attribution to the source work. The images or other third party material in this file are included in the article's Creative Commons license, unless indicated otherwise in a credit line to the material. If material is not included in the article's Creative Commons license and your intended use is not permitted by statutory regulation or exceeds the permitted use, you will need to obtain permission directly from the copyright holder. To view a copy of this license, visit http://creativecommons.org/licenses/by/4.0/.

Reviewers' Comments:

Reviewer #1:
Remarks to the Author:
The study presented by Baldwin and colleagues aims to discern between two predominant mechanisms of immunoediting in a genetically engineered mouse mammary tumor model: loss of strongly immunogenic populations vs. expansion/selection of populations with less immunogenic features. This is a truly fascinating study, aimed at addressing an interesting and clinically important question in breast cancer. The study utilizes state of the art technologies and treatments to address a novel question. Overall, some additional barcode data analysis and immune population analyses will benefit this study as described below.
• Introduction/discussion
o please provide commentary on how results from this study compare to those observed in other breast cancer xenograft studies, particularly with respect to clonal dynamics of metastasis and clonal dynamics of cytotoxic chemotherapy response (e.g. PMID 30498242, PMID 30996079, PMID 31019194)
o In patients, how does clonal diversity (estimated from genomic sequencing) correlate with ICT responses?
• Please state the complexity of the ClonTracer barcode library
• Supplementary data needed for figures 1 and 2 (this is needed for 4T1 and EMT6 cells):
o FACS plots should be provided for the initial isolation of the 10% infection efficiency barcode transduction of the 4T1 and EMT6 cells
o precise description of for how many population doublings / passages cells were grown between FACS sorting and mammary implantation?
o Please provide NGS data demonstrating that barcode distribution is maintained during passaging in culture after FACS (is there drift in the population)? Data needs to be provided demonstrating that barcode distribution is equal in each mouse that was engrafted (in other words, after passaging X passages following FACS, then splitting the barcoded population between mice, can authors demonstrate that the same barcode repertoire was truly engrafted in each mouse?) – please see PMID 30726735
o What is the tumor initiating cell frequency (ie number of barcodes detected in primary tumors / number of barcodes engrafted into a single mouse)
• From the methods, it seems that different cell numbers were engrafted in WT vs NSG mice, yet tumor growth rates are compared in Fig 2B. Please clarify how many cells were engrafted. If different cell numbers were engrafted WT vs NSG, growth rates cannot be compared.
• Line 89-91 – please edit this statement to instead say 'These results suggest…' . These are different mice strains and there could be other indirect effects impacting tumor growth rate, assuming the same cell numbers were implanted.
• Fig 2d – please provide tumor growth curves in individual format, as only 50% of mice had a relapse as stated in the text and this is not discernable from the averaged data that is plotted.
o Do the mice with completely regressed tumors harbor metastatic lesions at day 60?
• Please state in the text, and indicate diagrammatically in Figure 2, at what timepoint control/ICT-treated tumors were harvested for barcode analysis presented in figure 2f-h)
• Line 116- please change 'indicates' to 'suggests'
• Fig 2 – additional barcoding data needs to be provided:
o please provide NGS data revealing barcode numbers, Shannon diversity, and distribution in barcoded cells prior to mouse implantation.
o Please provide analysis of how barcode distributions compare between individual mice within each treatment group. Since this was a cultured cell line and the goal was to implant barcodes with '6 fold over-representation' please provide evidence this was achieved
o Fig 2H – please clarify whether the 5 barcodes presented are the only barcodes overlapping between those mice. If not, please provide comprehensive analysis of how many barcodes (what percentage?) were overlapping between mice. How were the barcodes presented in H selected for this plot? A different color scheme might be beneficial- as-is, most barcodes look to be at a similar frequency in

ICT-treated tumors as in the control groups
• 4T1 experiment/ Figure 3 – please provide commentary about why barcode patterns in WT vs NSG were similar with 4T1 cells, but not with EMT6 cells. What inherent differences in these cell lines could drive this?
o Please provide quantitative data of lung metastatic burden in WT mice with control or ICT treatment (if fluor/luminescent cells were used, imaging data can be quantified. If not, metastasis area quantified from histology images would suffice, or at a minimum lung weight could be used as a rough estimate)
o This is not necessary, but if authors could at least discuss or even provide data it would be beneficial – at day 15 when tumors are resected, have metastases already seeded the lungs? Please discuss whether you think the ICT treatments are targeting cells in circulation, or rather micro-metastatic lesions on day ~15.
• Line 154- please change indicates to suggests
• Please provide data (e.g. flow cytometry) demonstrating depletion of CD8 T cells or NK cells using the anti-CD8 and anti-asialo-GM1. Please also provide lung met burden data, as suggested above, for this experiment.
• Line 190- please further explain how the results agree with the Wagenblast et al findings.
• If possible, can the colors used in 4c-d be also labeled with the clones in other parts of fig 4?
• For all barcoding data, it is critical that distribution plots be provided. Often times, simply counting number of unique barcodes does not paint a complete picture- several xenograft studies have demonstrated that many clones can be maintained in extremely low abundance. Please provide distribution plots and description in the text of how abundant/low-abundance clones are after ICT and in lung mets versus control primary tumors. Particularly the clones of focus in figure 4 – are these maintained in high copy number? Are all of those predominant clones maintained at similar levels or not?
• Figure 5- please provide a venn diagram, or similar, demonstrating the overlap (or lack thereof) in differentially expressed genes between IE1 and IE2.
• The gene expression analysis in figure 5 would be strengthened by bulk RNA seq analysis of ICT-treated versus control tumors. This would clarify interpretations of the individual clone transcriptomic analysis. This is true especially in light of the fact that recovering these clones from culture could greatly impact their RNA profiles.
• Authors should provide PDL1 IF/IHC staining of their control and ICT-treated tumors. We would expect to see subclonal PDL1 expression based on the clonal transcriptome analysis.
o Ideally, co-staining on tissue showing that PDL1 expression is present on a distinct tumor cell population from the MHC-loss population, would be quite compelling to demonstrate that two unique clonal lineages have unique immune evasion mechanisms and these unique mechanisms are maintained together within the same tumor.
• Authors should mine their RNA seq to search for epigenetic regulators that may impact MHC transcriptional regulation
• Authors should measure TILs and NK cells in their control and ICT treated tumors. How are levels of these immune populations expected to correlate with clonal diversity? Is there a correlation when comparing the two cell lines?

Reviewer #2:
Remarks to the Author:
Baldwin et al utilized DNA barcoding to track clonal dynamics during primary tumor and metastatic outgrowth of two mouse cell lines within immunocompetent and immunocompromised mice. They also tracked clonal dynamics after administration of immune checkpoint blockade (anti-PD1 & anti-CTLA-4). Considering the relatively low response rates of immune checkpoint blockade (ICB) in breast cancer, mechanistic characterization of ICB response and escape would be a novel contribution to the field.
Clonal selection due to immunoediting is a widely accepted model of tumor evolution and several

longitudinal studies characterizing primary and metastatic patient tumor tissues exemplify these principles. While significant mechanistic insight can be derived from in vivo modeling, there is relatively limited molecular and functional characterization of the models presented, which ultimately detracts from the novelty of this study. Thus, the authors essentially just confirmed prior data (much of it in patients) in their mouse model without giving any novel insights beyond what's known. Overall, the authors would need to greatly expand the breadth and depth of their molecular and functional characterization in order to increase the novelty of their study. It would be useful of that the authors augment their introduction, as there is a substantial body of relevant literature that is not referenced, that the authors correct all typos found in the manuscript, and importantly, that the statistics used throughout the data are re-evaluated for appropriateness (repeated t-test is not appropriate for comparing several groups, ANOVA should be used instead).

The data presented in Baldwin et al support the author's conclusions that clonal selection occurs during primary tumor expansion and metastatic outgrowth and this is augmented by immunotherapy. However, the authors only show a decrease in clonality and do not actually prove that the eliminated clones were immunologically different leading to their elimination.

The authors used transplantable mouse mammary tumor cell lines for their studies that grew extremely fast. The use of transplantable models is not the best choice for immunological studies, since the injected tumors do not have an endogenous immune environment and establishing this takes time.

The authors performed bulk RNA-seq of two clones that were enriched after ICB as a way to prove immune editing. However, the usefulness of this data is not immediately apparent for several reasons. Firstly, of the post-ICB expanded clones, only two were pursued for follow-up analyses. It is not clear how the authors selected these particular clones for characterization, but this approach is limited in scope and ignores the contributions from other potentially important clones, as well as information on clonal dynamics/interaction. Secondly, aside from the initial in vivo experiments being initiated from mouse cell lines, the two clones that were selected for molecular characterization were subjected to further selection in vitro via culturing and freezing to establish cell lines prior to RNA-seq. While this is perhaps an unavoidable aspect of the experimental design, these limitations along with the limited scope of characterization challenge the applicability of the findings to the patient gene expression data (particularly since there is limited mechanistic follow-up). Finally, the differential gene expression of the two clones showed little overlap, confirming the phenotypic/functional heterogeneity at play, and of these genes, the authors only performed limited follow-up experiments on MHC expression. Downregulation of MHC expression is also a well-documented feature of immune escape, so the authors utilized a demethylating agent (5-aza) and Interferon-gamma treatment in an attempt to rescue MHC levels. The authors conclude that gene hypermethylation is not the mechanism of MHC-I suppression in the IE1 clone, however, in supplemental figure 6A, 200nM 5-aza seems to increase MHC-I expression to parental control levels. Additionally, there is no in vitro or in vivo functional data to demonstrate the relevance of the decreased or increased MHC levels.

Reviewer #3:
Remarks to the Author:
In this study, Baldwin LA. et al. use DNA barcoding of immunotherapy-sensitive mouse mammary carcinoma EMT6 cells and the metastatic 4T1 mammary carcinoma mouse line to track clones of tumor cells during tumor outgrowth and/or metastasis in immunodeficient mice [NOD SCID Gamma (NSG)], immune-competent mice (syngeneic WT mice) or immune-competent mice treated with immune checkpoint therapy. As expected, primary EMT6 tumors grew faster in immunodeficient NSG mice as compared to WT mice. Treatment of EMT6 bearing WT mice with anti-PD-1 and anti-CTLA-4 immune checkpoint therapy induced tumor regression, with about 50% of mice subsequently experiencing tumor relapse and outgrowth. The researchers then examined the number and

distribution of barcodes present in the tumors at endpoint and found tumors grown in NSG mice had 50 times the number of unique barcodes as those grown in WT mice, which in turn had more than 20 times the number of unique barcodes as those tumors from WT mice treated with immunotherapy. Shannon diversity analysis revealed a trend to a lower barcode diversity in EMT6 tumors from WT mice than that in tumors from immunodeficient NSG mice with significantly lower diversity present in tumors from WT mice treated with immune checkpoint therapy.

The researchers then used DNA barcoded 4T1 cells orthotopically injected into the mammary fat pad (and subsequently surgically resection of primary tumors to allow metastasis to the lungs) of WT and NGS mice to determine whether immunoediting occurred during metastasis, as measured by enrichment or depletion of specific tumor clones in metastatic lung tumors.
Primary tumor sizes between WT mice and NSG mice were similar at the time of resection. However, NSG mice succumbed to metastatic disease earlier than WT mice. Anti-PD-1 and anti-CTLA-4 immune checkpoint therapy led to a small but statically significant increase in survival compared to WT mice. Analysis of barcode diversity revealed similar numbers of primary tumor clones and barcode diversity between NSG and WT hosts. In contrast, the lung metastases of NSG mice contained significantly more barcode clones than lung mets from WT control mice. A 70% reduction (compared to control WT mice) in the number of clones in metastatic tumors in WT mice treated with anti-PD-1 and anti-CTLA-4 immune checkpoint therapy was observed. The higher barcode number in the lung mets of NSG mice compared to WT mice was associated with a higher diversity of barcode as measured using the Shannon diversity index. A significant reduction in barcode diversity was observed in WT mice treated with anti-PD-1 and anti-CTLA-4 immune checkpoint therapy. The authors then treated WT mice starting one day before primary tumor resection with anti-CD8 or anti-asialo-GM1 to deplete anti-CD8 or NK cells, respectively. Neither antibody depletion lead to a significant difference in mouse survival. Depletion of CD8+ T cells led to an increase in the number of clones detected within the lungs.

Unsupervised hierarchical clustering revealed that primary tumors cluster together, irrespective of the immune status of the mouse (WT or NSG), whereas lung tumors formed in the NSG mcie did not cluster with lung tumors formed in WT mice. Specific barcodes were enriched or depleted in across replicate mice, with three barcodes that were enriched in NSG lung mets present at lower abundance in non-treated WT mice and completely absent with anti-PD-1 and anti-CTLA-4 treatment. Several metastatic clones that were present in the lung of NSG and WT mice were enriched following immunotherapy.

They then established two “resistant” clonal cell populations (from the parental barcoded 4T1 cell population in vitro) called 1E1 and 1E2 and two independent control clones (NT1 and NT2) that were not enriched following immunotherapy. All clonal cell lines had similar in vitro growth kinetics. Transcriptomic analysis of the clonal cell lines revealed the IE1 clone had 1553 differentially expressed genes whereas the IE2 clone had 1099 differentially expressed genes (compared to bulk 4T1 cells). Comparison of the top differentially expressed genes (as compared to parental 4T1 cells) between the IE1 and IE2 clones did not reveal any with known role in immune evasion. GSEA revealed primarily different pathways between 1E1 and 1E2. The top downregulated gene-set for IE1 was “REACTOME_UB_SPECIFIC_PROCESSING_PROTEASES” that contained two genes involved in antigen processing for display by MHC class I (Psmb8 and Psmb9). Further analysis revealed the IE1 clone had significantly reduced expression of multiple genes related to antigen presentation, including H2-k1, Tap2, Psmb8, Psmb9 and Psmb10, of which H2-k1 expression was validated at the protein level using flow cytometry. The IE2 cells had a significantly increased expression of Cd274 (PD-L1) that was validated at the protein level.
Treatment of the clonal cell lines with the demethylating agent 5-aza did not fully restore MHC expression in the IE1 clone. The IE1 clone responded to IFN-gamma treatment and upregulated MHC-I expression but at lower levels compared to the parental 4T1 cells. Finally, analysis of gene signatures in human breast cancer patients revealed the 1E1 and 1E2 overlapping gene signature was associated with poor survival in breast cancer patients.

Overall, this is a solid study that is of interest. Immunoediting of primary and metastatic tumors over time has broad implications for therapy and understanding this is of significant importance. However, there are some things that should be addressed before I can recommend publication in Nature Communications.

While the authors demonstrate nicely that unmanipulated and/or immunotherapy-induced immunity can edit primary and metastatic tumors, the mechanism by which this happens is unclear and mostly inferred.

Specific comments:

- NSG mice lack CD4+ T cells, CD8+ T cells, Tregs, B cells, and functional NK cells and immune checkpoint therapy often requires both CD4+ T cells and CD8+ T cells, with NK cells and even B cells possibly contributing. Depleting just CD8+ T cells or NK cells is a pretty limited approach towards discovering the mechanism by which immunoselection of specific tumor clones in occurring. In fact, depletion of either CD8+ T cells or NK cells in WT mice had no effect on survival in the metastatic setting, and only CD8+ T cell depletion led to a significant, but small increase in the number of clones in the lungs. As far as I could tell, the authors do not even mention CD4+ T cells when these have been shown to be almost or as important as CD8+ T cells during cancer immunoediting and immunotherapy responses.

- While the 1E1 clone has reduced MHC-I expression and the 1E2 clone has increased PD-L1 expression, no relevant assays to determine whether these observations are relevant are performed. Antigen presentation assays to T cells, enforced over and under-expression of MHC-I and PD-L1, and characterization of in vivo growth (of the clones upon manipulation) has not been examined. Not necessarily recommending those specific assays, but these types of experiments would strengthen the connection to the transcriptomic and GSEA analysis.

- To the previous point, do clones 1E1 and 1E2 form primary tumors in WT and NSG mice and do they metastasize compared to the NT1 or NT2 clones?

Minor points
- Please check Figure 2D. I am not sure it is appropriate to plot average tumor volume of individual mice once a mouse has been removed from the group due to reaching volume endpoint. In other words, past day 45 or so it appears that some mice (in the anti-PD-1/anti-CTLA-4 group) were euthanized and therefore, removed from the time point on the plot (which will skew the average volume and might be misleading). Maybe showing plots with each individual mouse might be more appropriate if the goal is to show some mice experience tumor recurrence.

- Line 84: For a more general audience, I would mention what components of the immune system are missing in NSG mice. For example, "NSG mice lack T cells, B cells, and the IL-2 receptor common gamma chain, whereby the absence of the latter renders NK cells functionally deficient".

- Line 115: Data is plural so it should be "These data", as opposed to "This data".

POINT BY POINT RESPONSES TO REVIEWERS:

Reviewer #1:

1.1 Please provide commentary on how results from this study compare to those observed in other breast cancer xenograft studies, particularly with respect to clonal dynamics of metastasis and clonal dynamics of cytotoxic chemotherapy response (e.g. PMID 30498242 , PMID 30996079, PMID 31019194)

Response: We agree with the reviewer's comment that this will improve the manuscript.

Revisions: The following text was added to the discussion, beginning at line 478: "A number of previous barcoding studies in breast cancer had focused on metastasis and response to chemotherapy. However, these were performed in immunocompromised mice so that the role of the immune system in these processes was not addressed (*1-4*). In the absence of a fully intact immune system it was demonstrated that specific clones have greater metastatic ability (*1, 3, 4*). Our results suggest that a subset of these highly metastatic clones identified in these studies may have been recognised by an intact immune system and removed via immunoediting. Similar to our results, these studies found that the dominant clone within the primary tumour generally was not the dominant clone in the metastases (*1, 3, 4*). Some of these studies also showed that chemotherapy treatment of PDX models led to a decrease in clonal abundance and diversity in relapsed disease, which is similar to what we found with immunotherapy (*2, 3*). Future studies combining immunotherapy with chemotherapy utilising a similar regimen to the atezolizumab plus nab-paclictaxel of the Impassion130 trial would give important insights into how combining these two treatment modalities affected clonal diversity following relapse (*5*)."

1.2 In patients, how does clonal diversity (estimated from genomic sequencing) correlate with ICT responses?

Response: We think this is a very interesting question. After searching the literature, we were unable to find any relevant studies carried out in patients treated with immune checkpoint inhibitors that correlated clonal diversity to response. We welcome any specific suggestions. Related work in this area has been carried out in non-small cell lung cancer, where loss of HLA has been associated with increased intratumoural heterogeneity (*6*). Furthermore, ITH has been shown to increase after chemotherapy in small cell lung cancer and is associated with treatment relapse (*7*). Although interesting, we feel these papers fall beyond the scope of this study.

1.3 Please state the complexity of the ClonTracer barcode library.

Response: We have added this detail to the manuscript.

Revision: We have added the following text to the results at line XX: "that contains approximately 7 million unique barcodes".

1.4 Supplementary data needed for figures 1 and 2 (this is needed for 4T1 and EMT6 cells): FACS plots should be provided for the initial isolation of the 10% infection efficiency barcode transduction of the 4T1 and EMT6 cells.

Response: We agree with the reviewer that this will improve the manuscript and have added supplementary figures and text with this data.

Revision: We have added this data to supplementary figure 1 and supplementary figure 3. This is referenced in the text at lines 86 and 138.

1.5 Please provide a precise description of for how many population doublings / passages cells were grown between FACS sorting and mammary implantation?

Response: We have added supplementary figures and text with this data.

Revision: The following text was added to the method at line 625: "The EMT6 cells and the high complexity 4T1 cells were passaged twice following cell sorting, frozen and cells from these aliquots were thawed and passaged once more prior to transplantation. The low complexity 4T1 cells (4T1 BC5000) were a subpool derived from the higher complexity 4T1 cell line, these were passaged an additional 3 times to expand and freeze and were then thawed and used as described above."

1.6 Please provide NGS data demonstrating that barcode distribution is maintained during passaging in culture after FACS (is there drift in the population)? Data needs to be provided demonstrating that barcode distribution is equal in each mouse that was engrafted (in other words, after passaging X passages following FACS, then splitting the barcoded population between mice, can authors demonstrate that the same barcode repertoire was truly engrafted in each mouse?) – please see PMID 30726735.

Response: We have now added Shannon diversity data for the cell pellet that demonstrates that the clonal diversity in the cell pellet is very similar to that which is engrafted into the primary tumours of NSG mice. Furthermore, we have analysed the cell pellet barcode overlap of two independent experiments. The content of the cell pellets was >95% identical for a cut-off of 5 barcode reads, or >97% identical for a cut-off of 10 reads. Overall, we feel this confidently demonstrates that clonal diversity does not change from experiment to experiment and that the clonal diversity seen within the cell pellet is maintained.
We did not perform analysis of barcode drift during passage so cannot provide this information. The high degree of hierarchical clustering between all of the primary tumours in figure 4 that spans two independent experiments indicates that a similar barcode repertoire was engrafted into each mouse.

Revision: We have included data outlining the overlap between cell pellets in supplementary table 1.

1.7 What is the tumor initiating cell frequency (i.e. number of barcodes detected in primary tumors / number of barcodes engrafted into a single mouse)?

Response: We agree that this is an interesting question although it is not one that these experiments were designed to answer. However, from the high complexity 4T1 data we can see that ~12,000 barcodes were detected following injection of 50,000 cells with a total complexity of ~300,000 barcodes. This indicates approximately 1:4 cells engrafted. In contrast in the 4T1 model, 5,000 barcode cells and the EMT6 cells had too few barcodes present to estimate engraftment rates as there was 5-10 times less barcodes present as cells injected.

1.8 From the methods, it seems that different cell numbers were engrafted in WT vs NSG mice, yet tumor growth rates are compared in Fig 2B. Please clarify how many cells were engrafted. If different cell numbers were engrafted WT vs NSG, growth rates cannot be compared.

Response: We apologise if this was unclear, but direct the reviewer to the results, line 87and the methods line 641. We can confirm the same number of cells were injected into WT and NSG mice for each model. For the 4T1 model this was 50 000 cells per mouse and for the EMT6 model this was 250 000 cells per mouse.

1.9 Line 89-91 – please edit this statement to instead say 'These results suggest…'. These are different mice strains and there could be other indirect effects impacting tumor growth rate, assuming the same cell numbers were implanted.

Response: We agree that this improves the paper and have updated the results.

Revision: Line 96 was edited to say "These results suggest…"

1.10 Fig 2d – please provide tumor growth curves in individual format, as only 50% of mice had a relapse as stated in the text and this is not discernable from the averaged data that is plotted.

Response: We felt that the combination of the tumour growth and KM plot allowed for the reader to determine how relapse influenced tumour growth. To address the reviewer's request we have now updated the figure to include individual tumour growth curves.

Revision: Figure 2D has been updated with individual tumour growth curves.

1.11 Do the mice with completely regressed tumors harbor metastatic lesions at day 60?

Response: We thank the reviewer for pointing out this omission, we have since updated the wording of the results section to address this.

Revision: The following sentence was added to the results at lines 106-108: "During harvest a small residual lesion was observed in two of these mice, no metastatic lesions were observed in any of the mice irrespective of treatment."

1.12 Please state in the text, and indicate diagrammatically in Figure 2, at what timepoint control/ICT-treated tumors were harvested for barcode analysis presented in figure 2f-h).

Response: Mice were harvested at ethical endpoint for barcode rather than at specific timepoints. We feel this addition would cause the figure to be confusing and difficult to interpret.

Revision: Added this to the results at lines 112-113: "collected at ethical endpoint in the experiment described above"

1.13 Line 116- please change 'indicates' to 'suggests'.

Response: We agree that this will improve the accuracy of the paper and have updated the results.

Revision: We have updated line 125 in the results from 'indicates' to 'suggests'.

1.14 Fig 2 – additional barcoding data needs to be provided. Please provide NGS data revealing barcode numbers, Shannon diversity, and distribution in barcoded cells prior to mouse implantation.

Response: We agree that this data strengthens the manuscript. We have now generated and included NGS data demonstrating unique barcode counts and Shannon diversity. We have also provided barcoding data for the remaining cell pellet as a proxy for barcode diversity prior to implantation.

Revision: Inclusion of supplementary figure 2, panels A-D. We have provided barcoding data for the left over cell pellet after implantation as a proxy for barcode diversity prior to implantation.

1.15 Please provide analysis of how barcode distributions compare between individual mice within each treatment group. Since this was a cultured cell line and the goal was to implant barcodes with '6 fold over-representation' please provide evidence this was achieved.

Response: We have carried out analyses of barcoding data and provided distribution plots for individual mice as well as Shannon diversity. In supplementary figure 2A, we show that more than 40,000 unique barcodes were detected in the remaining cell pellet. Given 250,000 cells were transplanted, we feel confident 6-fold over representation of the barcode library was achieved.

Revision: We direct the reviewer to the additional information provided in supplementary figure 2.

1.16 Fig 2H – please clarify whether the 5 barcodes presented are the only barcodes overlapping between those mice. If not, please provide comprehensive analysis of how many barcodes (what percentage?) were overlapping between mice. How were the barcodes presented in H selected for this plot? A different color scheme might be beneficial- as-is, most barcodes look to be at a similar frequency in ICT-treated tumors as in the control groups.

Response: We agree clarification is required.

Revision: Inclusion of supplementary figure 2, panel D. The upset plot shows the barcodes represented in figure 2H as the only barcodes common across treatment groups.

1.17 4T1 experiment/ Figure 3 – please provide commentary about why barcode patterns in WT vs NSG were similar with 4T1 cells, but not with EMT6 cells. What inherent differences in these cell lines could drive this?

Response: We can offer only speculation as to this difference in phenotype. To us, this suggests the vast majority of 4T1 cells are inherently resistant to immune control. Alternatively, 4T1s may have the ability to rapidly establish a protumourigenic and immune suppressive microenvironment after implantation. Further experiments would be required to fully elucidate this.

Revision: We have added the below to the discussion at line 459:

"Intriguingly the 4T1 model unlike the EMT6 model showed little immunoediting in the primary tumour. This suggests that either the vast majority of 4T1 cells are inherently resistant to immune control at the orthotopic site, or that 4T1 cells very rapidly set up a suppressive immune microenvironment that protects the majority of clones from immune mediated killing. The ability of 4T1 cells to induce myeloid derived suppressor cells could well contribute to a suppressive immune microenvironment, however, further studies would be necessary to further clarify this (10.1186/s13058-019-1189-x)."

1.18 Please provide quantitative data of lung metastatic burden in WT mice with control or ICT treatment (if fluor/luminescent cells were used, imaging data can be quantified. If not, metastasis area quantified from histology images would suffice, or at a minimum lung weight could be used as a rough estimate).

Response: Lung samples were collected at ethical endpoint when metastatic burden was broadly equivalent between groups, based on clinical measurements and lung weight. Lung samples were processed for DNA extraction and barcode analysis so no histology could be performed.

1.19 This is not necessary, but if authors could at least discuss or even provide data it would be beneficial – at day 15 when tumors are resected, have metastases already seeded the lungs? Please discuss whether you think the ICT treatments are targeting cells in circulation, or rather micro-metastatic lesions on day ~15.

Response: We welcome the opportunity for further discussion and have added text to the manuscript.

Revision: We have added the following text to the discussion at line 467:
"As we wanted to understand the role of immunotherapy on controlling metastatic disease we examined lung metastases from the 4T1 model following resection of the primary tumour. Lung metastasis occurs early in the 4T1 model with our studies indicating metastases can form following resection as early as day 13 (data not shown), and others showing the related 4T1.2 model robustly metastasizes by day 10 (*8*). This indicates that when adjuvant immunotherapy was given, these therapies were activating immune cells to target micrometastases that had already formed within the lungs. We postulate that while possible it is unlikely that circulating cancer cells were a major target of adjuvant immunotherapy as previous studies have indicated that circulating breast cancer cells only have a short half-life of 1-2.4 hours in circulation (*9*)"

1.20: Line 154- please change indicates to suggests

Response: We agree this strengthens the manuscript.

Revision: We have changed the text at line 164 from "This indicates…" to "This suggests…"

1.21 Please provide data (e.g. flow cytometry) demonstrating depletion of CD8 T cells or NK cells using the anti-CD8 and anti-asialo-GM1. Please also provide lung met burden data, as suggested above, for this experiment.

Response: We agree inclusion of flow cytometry data strengthens the manuscript. We have now generated and included this data.
For these experiments, mice were harvested at ethical endpoint which includes respiratory distress and rapid breathing, indicative of high lung metastatic burden. Providing lung weight (as a proxy for lung metastatic burden) would not be informative as mice are harvested at end stage where the lungs are similarly overrun with metastases between treatment groups.

Revision: To address the comment, we carried out a flow cytometry experiment to confirm depletion of target cell types. This data is included in supplementary figure 6 B-D, and provided the gating strategy in supplementary figure 7.

1.22 Line 190- please further explain how the results agree with the Wagenblast et al findings.

Response: We have clarified our stance by adding text, as below.

Revision: Further elaborated and changed text at line 209 from "This agrees with the findings of Wagenblast and colleagues (23)." To "This is similar to the findings of Wagenblast and colleagues who examined 4T1 clonal diversity in metastases in NSG (but not wild type mice) and found tissue-specific enrichment of unique barcode clones."

1.23 If possible, can the colors used in 4c-d be also labelled with the clones in other parts of fig 4?

Response: Figure 4 panels C and D were generated by plotting the top 9 barcodes present in the lungs of either NSG, Balb/c, control treated and immunotherapy treated mice. These are not necessarily the same barcodes as represented in panel B. To assist interpretation of this figure, we have altered the colour of panel C (reflecting the different barcode clones displayed compared to panel D), and have added labels for the clones where possible.

Revision: Figure 4C, D colour change. Addition of labels.

1.24 For all barcoding data, it is critical that distribution plots be provided. Often times, simply counting number of unique barcodes does not paint a complete picture- several xenograft studies have demonstrated that many clones can be maintained in extremely low abundance. Please provide distribution plots and description in the text of how abundant/low-abundance clones are after ICT and in lung mets versus control primary tumors. Particularly the clones of focus in figure 4 – are these maintained in high copy number? Are all of those predominant clones maintained at similar levels or not?

Response: We agree that this data strengthens the manuscript and have now generated and included this data.

Revision: We have now included this data in supplementary figure 4, panels A-D. Panels A and B show the dramatic reduction in unique barcodes present in the lungs compared to the primary tumours, further exacerbated by treatment with immunotherapy. Distribution plots are provided in panels C and D.

1.25 Figure 5- please provide a venn diagram, or similar, demonstrating the overlap (or lack thereof) in differentially expressed genes between IE1 and IE2.

Response: We direct the reviewer to figure 7a for a Venn diagram which includes both IE1 and IE2, as well as NT1 and NT2.

1.26 The gene expression analysis in figure 5 would be strengthened by bulk RNA seq analysis of ICT-treated versus control tumors. This would clarify interpretations of the individual clone transcriptomic analysis. This is true especially in light of the fact that recovering these clones from culture could greatly impact their RNA profiles.

Response: We thank the reviewer for their comment but respectfully disagree and question the utility of bulk RNAseq in this setting as we consider it unlikely to be interpretable. Our reasoning is as follows:

1. We identified two clones that were reproducibly enriched in lung metastases of mice following immunotherapy. This strongly indicated that these clones had a pre-existing immunotherapy resistance phenotype.
2. These two clones were then prospectively isolated from the starting pool of cells to determine what pre-existing gene expression features they had that could explain their immunotherapy resistance phenotype. These two clones each had distinct as well as overlapping gene expression features when compared to the bulk population and other isolated clones.
3. Whilst *enriched*, these clones vary in abundance and are mixed with ~20 other clones that vary from mouse to mouse in the lung metastases that we analysed. Thus, any bulk sequencing will most likely recapitulate the bulk 4T1 population rather than the interesting clones and not be informative. In particular we do not believe this would clarify interpretations of the individual clones.
4. The method of clonally selecting clones of interest followed by molecular profiling is established in the field and has been used previously (Eg in Wagenblast et al, Nature)

1.27 Authors should provide PDL1 IF/IHC staining of their control and ICT-treated tumors. We would expect to see subclonal PDL1 expression based on the clonal transcriptome analysis. Ideally, co-staining on tissue showing that PDL1 expression is present on a distinct tumor cell population from the MHC-loss population, would be quite compelling to demonstrate that two unique clonal lineages have unique immune evasion mechanisms and these unique mechanisms are maintained together within the same tumor.

Response: We agree this will strengthen the manuscript. PD-L1 was undetectable by IHC so we performed flow cytometry on end-stage immunotherapy treated metastatic lungs to address the reviewer's query. We show there are distinct populations of cancer epithelial cells present with varying expression of MHC I and PDL1. We observe an MHC I-high population with varying expression of PDL1. Additionally, we also see a MHC I-low population with varying PDL1 expression. This provides compelling evidence of multiple known mechanisms of immune evasion occurring and being maintained within the same tumour

Revision: Addition of flow cytometry data in supplementary figure 11. Addition of the following text to the manuscript at line 312

"To determine if these two mechanisms are simultaneously maintained *in vivo*, we carried out flow cytometry for MHC-I and PD-L1 on advanced 4T1 lung metastases from immunotherapy treated BALB/c mice. By isolating RFP+ cancer epithelial cells from lung tissue, we observed MHC-I-high and MHC-I-low neoplastic populations, both with varying expression of PD-L1 (Sup Fig 11). This provides convincing

evidence of these two mechanisms of immune evasion being maintained simultaneously *in vivo*."

1.28 Authors should mine their RNA seq to search for epigenetic regulators that may impact MHC transcriptional regulation.

Response: We find the epigenetic regulation of MHC I expression intriguing. In response to this request, further GSEA and Cytoscape analysis of our derived gene signature identified three interrelated negatively-enriched gene sets, "BENPORATH_ES_WITH_H2K27ME3", "BENPORATH_PRC2_TARGETS" and "BENPORATH_SUZ_12_TARGETS". Ben-Porath and colleagues found these signatures correlated with a stem-like phenotype in patient breast cancer datasets and associated with poor prognoses. Further investigation showed a number of the top genes in these gene sets are also present in our derived signature and are targets of DNA methylating complex PRC2, namely HHIP, CWH43, WNT10B, ABC3, CHN2 and CRIP1. PRC2 is known to regulate MHC I expression. While we only see subtle changes in PRC2 expression in our RNAseq, we do see far greater downregulation of the targets of PRC2, perhaps suggesting an over-active PRC2 complex. It is important to note that these observations were made by analysing the gene signature derived from the common DEGs from both IE1 and IE2. It is unlikely that inhibition of PRC2 in itself will result in the complete rescue of MHC I expression in IE1 to control or IE2 levels. We find the epigenetic drivers of immune evasion intriguing, but believe substantial further investigation would require extensive work that is beyond the scope of this study.

Revision: Addition of text at line 382. "We also identified three gene sets that were closely related and had clear associations with epigenetic regulation, "BENPORATH_ES_WITH_H2K27ME3", "BENPORATH_PRC2_TARGETS" and "BENPORATH_SUZ_12_TARGETS". In patient data, Ben-Porath and colleagues found negative enrichment of these genesets to be correlated with a stem-like phenotype and to be associated with poor prognosis. Further investigation showed the top genes driving these signatures were also negatively enriched in our derived gene signature, namely HHIP, CWH43, WNT10B, ABC3, CHN2 and CRIP1. This suggests a possible role for PRC2-mediated MHC I suppression in our subclones."

1.29 Authors should measure TILs and NK cells in their control and ICT treated tumors. How are levels of these immune populations expected to correlate with clonal diversity? Is there a correlation when comparing the two cell lines?

Response: We find this an interesting question. Unfortunately, we do not have this data as all tissue was collected for barcode analysis. To fully address this question, single cell RNA sequencing with an expressed barcode system and time point analyses would be most appropriate. . Examining the correlation of clonal diversity and TILs would be intriguing, but is ultimately beyond the scope of this study.

Reviewer #2

2.1. It would be useful of that the authors augment their introduction, as there is a substantial body of relevant literature that is not referenced, that the authors correct all typos found in the manuscript, and importantly, that the statistics used throughout the data are re-evaluated for appropriateness (repeated t-test is not appropriate for comparing several groups, ANOVA should be used instead).

Response: We thank the reviewer for their careful consideration of our manuscript. We welcome the reviewer suggesting specific publications for consideration. We have endeavoured to correct all typos within the manuscript. We wish to justify our use of statistical test. In all figure panels (unless otherwise stated), we compare the control condition to that of the experimental group. We do not carry out multiple tests for significance across all groups within a figure panel. The other conditions are plotted to provide context. As we are testing a single hypothesis, we believe the use of unpaired T-tests is justified and appropriate.

2.2 The data presented in Baldwin et al support the author's conclusions that clonal selection occurs during primary tumor expansion and metastatic outgrowth and this is augmented by immunotherapy. However, the authors only show a decrease in clonality and do not actually prove that the eliminated clones were immunologically different leading to their elimination.

Response: We appreciate the reviewer's concerns but we respectfully disagree. We show that certain clones are reproducibly eliminated by the immune system. The fact that they were reproducibly eliminated following immunotherapy but not in NSG mice strongly suggests they are immunologically different. This supports our main claim that immunoediting is active during metastatic progression and in response to immunotherapy.
Finally, we have undertaken new work to assess the capacity for the subclones to suppress T cell activation, both with and without anti-PD1 immunotherapy. This showed that IE2 is capable of directly suppressing T cell activation *in vitro*.

Revision: Inclusion of T cell activation assay data, line 345-365, figure 6

2.3 The authors used transplantable mouse mammary tumor cell lines for their studies that grew extremely fast. The use of transplantable models is not the best choice for immunological studies, since the injected tumors do not have an endogenous immune environment and establishing this takes time.

Response: We acknowledge that cell line models are not perfect. However, as non-transplantable models are not amenable to barcoding studies and transgenic models initiate tumours in all epithelial cells of the mammary gland (leading to multiple tumours), we considered cell line models to be the optimal method for this study. To reflect our understanding of this, we already included the following statement in the discussion about the limitations of cell line models: "A limitation of this study is the reliance on mouse cell line models, which do not recapitulate early stages of tumorigenesis and do not represent the full diversity of human breast cancer.

However, syngeneic allograft models have delivered central insights about the immune response to cancer and demonstrated the utility of immunotherapies (46)."

2.4 It is not clear how the authors selected these particular clones for characterization, but this approach is limited in scope and ignores the contributions from other potentially important clones, as well as information on clonal dynamics/interaction.

Response: We selected the two clones of interest as they were reproducibly enriched across multiple mice and experimental conditions. We direct the reviewer to the manuscript, 222 - 225 .This indicates increased immune evasive abilities compared to the bulk population. We agree with the reviewer that clonal cooperativity is intriguing, however we believe it is beyond the scope of this study.

2.5 Secondly, aside from the initial in vivo experiments being initiated from mouse cell lines, the two clones that were selected for molecular characterization were subjected to further selection in vitro via culturing and freezing to establish cell lines prior to RNA-seq. While this is perhaps an unavoidable aspect of the experimental design, these limitations along with the limited scope of characterization challenge the applicability of the findings to the patient gene expression data (particularly since there is limited mechanistic follow-up).

Response: We agree with the reviewer that there are unavoidable further selective pressures applied to the clones of interest and bear this in mind for all interpretations of data. To help account for these pressures, we also isolated two non-target clones (NT1 and NT2) which act as a control in our studies. Furthermore, the signature we derived clearly demonstrates remarkable prognostic significance in two independent patient data cohorts. This suggests that despite the experimental design we can still identify relevant, novel and important genes involved in human disease. We also note that similar *in vitro* analyses of target clonal cell lines has also been carried out in key papers in the barcoding field (*4*).

2.6 Downregulation of MHC expression is also a well-documented feature of immune escape, so the authors utilized a demethylating agent (5-aza) and Interferon-gamma treatment in an attempt to rescue MHC levels. The authors conclude that gene hypermethylation is not the mechanism of MHC-I suppression in the IE1 clone, however, in supplemental figure 6A, 200nM 5-aza seems to increase MHC-I expression to parental control levels. Additionally, there is no in vitro or in vivo functional data to demonstrate the relevance of the decreased or increased MHC levels.

Response: We appreciate the opportunity to clarify our stance on the role of hypermethylation in the regulation of MHC-I. Treatment of all subclones with 5-aza does increase surface MHC I expression. If hypermethylation was the sole regulator of MHC I expression in IE1, we would expect to see MHC I expression increase not only to control untreated levels, but to match the same treatment condition within the control group. Instead, we only see an increase in MHC I expression to the level of the untreated control. To us, this suggests there are alterative regulatory mechanisms at play.

As the reviewer states, downregulation of MHC expression is a well-documented feature of immune escape. However, we acknowledge that additional in vitro data would strengthen the manuscript. As such, we have undertaken extensive work to examine T cell activation when co-cultured with our clones. This work also addresses a similar query from reviewer 3. This data shows IE2 can suppress T cell activity, even in the context of anti-PD1 immunotherapy.

Revision: Inclusion of T cell activation assay data, line 354 - 365, figure 6

Reviewer #3

3.1 NSG mice lack CD4+ T cells, CD8+ T cells, Tregs, B cells, and functional NK cells and immune checkpoint therapy often requires both CD4+ T cells and CD8+ T cells, with NK cells and even B cells possibly contributing. Depleting just CD8+ T cells or NK cells is a pretty limited approach towards discovering the mechanism by which immunoselection of specific tumor clones in occurring. In fact, depletion of either CD8+ T cells or NK cells in WT mice had no effect on survival in the metastatic setting, and only CD8+ T cell depletion led to a significant, but small increase in the number of clones in the lungs. As far as I could tell, the authors do not even mention CD4+ T cells when these have been shown to be almost or as important as CD8+ T cells during cancer immunoediting and immunotherapy responses.

> **Response:** We agree with the reviewer that depletion of CD4+ cells would strengthen the manuscript. As such, we have undertaken extensive mouse experiments depleting CD4 T cells along with CD8 or NK cells. As previously observed for depletion of CD8+ T cells or NK cells, we found that depletion of CD4 cells had no effect on survival in the metastatic setting. Barcode analysis found that the original observed phenotype was not recapitulated, leading us to conclude that depletion of any one of the cell types from day 14 post surgery does not alter the number of unique barcodes present in the lungs at endstage, suggesting a limited role for these cell types in controlling cancer cell clonal outgrowth and diversity after dissemination. We thank the reviewer for this suggestion that has improved the manuscript.
>
> **Revision:** Inclusion of CD4+ cell depleted mice in survival data in supplementary figure 6. Inclusion of additional barcoding data in supplementary figure 6 E and F. Inclusion of the following text in the manuscript at line 1184-1187:
> "Initial experiments indicated a small change in the number of clones detected within the lung between treatment groups (Sup Fig 6E). However, this was not recapitulated in repeat experiments (Sup Fig 6F). This may suggest that none of these cell types alone are sufficient to restrict clonal diversity after seeding of pulmonary metastases".
>
> Depletion of target cell types was confirmed by flow cytometry (Sup Fig 6B-D, Sup Fig 7).

3.2 While the 1E1 clone has reduced MHC-I expression and the 1E2 clone has increased PD-L1 expression, no relevant assays to determine whether these observations are relevant are performed. Antigen presentation assays to T cells, enforced over and under-expression of MHC-I and PD-L1, and characterization of in vivo growth (of the clones upon manipulation) has not been examined. Not necessarily recommending those specific assays, but these types of experiments would strengthen the connection to the transcriptomic and GSEA analysis.

> **Response:** We agree further insight into the relevance of MHC I and PDL1 expression would strengthen the manuscript. To address this, we have undertaken new work to assess the capacity for the subclones to suppress T cell activation, both with and without anti-PD1 immunotherapy. This showed that IE2 is capable of directly suppressing T cell activation *in vitro*.

Revision: Inclusion of T cell activation assay data, line 345 - 365, figure 6

3.3 To the previous point, do clones 1E1 and 1E2 form primary tumors in WT and NSG mice and do they metastasize compared to the NT1 or NT2 clones?

Response: We have examined the behaviour of the isolated clones in vivo and have now added this data to the manuscript. We found that all clones form primary tumours in wild type mice, and followed similar tumour kinetics to the bulk population. We found that the majority of IE2 tumours established extensive metastases in the lungs (8 out of 12 mice), while some IE1 tumours also metastasised (3 out 10 mice). Neither NT1 or NT2 showed metastatic ability (0 out of 8 mice total).

Revision: Inclusion of primary tumour and metastatic burden data in supplementary figure 9. Inclusion of the following text at line 245
"All clonal cell lines were able to form tumours in the primary setting (Sup Fig 9B). IE2 demonstrated considerable metastatic potential with 66% of mice forming extensive lung metastases (Sup Fig 9C). IE1 also demonstrated some metastatic capacity with 30% of mice forming lung metastases. No mice bearing NT1 or NT2 tumours formed lung metastases (Sup Fig 9C)."

3.4 Please check Figure 2D. I am not sure it is appropriate to plot average tumor volume of individual mice once a mouse has been removed from the group due to reaching volume endpoint. In other words, past day 45 or so it appears that some mice (in the anti-PD-1/anti-CTLA-4 group) were euthanized and therefore, removed from the time point on the plot (which will skew the average volume and might be misleading). Maybe showing plots with each individual mouse might be more appropriate if the goal is to show some mice experience tumor recurrence.

Response: While we originally felt the combination of tumour growth and the KM plot allowed for the reader to determine how relapse influenced tumour growth, we acknowledge that plotting individual tumour growth curves increases clarity and ease of interpretation.

Revision: Figure 2D has been updated with individual tumour growth curves.

3.5 For a more general audience, I would mention what components of the immune system are missing in NSG mice. For example, "NSG mice lack T cells, B cells, and the IL-2 receptor common gamma chain, whereby the absence of the latter renders NK cells functionally deficient".

Response: We agree that this adds to the clarity of the manuscript.

Revision: We have added the following text to the manuscript at line 90: "mice that lack T cells, B cells and functional NK cells"

3.6 Line 115: Data is plural so it should be "These data", as opposed to "This data".

Response: We agree with the reviewer.

Revision: We have made changes as suggested at line 124

References

1. G. V. Echeverria *et al.*, High-resolution clonal mapping of multi-organ metastasis in triple negative breast cancer. *Nature communications* **9**, 1-17 (2018).
2. G. V. Echeverria *et al.*, Resistance to neoadjuvant chemotherapy in triple-negative breast cancer mediated by a reversible drug-tolerant state. *Science translational medicine* **11**, (2019).
3. D. Merino *et al.*, Barcoding reveals complex clonal behavior in patient-derived xenografts of metastatic triple negative breast cancer. *Nature communications* **10**, 1-12 (2019).
4. E. Wagenblast *et al.*, A model of breast cancer heterogeneity reveals vascular mimicry as a driver of metastasis. *Nature* **520**, 358-362 (2015).
5. P. Schmid *et al.*, Atezolizumab plus nab-paclitaxel as first-line treatment for unresectable, locally advanced or metastatic triple-negative breast cancer (IMpassion130): updated efficacy results from a randomised, double-blind, placebo-controlled, phase 3 trial. *The Lancet Oncology* **21**, 44-59 (2020).
6. N. McGranahan *et al.*, Allele-specific HLA loss and immune escape in lung cancer evolution. *Cell* **171**, 1259-1271. e1211 (2017).
7. C. A. Stewart *et al.*, Single-cell analyses reveal increased intratumoral heterogeneity after the onset of therapy resistance in small-cell lung cancer. *Nature Cancer* **1**, 423-436 (2020).
8. Y. R. Miao *et al.*, Inhibition of established micrometastases by targeted drug delivery via cell surface–associated GRP78. *Clinical Cancer Research* **19**, 2107-2116 (2013).
9. S. Meng *et al.*, Circulating tumor cells in patients with breast cancer dormancy. *Clinical cancer research* **10**, 8152-8162 (2004).

Reviewers' Comments:

Reviewer #1:
Remarks to the Author:
Baldwin and colleagues have made substantial edits and conducted several experiments to address points raised by myself and other reviewers. I believe all of my points have been satisfactorily addressed. It will be good if authors can include the discussion point made in response 1.2 in the discussion or intro section.

Reviewer #2:
Remarks to the Author:
The authors have responded to each of the specific comments raised by the reviewers, included additional data, and revised the manuscript. However, their response to some comments are not satisfactory as they did not really answer the question. For example, engraftment differences between WT and NSG mice does not refer to the number of cells injected, but the number of cells that initiated a tumor. The statistics needs to be reviewed as well – may be by a statistician reviewer, since the authors do not acknowledge there is an issue with statistical tests.

There are many other reasons why a clone can be lost during tumor evolution. Contrary to the authors' reply, immunologic elimination is only one of the possibilities.

The 4T1 model is known to be non-responsive to single agent PD1/PDL1 blockade, contrary to the authors' observations. This needs to be clarified, especially since the authors also do not see PDL1 staining in the tumors.

Multiple papers described associations between subclonal heterogeneity and antu-tumor immune responses, including papers by Yardena Samuels, Charles Swanton, and Thomas Gajewski

Reviewer #3:
Remarks to the Author:
The authors have adequately addressed my concerns.

Reviewer #4:
Remarks to the Author:
Baldwin et al. tracked cancer cell clones during the immunoediting process and determined clonal transcriptional profiles that allow immune evasion in murine mammary tumour growth in response to immunotherapy with anti-PD1 and anti-CTLA4 and found Clonal diversity was significantly restricted by immunotherapy treatment in both primary tumours and metastases. Additionally, they found solation of immunotherapy resistant clones revealed unique and overlapping transcriptional signatures. The overlapping gene signature was associated with poor survival of basal-like breast cancer patients in two cohorts.

Overall, I found the reviewers are responsive to the reviewers' comments. But I noticed some issues that need to be addressed.

The primary concern is the data analysis part, specifically, about the reproducibility. I found the detailed description of many of the analyses is missing. Without such information, the analysis results are impossible to reproduce. Here is a list (perhaps incomplete):

1. Differential expression analysis: I assume edgeR is used. But this is never formally acknowledged. Also, any data processing is done, such as filtering, and adding small constant to all expression values to eliminate zeros?
2. For t-tests. Are the tests two-sided or one-sided? For most of the tests, especially those involves bar code counts, TPMs, log transformation is strongly recommended to make the data closer to normal distribution.
3. What software is used for survival analyses?
4. What software is used to conduct GSEA? Which gene sets are used? Why COVID-related gene sets show up in the enriched results? How do you explain that? This does not make much sense.
5. For the hierarchical clustering on Fig 4A, what software is used? Hos is distance between clusters calculated? It seems to me single linkage is used since the clusters are elongated. It is recommended to use average linkage to get better clustering result.
6. For the Volcano plot in Fig 5A, the log p-values seem really high. How were the p-values calculated? Many of these genes likely to have very high expression levels, so their significance is exaggerated. It is suggested that more attention is paid to those genes with large log fold change values.
7. Fig 7C, and D. Cox proportional hazards regression model, the label for the y-axis is missing.
8. Fig 7D, are the number at risk table correct? Since the two rows are almost the same but the difference is significant.

Baldwin *et al*.
Detailed responses and revisions

SUMMARY OF REVISIONS:

We thank the reviewers for their time in considering our manuscript. We are pleased to hear reviewers 1 and 3 are satisfied with our revisions. We have included a further discussion point into the introduction as requested by reviewer 1. We have addressed the comments of reviewers 2 and 4. We describe all changes in the point by point response below and have used track changes in the manuscript.

POINT BY POINT RESPONSES TO REVIEWERS:

Reviewer #1:

1.1 It would be good if the authors can include the discussion point made in response 1.2 in the discussion or into section.

> **Response:** We agree with the reviewer that this discussion point is worthy of inclusion in the manuscript.
>
> **Revision:** We have included the following text in the introduction, beginning at line 75:
>
> *"Intratumoural heterogeneity (ITH) has been identified as a major contributor to treatment response. Prior work in non-small cell lung cancer (NSCLC) demonstrated that neoantigen heterogeneity and tumour mutation burden more broadly is strongly associated with T cell anticancer immune responses (1, 2). Chemotherapy (3) or loss of HLA (4) have also been shown to increase ITH, which in turn was associated with treatment relapse in NSCLC. Similar results have been described in cell line models of melanoma (5), with Williams and colleagues providing recent evidence of ITH enabling clonal cooperatively and immune escape in melanoma-derived cell lines (6).*
>
> *While previous studies have been crucial to our understanding of ITH and anti-tumour immune responses and have provided undeniable evidence associating increased ITH with therapy resistance, studies of this nature have not yet been carried out in breast cancer. Furthermore, direct evidence of ITH enabling immune escape and immunotherapy resistance* in vivo *has not been reported."*

Reviewer #2:

2.1 Engraftment differences between WT and NSG mice does not refer to the number of cells injected, but the number of cells that initiated a tumor.

> **Response:** We believe that this question was answered in the prior response to reviews (Reviewer 1, point 7). To clarify, in figure 2F, figure 3D, figure 3F and the results, lines 127-130 we address the number of barcodes detected. In figure 2F, we show that NSG mice with EMT6 bearing tumours have approximately 30,000 unique barcodes detected, while only 600 unique barcodes were detected in wildtype (BALB/c) mice. In wildtype mice treated with combined immunotherapy, only 30 unique barcodes were detected.

In figure 3D and 3E, we report on the barcodes detected in the 4T1 model. Here, we show both wildtype, NSG and wildtype immunotherapy treated mice have similar numbers of unique barcodes detected (~2300).

To address the number of cells injected per model, we refer the reviewer to the results line 100, and methods line 674. Here, we state $2.5x10^6$ EMT6 cells were injected while for the 4T1 model $5x10^4$ cells were injected, results line 153 and methods line 675.

By reporting both the number of cells injected for both models, as well as reporting the number of unique barcodes detected in the primary tumours of each models, we believe we have sufficiently addressed the reviewer's comment.

2.2 *The statistics needs to be reviewed as well – may be by a statistician reviewer, since the authors do not acknowledge there is an issue with statistical tests.*

Response: We thank the reviewer for this comment and acknowledge that some of the statistical analyses were insufficient. This change has improved the rigour of the manuscript. We have consulted a specialist statistician (see acknowledgements, lines 625-626) to ensure we have carried out the correct tests and followed his advice.

Revision: We have altered the statistical tests used in figures 2F, 2G, 3F, 3F, 5C-E, 5F, 6A and 6B and supplementary figures 6B-D 8A, 8B, 12A and 12B. We have updated any reported significance values in the text. After this analysis, all results remained significant, except one result shown in Supplementary Figure 8B.

2.3 *There are many other reasons why a clone can be lost during tumor evolution. Contrary to the authors' reply, immunologic elimination is only one of the possibilities.*

Response: Reviewer two's original comments is as follows:

"T*he authors only show a decrease in clonality and do not actually prove that the eliminated clones were immunologically different leading to their elimination."*

In our response, we outlined why we strongly believe immunoediting is driving the relative enrichment of our two clones of interest (IE1 and IE2). These experiments were designed to examine differences relating to the immune response by directly comparing immunocompetent wildtype mice to severely immunocompromised (NSG) mice, and then comparing immunotherapy treated to control treated mice. We observed reproducible patterns within groups and across experiments. Clones were found to be present in both NSG and wildtype mice, but were consistently enriched in immunotherapy treated mice. Given the difference between these two experimental settings is differences in immune activity, we conclude the immune system must be responsible for the drop in clonal diversity and unique barcodes present in the lungs of immunocompetent mice, compared to immunocompromised mice.

We also undertook further work to assess the capacity for the subclones to suppress T cell activation, both with and without anti-PD1 immunotherapy. This showed that IE2 is capable of directly suppressing T cell activation *in vitro* (figure 6). Comparing severely immunocompromised mice to immunocompetent mice is an accepted approach to tease out the role of the immune system in a given model (*5, 6*). We believe this provides

strong evidence for immune elimination of clones and immune-evasive capabilities of IE1 and IE2.

Revision: To clarify our logic for the reader, we have included the following text in the discussion at line 592:

"To survive in any given system, cancer cells must utilise a number of mechanisms to avoid more than just immune destruction, as extensively reviewed in the recent work of Hanahan (7). Not only are IE1 and IE2 immune evasive, they are also highly metastatic and by their very nature must be able to grow independent of anchorage, as well as possessing abilities to engraft in both the mammary fat pad and lungs, shed from the primary tumour prior to resection and resist all other mechanisms of host anti-cancer response. We attribute the decrease in unique clones present in the lungs of control treated 4T1 tumour bearing mice compared to the primary tumour to precisely this; only some clones within the engrafted tumour possess the required abilities to be able to successfully metastasise. By making careful comparisons between controlled conditions, e.g. immunotherapy treated lungs vs non immunotherapy treated lungs, we believe we have demonstrated immunoediting occurring in vivo.*"*

2.4 *The 4T1 model is known to be non-responsive to single agent PD1/PDL1 blockade, contrary to the authors' observations. This needs to be clarified, especially since the authors also do not see PDL1 staining in the tumors.*

Response: The reviewer is correct that the 4T1 model is considered recalcitrant to single agent immunotherapy in the primary setting. However our studies are different: we examined the 4T1 model in the metastatic setting, treated with adjuvant combined immunotherapy (anti-PD-1 and anti-CTLA-4). We observe a small increase in overall survival of 4.5 days, with all mice eventually succumbing to lung metastases. The use of adjuvant immunotherapy and surgical resection in the 4T1 model to extend survival has not been previously studied in depth. As such, we don't find this small increased in survival to be contradictory to current knowledge.

2.5 *Multiple papers described associations between subclonal heterogeneity and anti-tumor immune responses, including papers by Yardena Samuels, Charles Swanton, and Thomas Gajewski*

Response: We thank the reviewer for pointing out the importance of this literature. The manuscript already contains discussions of the work of Samuels and Swanton, who first made the association between intratumoural heterogeneity (ITH), anti-tumour immune responses and treatment outcomes in melanoma and lung cancer respectively. We refer the reviewer to the discussion, line 528-538, and have provided a copy of the text below.

"Intratumoural heterogeneity (ITH) has previously been associated with resistance to immunotherapy in melanoma and lung cancer, with higher ITH being associated with resistance to immunotherapy (1, 4, 5). McGranahan and colleagues postulated that this was due to improved T cell killing of tumours with clonal neo-antigens. A non-mutually exclusive explanation is that clonal tumours are less likely to contain cancer cells with a pre-existing resistance mechanism to immunotherapy. These findings refine the concept of cancer

immunoediting, demonstrating that there are clonal populations of cancer cells with variable resistance to the immune system. Based on their phenotype, these clones are either enriched or depleted by an active immune system and immunotherapy."

We acknowledge we mistakenly excluded some work from Gajewski and colleagues and thank the reviewer for bringing this to our attention. We have now referenced this work in the manuscript (see revision below).

The works of Gajewski, Swanton and Samuels are undeniably crucial to our conceptual understanding and demonstrate associations between ITH and treatment response. Our paper is unique in that it provides direct evidence for ITH enabling immune escape *in vivo* by using DNA barcoding to trace individual cancer cell clones during metastasis and after treatment with immunotherapy. This approach has not been taken before, nor has this level of evidence been provided.

Revision: In addition to the existing references to some of this work in the discussion, we have added the following to the introduction, line 75-87.

"Intratumoural heterogeneity (ITH) has been identified as a major contributor to treatment response. Prior work in non-small cell lung cancer (NSCLC) demonstrated that neoantigen heterogeneity and tumour mutation burden more broadly, is strongly associated with T cell anticancer immune responses (1, 2). Chemotherapy (3) or loss of HLA (4) have also been shown to increase ITH, which in turn was associated with treatment relapse in NSCLC. Similar results have been described in cell line models of melanoma (5), with Williams and colleagues providing recent evidence of ITH enabling clonal cooperatively and immune escape in melanoma-derived cell lines (6).

While these previous studies have been crucial to our understanding of ITH and anti-tumour immune responses and have provided undeniable evidence associating increased ITH with therapy resistance, studies of this nature have not yet been carried out in breast cancer. Furthermore, direct evidence of ITH enabling immune escape and immunotherapy resistance in vivo *has not been reported."*

<u>Reviewer #4:</u>

4.1 Differential expression analysis: I assume edgeR is used. But this is never formally acknowledged. Also, any data processing is done, such as filtering, and adding small constant to all expression values to eliminate zeros?

Response: The reviewer is correct, EdgeR has been used for DEG analysis. We direct the reviewer to the methods, line 778, where we state

"Differentially expressed genes and repeat elements were defined with EdgeR with FDR<0.01 (8)"

Further, read counts from RSEM were processed with the default edgeR workflow that performs DE analysis with genewise negative binomial generalized linear models through function glmQLFTest (*9*). Genes that were present with less than 10 reads across less than 3 samples per group were eliminated from the analysis. The model involved comparison of IE samples to

differences between NT and 4T1 cell lines. We apologies for the lack of detail here and have modified the methods accordingly.

Revision: For clarity, we have added the following to the results, at line 291.

"Differential gene expression analysis was carried out using EdgeR."

We have added the following to the methods, at line 829-830

"EdgeR uses genewise negative binomial generalised linear models through the function glmQLFTest (9). Genes that with less than 10 reads across three samples per group were omitted from the analysis. The model involved comparison between IE samples, NT samples and the bulk 4T1 cell line."

4.2 For t-tests. Are the tests two-sided or one-sided? For most of the tests, especially those involves bar code counts, TPMs, log transformation is strongly recommended to make the data closer to normal distribution.

Response: This is a valuable point raised by reviewer 4 and is similar to a comment from reviewer 2 (2.2). We agree that a t-test is not appropriate for analysis of some of our data as it is not normally distributed. We have consulted a specialist statistician and upon their recommendation have fitted more appropriate models for non-normal data (generalised linear model, GLM) and carried out post hoc tests for significance, accounting for multiple comparisons.

Revision: Please see revision for comment 2.2

4.3 What software is used for survival analyses?

Response: For the survival analysis, we used a number of resources and packages. We direct the reviewer to the methods, beginning at line 785, where we state:

"To assess the clinical relevance of our isolated immune evasion clones, we assessed the association between the gene signatures derived from our bulk RNA-Sequencing studies with the overall survival of basal (PAM50) breast cancer patients from the METABRIC and The Cancer Genome Atlas (TCGA; https://www.cancer.gov/tcga) cohorts. Mouse gene signatures were first converted to human orthologs using the biomaRt package (10). Shared up-regulated genes across both immune evasion clones IE1 and IE2 were then filtered, and only genes detected in each expression cohort were considered. For each tumour from the bulk cohort, signature scores were computed based on the average expression of the top 25 genes ranked by log fold change. Patients were then stratified based on the signatures scores into the top 30%, middle 40% and bottom 30% groups. Survival curves were generated using the Kaplan Meier method with the 'survival' package in R (https://cran.r-project.org/package=survival). The Cox proportional hazards model was used to compute Hazard Ratios. We assessed the significance between groups using the log-rank test statistics."

4. What software is used to conduct GSEA? Which gene sets are used? Why COVID-related gene sets show up in the enriched results? How do you explain that? This does not make much sense.

Response: We thank the reviewer for pointing out this omission. We have added to the methods, as described below.

We only find one significant COVID-related gene set in our analysis. This geneset was derived from a paper by Blanco-Melo and colleagues, describing immune dysregulation induced by SARS-CoV-2 infected lung epithelial cells (*11*). To us, this suggests an immune-modulating role of the genes we identified that were common to both of our subclones. We refer the reviewer to the results, lines 394-398, where we state

"Only two gene sets had significant p values when multiple testing was considered, these were the HOXA5 gene set mentioned previously and a COVID-19 related gene set. Although not significant, there were several additional COVID-19 related gene sets from the same recent publication identified in the overlapping upregulated gene list suggesting an immune related role of these genes (11)."

Revision: We have added the following to the methods, beginning at line 801.

"Gene set enrichment analysis (GSEA) was carried out using the GSEA desktop app (4.1.0) and DEGs generated as described above. GSEA was run using preranked list of significantly differentially expressed genes, ranked by log fold change. The molecular signatures database (MsigDB v7.5.1) hallmark and curated (C2) gene sets were used for analysis."

4.5 For the hierarchical clustering on Fig 4A, what software is used? Hos is distance between clusters calculated? It seems to me single linkage is used since the clusters are elongated. It is recommended to use average linkage to get better clustering result.

Response: Figure 4A was made with package Pheatmap, v.1.0.12 with default parameters as follows. Clustering distances for rows and columns (clustering_distance_cols, clustering_distance_rows) were "euclidean" and clustering linkage (clustering_method) was "complete" and not "single". We believe complete linkage is appropriate in this context and behaves similarly to average linkage. We thank the reviewer for drawing our attention to these omitted details and have added the methods as below.

Revision: We have added the following to the methods at line 737

"Pheatmap (v1.0.12) was used with default parameters. Clustering distances for rows and columns were euclidean and complete clustering linkage."

4.6 For the Volcano plot in Fig 5A, the log p-values seem really high. How were the p-values calculated? Many of these genes likely to have very high expression levels, so their significance is exaggerated. It is suggested that more attention is paid to those genes with large log fold change values.

Response: The p-values were generated with edgeR's glmQLFTest with default parameters. After elimination of the lowly expressed genes (less than 10 reads in less than 3 samples per group), the model compared IE values to differences between NT and 4T1 cells. The increase in log p-values is most likely due to batch correction of normal values by the used model. We have added the detailed description of the batch correction to the methods.

Revision: See comment 4.1 for detailed addition to the methods section.

4.7. Fig 7C, and D. Cox proportional hazards regression model, the label for the y-axis is missing.

Response: We thank the reviewer for pointing out this omission.

Revision: We have added y-axis labels to figure 7C and 7D.

4.8. Fig 7D, are the number at risk table correct? Since the two rows are almost the same but the difference is significant.

Response: We thank the reviewer for noticing this mistake in Figure 7D of our manuscript. Upon inspection, we identified that this were due to a previously reported errors when plotting risk tables using R packages for survival analysis (reported on GitHub: https://github.com/kassambara/survminer/issues/36 or https://github.com/kassambara/survminer/issues/37). This is purely related to plotting and does not impact the findings of this section. To confirm this, we inspected the raw data behind the risk table in Figure 7D (below), where numbers at risk (n.risk column, see image below) correctly match the survival trends shown in the Kaplan Meier curves above. We have removed the risk tables from these figures to avoid any confusion.

CELLTYPE=bot30%

time	n.risk	n.event	survival	std.err	lower 95% CI	upper 95% CI
224	35	1	0.971	0.0282	0.918	1
524	29	1	0.938	0.0427	0.858	1

CELLTYPE=top30%

time	n.risk	n.event	survival	std.err	lower 95% CI	upper 95% CI
255	35	1	0.971	0.0282	0.9178	1.000
266	34	1	0.943	0.0392	0.8690	1.000
548	30	1	0.911	0.0489	0.8204	1.000
639	24	1	0.873	0.0598	0.7637	0.999
754	22	1	0.834	0.0690	0.7088	0.981
785	21	1	0.794	0.0763	0.6577	0.959
1556	13	1	0.733	0.0917	0.5736	0.937
1692	10	1	0.660	0.1079	0.4787	0.909
3472	2	1	0.330	0.2394	0.0795	1.000

References

1. N. McGranahan *et al.*, Clonal neoantigens elicit T cell immunoreactivity and sensitivity to immune checkpoint blockade. *Science* **351**, 1463-1469 (2016).
2. N. A. Rizvi *et al.*, Mutational landscape determines sensitivity to PD-1 blockade in non-small cell lung cancer. *Science* **348**, 124-128 (2015).
3. C. A. Stewart *et al.*, Single-cell analyses reveal increased intratumoral heterogeneity after the onset of therapy resistance in small-cell lung cancer. *Nature Cancer* **1**, 423-436 (2020).
4. N. McGranahan *et al.*, Allele-specific HLA loss and immune escape in lung cancer evolution. *Cell* **171**, 1259-1271. e1211 (2017).
5. Y. Wolf *et al.*, UVB-Induced Tumor Heterogeneity Diminishes Immune Response in Melanoma. *Cell* **179**, 219-235.e221 (2019).
6. J. B. Williams *et al.*, Tumor heterogeneity and clonal cooperation influence the immune selection of IFN-γ-signaling mutant cancer cells. *Nature Communications* **11**, 602 (2020).
7. D. Hanahan, Hallmarks of Cancer: New Dimensions. *Cancer Discovery* **12**, 31-46 (2022).
8. M. D. Robinson, D. J. McCarthy, G. K. Smyth, edgeR: a Bioconductor package for differential expression analysis of digital gene expression data. *Bioinformatics* **26**, 139-140 (2009).
9. A. T. Lun, G. K. Smyth, No counts, no variance: allowing for loss of degrees of freedom when assessing biological variability from RNA-seq data. *Statistical Applications in Genetics and Molecular Biology* **16**, 83-93 (2017).
10. S. Durinck, P. T. Spellman, E. Birney, W. Huber, Mapping identifiers for the integration of genomic datasets with the R/Bioconductor package biomaRt. *Nature Protocols* **4**, 1184-1191 (2009).
11. D. Blanco-Melo *et al.*, Imbalanced Host Response to SARS-CoV-2 Drives Development of COVID-19. *Cell* **181**, 1036-1045.e1039 (2020).
